# Genome-wide identification of rice *CXE* gene family and mining of alleles for potential application in rice improvement

**DOI:** 10.3389/fpls.2024.1435420

**Published:** 2024-10-17

**Authors:** Jinguo Zhang, Xinchen Wang, Guohui Dou, Dezhuang Meng, Chenghang Tang, Jiaqi Lv, Nansheng Wang, Xingmeng Wang, Jianfeng Li, Yaling Bao, Guogeng Zhang, Tao Huang, Yingyao Shi

**Affiliations:** ^1^ School of Agronomy, Anhui Agricultural University, Hefei, China; ^2^ Institute of Crop Sciences, Chinese Academy of Agricultural Sciences, Beijing, China

**Keywords:** rice, carboxylesterase, gene-CDS-haplotype (gcHap) diversity, yield traits, biological stress

## Abstract

Carboxylesterases (CXE, EC 3.1.1.1), a class of hydrolases with an α/β folding domain, play important roles in plant growth and development and stress response. Here, we identified 32, 63, 41, and 45 *CXE* genes in Oryza sativa Japonica (Nipponbare), Oryza sativa Indica (93-11), Oryza sativa Indica (*Xian*-1B1 var.IR64), and Oryza sativa Japonica (*Geng*-sbtrp var.ChaoMeo), respectively. Then, we analyzed the chromosomal location, physical and chemical properties, subcellular localization, collinearity, and selection pressure of *CXE* genes in four rice varieties. We also analyzed the functional interaction network, cis-regulatory elements, evolutionary relationship, and protein tertiary structure, and performed gene expression profiling and qPCR verification under abiotic stress, as well as diversity analysis of 3010 gene-CDS-haplotype (gcHap) rice samples, aiming to understand the potential function of the 32 *OsCXE* genes. Our results indicated that fragment replication is the main reason for amplification of the *CXE* gene family in rice, and the gene family has undergone strong purification selection. *OsCXE3.1, OsCXE3.2, OsCXE3.3, OsCXE5.1*, and *OsCXE7.3* may be used to improve the tolerance of rice to abiotic stress. *OsCXE* play important roles in rice population differentiation and improvement, and the major gcHaps at most *OsCXE* locus are significantly associated with yield traits. Therefore, natural variations of most *OsCXE* locus have great potential value for improvement of rice productivity.

## Introduction

Carboxylesterases (CXE, EC 3.1.1.1) are a superfamily of proteins encoded by the supergene family that have α/β folding domains and can catalyze the hydrolysis of ester and amide compounds (Zhang et al., 2017). This family of proteins contains eight β-folds and a conserved core region consisting of α-helices and irregular rings. CXE comprise three glycines and catalytic ternary structures (serine, aspartate, and histidine) of the oxygen anion gap of the stable substrate-enzyme intermediates, as well as conserved hormone-sensitive domains HGG and GXSXG. At present, 20, 39, 40, and 72 CXE have been identified in Arabidopsis thaliana (Marshall et al., 2003), housefly (Feng et al., 2018), pear (Qi et al., 2023), and sea island cotton (Rui et al., 2022), respectively. CXE are involved in regulating the activity of a variety of substances, and various biological processes such as nerve formation and development and degradation of toxic substances, pheromones, and hormones in animals, and the formation of drug resistance in insects (Rasmussen et al., 2018). In plants, CXE play important roles in the detoxification of herbicides, activation of hormone signaling substances, and response to biotic stress (Griffiths et al., 2007). At present, some CXE have been preliminarily verified in plants. For instance, Nthsr203J in tobacco (Pontier et al., 1998), PepEST in sweet pepper (Ko et al., 2016), and *BdCXE29* in *Brachystis dipaniculata* (Schmeitzl et al., 2016) were all verified to be involved in the interaction between plants and pathogens. Tomato Lehsr203J can hydrolyze methyl jasmonate and methyl salicylate into active jasmonic acid (JA) and salicylic acid (SA), releasing signaling molecules to regulate plant growth and development (Stuhlfelder et al., 2002). Arabidopsis thaliana *AtCXE12* is involved in herbicide metabolism and has detoxification effects on plant toxins (Gershater et al., 2007). Radiata pine PrMC3 plays a potential role in the process of programmed cell death (Walden et al., 1999). Sea island cotton *GBCXE49* is probably involved in alkaline stress response (Rui et al., 2022). The above findings demonstrate that CXE play important roles in regulating herbicide activity, hormone signaling activation, and stress response in plants.

Rice is one of the most important food crops in the world and also a cereal model plant with great intraspecific genetic diversity (Li and Rutger, 2000; Yu et al., 2003). The completion of sequencing of 3010 different rice materials (3KRG) from 89 countries around the world has provided a large amount of genetic information of rice (Wang et al., 2018). On the basis of 3KRG, Zeng et al. analyzed the potential of applying *OsGLR* genes in rice improvement by combining haplotype data (Zeng et al., 2023). Cheng et al. investigated the role of *OsBES1-4* gene in regulating the grain size of rice by combining the haplotype data (Cheng et al., 2023)gene functions. However, it remains challenging to identify and mine elite alleles from rice germplasm resources to improve specific target traits in rice. At present, there have been rapid advances in studies of rice functional and population genomics, but these advances have not been widely applied to the development of more efficient breeding techniques. This is due to the fact that there has been incomplete information on the phenotypic effects of the cloned genes, as most experiments were conducted in the laboratory without full consideration of the breeding target environments, that is, the influence of environment interaction genotypes and genetic backgrounds of most cloned rice genes on some important agronomic traits remains largely unknown (Zhang et al., 2021). Secondly, before research on a specific gene of interest, researchers should check whether a similar allele has been identified and fixed in commercial varieties, and if a gene has been worked on for decades, it is unlikely to bring a sudden increase in crop yield (Khaipho-Burch et al., 2023). Finally, there are abundant natural allelic variations in most gene locus in rice. The function of many CXE in rice is still unknown. Hence, it is an important challenge to obtain the information needed for the breeding of these genes of unknown functions without cloning.

In this study, we adopted a comprehensive approach to identify 32 *OsCXE gene* from the rice (Nipponbare) genome, and studied the gene structure, phylogeny, and expression patterns to lay a foundation for further revealing the function of the rice *CXE* gene family. We also conducted a comprehensive population genetic analysis of the gene-cds-haplotype (gcHap) diversity of rice *OsCXE*, providing important information for future development of gene locus required for new breeding techniques.

## Materials and methods

### Identification of *CXE* family genes in rice

To identify the *CXE* family genes in rice, we accessed in the Ensembl database of the Ensembl Plants (https://plants.ensembl.org/index.html access) on September 26^th^, 2023 to download the genome-wide data of Oryza sativa Japonica (Nipponbare), Oryza sativa Indica (93-11), Oryza sativa Indica(*Xian*-1B1 var.IR64), and Oryza sativa Japonica (*Geng*-sbtrp var.ChaoMeo),the article represents them respectively as Os, 93-11, IR64, and ChaoMeo. The Hidden Markov Model (HMM) of PF07859 was downloaded from the pfam database (accessed September 26, 2023; http://pfam.xfam.org/) (Chen et al., 2020). Then, the Simple Hmm Search function of TBtools software was used to obtain the potential *CXE* genes and protein sequences (Chou and Shen, 2010). Then, the NCBI web site (https://www.ncbi.nlm.nih.gov/Structure/bwrpsb/bwrpsb.cgi) was used to analyze the genetic structure domain, and by combining the functional annotation, a comprehensive analysis was conducted for further screening to determine the members of the family, which were renamed according to their position on the chromosome. The online tools Cell-PLoc 2.0 (http://www.csbio.sjtu.edu.cn/bioinf/euk-multi-2/access) on September 27^th^, 2023 was used for subcellular localization prediction (Studer et al., 2021). SWISS-MODEL (https://swissmodel.expasy.org/interactive0) (September 26, 2023) was used for the prediction of CXE protein tertiary structure (Letunic and Bork, 2016). Moreover, the Protein Paramter Calc function of TBtools software was used to analyze the physicochemical properties of rice *CXE* genes, such as isoelectric point (Pi) and molecular weight (Da) (Chou and Shen, 2010).

### Evolution analysis of *CXE* genes

To study the evolutionary relationship of *CXE* genes in different rice varieties, we obtained the *CXE* genes from four rice varieties and Arabidopsis thaliana, and constructed a phylogenetic tree by the Neighbor-joining method in MEGA11 software. Then, the online mapping site iTOL (accessed September 26^th^, 2023; https://itol.embl.de/) was used to beautify the phylogenetic tree (Letunic and Bork, 2016).For more detailed steps, refer to Cui article published in BMC Molecular Plant (Rui et al., 2022).

### Analysis of *cis*-regulatory elements, conserved motifs, conserved domains, and promoter regions of CXE family proteins

After downloading of the gene structure annotation files from the website of Ensembl Plants, visualization was performed using the Gene Structure View (Advanced) function in TBtools software (Chen et al., 2020). Conserved CXE protein sequence analysis was performed by the MEME (https://meme-suite.org/meme/tools/meme; September 27^th^, 2023) and simple TBtools software MEME wrapper functions (Bailey et al., 2015), with the number of motifs being set as 10. The conserved domain was analyzed by NCBI CD retrieval function and visualized by Tbtools. *Cis*-regulatory element analysis was performed using the Gtf/Gff3 sequence extract function in TBtools to extract the upstream 2000 bp of CDS and the promoter sequence of *CXE* genes. The results obtained were submitted to PlantCARE database (http://bioinformatics.psb.ugent.be/webtools/plantcare/html/access) on September 26^th^, 2023 to analyze the *cis*-regulatory elements in the promoter regions (Lescot, 2002). The results were filtered based on the information in the table and the file was retained for viewing, which was then visualized using the Simple BioSequence Viewer function of TBtools software (Chen et al., 2020).

### Collinearity analysis of rice *CXE* genes

First, the gff files and DNA files of the target genomes were downloaded from the Ensembl Plants website, and then TBtools was used to obtain the gene pair files for each rice species (Os - 93-11, Os - IR64, Os - ChaoMeo, ChaoMeo - IR64, 93-11 - ChaoMeo, 93-11 - IR64).The collinearity of duplicate gene pairs in four rice species (Nipponbare, 93-11, *Xian*-1B1 var. IR 64, *Geng*-sbtrp var. ChaoMeo) was analyzed using the One Step MCScanX and Advance Circos functions of TBtools. Finally, the collinearity results were visualized using chromosome length files and genome comparison files.

### Gene expression profiling based on RNA-seq

From the RNA-seq database (PPRD, http://ipf.sustech.edu.cn/pub/plantrna/; September 26th, 2023), the rice *CXE* gene RNA-seq expression data were retrieved (as FPKM values, Transcripts per kilobase read per million maps) (Yu et al., 2022). Data of multiple tissue sites, different development times, and under abiotic stress conditions were analyzed using average FPKM values in the presence of multiple libraries. Tbtools software was used to create heat maps to visually represent these expression patterns (Chen et al., 2020).

### Material handling

Rice seeds (Nipponbare) were disinfected with 3% sodium hypochlorite for 30 min, germinated at 28°C for 3 d, and transplanted into a hydroponic box containing Hoagland nutrient solution. In an intelligent light temperature incubator (under normal conditions: 28°/12 h at day, 26°/12 h at night, humidity 80%, light intensity 3000 lux). The seedlings were treated with low temperature (4°C), high temperature (42°C), high salt (200 mmoL/L), drought (PEG20%), and two different hormones (100 μmol/L ABA and 100 μmol/L MeJA), respectively. Rice leaves were taken at 0, 4, 8, 12 and 24 h, respectively, and immediately placed in liquid nitrogen and stored at –80°C to extract total RNA.

### Real-time fluorescence quantitative PCR analysis

The obtained sample was ground in liquid nitrogen with a mortar and pestle. The TaKaRa MiniBEST Plant RNA Extraction Kit (TaKaRa, Japan) was used for the extraction of RNA. Reverse Transcription Kit (TAKARA, Japan) was used to perform reverse transcription of the obtained cDNA, and gene expression was analyzed by qRT-PCR.Primers for the screened *OsCXE3.1, OsCXE3.2, OsCXE3.3, OsCXE5.1* and *OsCXE7.3* were designed, and relative quantitative expression was normalized to the reference gene *OsActin1* (LOC_Os03g61970) (Cui et al., 2020). The related information of primers is shown in **Supplementary Table S1**. Real-time fluorescence quantitative detection was performed using LightCyler 96 quantitative PCR instrument. The amplification system was 20 μL, and the cDNA was 2 μL. The positive and negative primers were 0.8 μL; AceQ Universal SYBR qPCR Master Mix was 10 μL; and ddH_2_O was 6.4 μL. The program was set as predenaturation at 95°, 5 min; denaturation at 95°, 10 s, annealing and extension at 60°, 30 s, in a total of 40 cycles. Three biological replicates and three technical replicates were performed. The expression of each gene was analyzed by the 2^-ΔΔct^ method. WPS 2023 software was used for statistical analysis of data, and GraphPad Prism 8 software was used for variance analysis and drawing pictures.

### GcHap of *OsCXE* and gcHap diversity among modern and indigenous rice varieties

We used Shannon’s Equity (*E_H_*)to assess gcHap diversity at *OsCXE* locus across different rice populations. For each gene, Nei genetic diversity (*I_Nei_*) estimates the genetic difference between two populations using gcHap data and was used to measure genetic differentiation between populations (Nei, 2019; Zhang et al., 2021). To understand how modern breeding over the past few decades has affected the gcHap diversity of *OsCXE* genes in rice, we collected detailed information on a total of 3010 3KRG rice materials.Of these, 732 were identified as *Xian* landraces (LANs-*Xian*), 358 were identified as *Geng* landraces (LANs-*Geng*),identifed as modern *Xian* varieties (MVs-*Xian*),and 139 were identified as modern *Geng* varieties (MVs-*Geng*). First of all, we downloaded the gcHap data of *OsCXE* genes from RFGB (https://www.rmbreeding.cn/Index access) on September 26th, 2023. Then, based on the R script, the drift frequency of the major gcHaps of each *OsCXE* gene between the modern varieties (MVs) and the local varieties (LANs) was calculated (Zeng et al., 2023). The gcHap distributions of modern *Xian* and *Geng* varieties and their respective local varieties were then compared. At the same time, the loss and new emergence of gcHaps in modern *Xian/Geng* varieties were also analyzed. Finally, GraphPad Prism 8 software was used to plot the above data.

### Extraction of major gcHap phenotypes of *OsCXE* genes

First, we collected phenotypic data on 15 agronomic traits from 3010 cultivated rice cultivars in Asia. The study examined 15 agronomic traits, including Days to Heading (DTH, day), Plant Height (PH, cm), Flag Leaf Length (FLL, cm), Flag Leaf Width (FLW, cm), Panicle Number (PN, count), Panicle Length (PL, cm), Culm Number (CN, count), Culm length (CL, cm), Grain Length (GL, mm), Grain Width (GW, mm), Grain Length/Width Ratio (GLWR, ratio), Thousand Grain Weight (TGW, g), Leaf Rolling Index (LRI, %), Seedling Height (SH, cm), and Ligule Length (LL, mm). The phenotypic data for 15 rice traits were downloaded from the RFGB website (accessed September 26^th^, 2023; https://www.example.comIndex/). Next, R script was used to obtain the major gcHaps of all *OsCXE* genes (Zeng et al., 2023). Finally, R scripts were used to correlate major gcHaps with these agronomic traits in 3,010 rice materials. Significance was calculated using one-way analysis of variance, and significance among major gcHaps was compared using Tukey multiple comparison method. The layout of the images was performed in Adobe Illustrator 2023 software.

### Construction of CDS haplotype (gcHap) network of *OsCXE* genes

First, a haplotype of *OsCXE* genes (gcHap) was constructed using R script pegas (Paradis, 2010). A network of gcHaps for each *OsCXE* gene was generated using a statistical reduction algorithm, which first connects the most closely related haplotypes by a minimum number of mutations (Templeton et al., 1992). The layout of the images was performed in Adobe Illustrator 2023 software.

## Results

### Identification and characterization of the *CXE* family genes in rice

In order to identify the *CXE* family genes in rice, a hidden Markov model (PF07859) was used for sequence alignment by Hmmer comparison method, and the genes that were incomplete and did not contain the Abhydrolase_3 domain were removed to identify the family members. As a result, in the Oryza sativa Japonica (Nipponbare), Oryza sativa Indica (93-11), Oryza sativa Indica (*Xian*-1B1 var.IR64), and Oryza sativa Japonica (*Geng*-sbtrp var.ChaoMeo), 32, 63, 41, and 45 *CXE* genes were predicted and renamed according to their chromosomal positions (**Supplementary Table S2**). These 181 *CXE* genes were distributed on all chromosomes except for chromosome 10, with the largest number of genes located on chromosome 7 (**Supplementary Table S2**).

The physical and chemical properties of the *CXE* genes were analyzed, including the protein length, Molecular Weight (Da), isoelectric point (pI), instability coefficient, hydrophilic index, and fat index (**Supplementary Table S2**). The results showed that in the Oryza sativa Japonica (Nipponbare), members of this family encoded proteins ranging from 321 aa (*OsCXE9.3)* to 460 aa (*OsCXE7.7*) in length. The Da ranged from 34163.74 Da (*OsCXE8.3*) to 49700.11 Da (*OsCXE7.7*), and the pI ranged from 4.95 (*OsCXE7.3*) to 9.37 (*OsCXE9.1*), The adipose index ranged from 70.07 (*OsCXE7.7*) to 96.18 (*OsCXE11.1*). Among the *OsCXE* genes, 23 genes had an instability coefficient greater than 40, indicating that most CXE members were unstable, and 22 genes had a hydrophilic index lower than 0, indicating that most of them are hydrophilic proteins. *93-11CXE*, *ChaoMeoCXE*, and *IR64CXE* proteins were encoded by family members ranging in length from 110 aa (*93-11CXE5.1*) to 685 aa (*93-11CXE7.13*), 149 aa (*ChaoMeoCXE9.3*) to 1449 aa (*ChaoMeoCXE7.1*), and 147 aa (*IR64CXE9.7*) to 1338 aa (*IR64CXE7.9*). The Da ranged from 12173.82 Da (*93-11CXE5.1*) to 74913.49 Da (*93-11CXE7.13*), 16560 Da (*ChaoMeoCXE9.3*) to 156279.01 Da (*ChaoMeoCXE7.1*), and 15725.86 Da (*IR64CXE9.7*) to 144254.64 Da (*IR64CXE7.9*). The pI ranged from 4.84 (*93-11CXE7.3*) to 9.76 (*93-11CXE8.3*), 4.96 (*ChaoMeoCXE8.4*) to 9.71 (*ChaoMeoCXE9.1*), and 4.86 (*IR64CXE1.1*) to 9.73 (*IR64CXE7.4*). The adipose-index ranged from 67.67 (*93-11CXE1.4*) to 97.02 (*93-11CXE11.3*), 70.07 (*ChaoMeoCXE7.5*) to 94.4 (*ChaoMeoCXE1.1*), and 70.94 (*IR64CXE9.2*) to 95.85 (*IR64CXE12*). Most family member proteins were unstable and hydrophilic proteins. Subcellular localization prediction showed that most rice *CXE* family genes were located on the cell membrane and chloroplasts (**Supplementary Table S2**).

### Analysis of the evolutionary relationship of rice *CXE* family members

**Figure 1:**
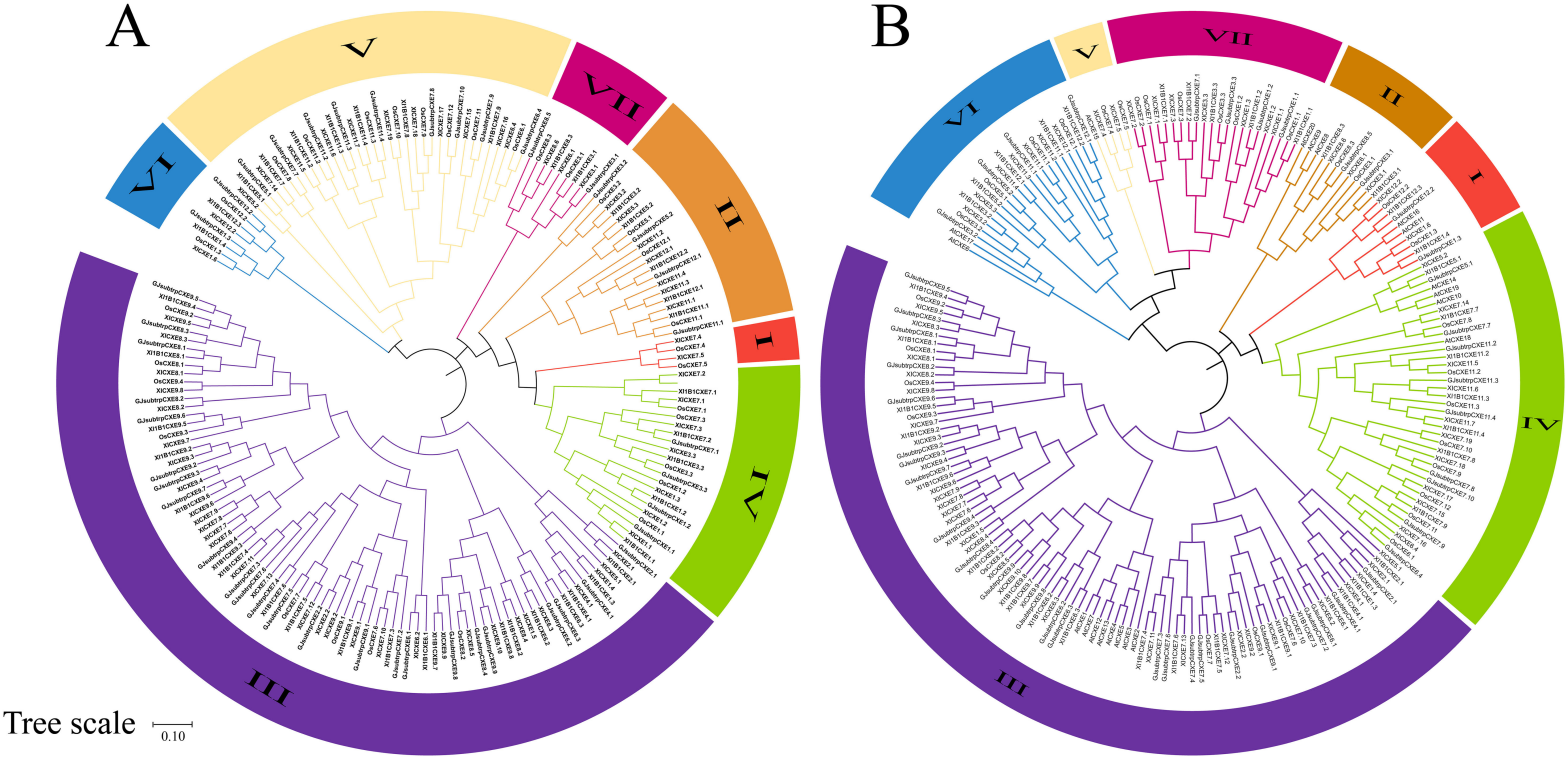
Phylogenetic trees of CXE family members. **(A)** Phylogenetic tree of four rice species; **(B)** Phylogenetic tree of four rice and *Arabidopsis Thaliana*.

**Figure 2:**
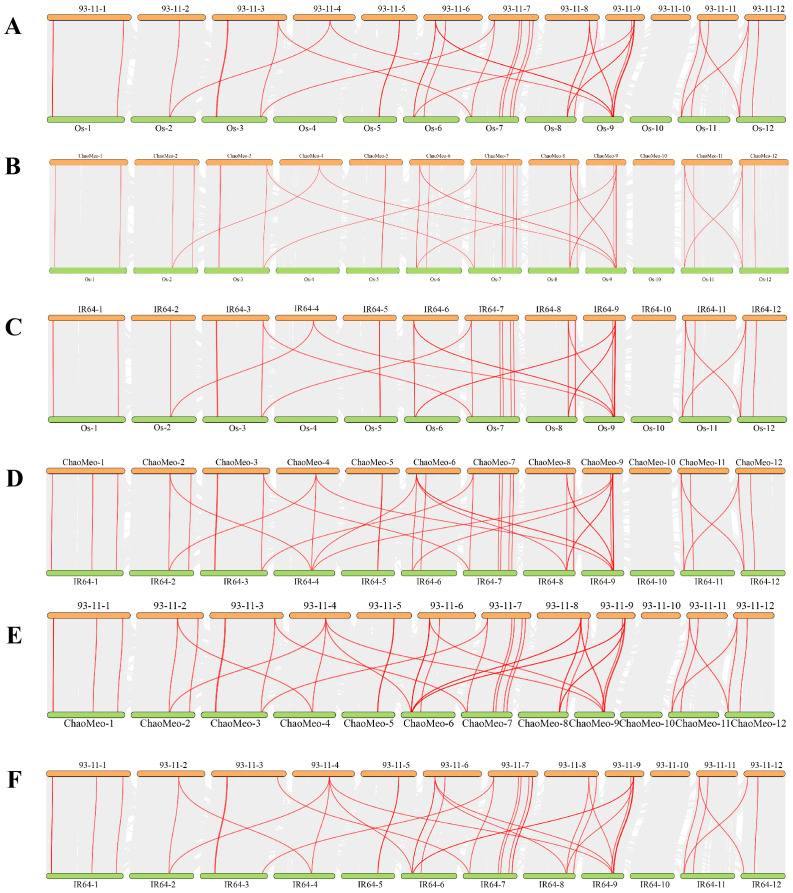
Collinearity of CXE genes in rice. **(A)** Collinearity between Os and 93-11. **(B)** Collinearity between Os and ChaoMeo. **(C)** Collinearity between Os and IR64. **(D)** Collinearity between ChaoMeo and IR64. **(E)** Collinearity between 93-11 and ChaoMeo. **(F)** Collinearity between 93-11 and IR64.

In order to study the evolutionary relationship between CXE proteins, the CXE family members in four rice species and *Arabidopsis thaliana* were compared. MEGA11 software was used to investigate the evolutionary relationships among the sequences and generate the phylogenetic tree (**Figure 1B**). The CXE family proteins were divided into seven clades (clades I–VII) with different gene numbers (**Figure 1A**). Clades III and IV contained a large number of genes (eight *OsCXE*). The *CXE* genes were present in all six subpopulations of *A. thaliana* except for clade VII, which did not contain any *AtCXE* gene (**Figure 1B**). To evaluate the evolutionary relationships of *CXE* family genes in rice, we analyzed the phylogenetic relationships of *CXE* family genes using the MCScanX toolkit. The results showed that *OsCXE* exhibited 42 collinear genes with *93-11CXE*, 41 collinear genes with *ChaoMeoCXE*, and 40 collinear genes with *IR64CXE*. Moreover, *93-11CXE* exhibited 42 collinear genes with *IR64CXE* and 48 collinear genes with *ChaoMeoCXE*, *ChaoMeoCXE* and *IR64CXE* had 45 pairs of collinear genes (**Figure 2**). The collinearity of *CXE* genes in rice indicated a close evolutionary relationship between japonica and indica. Gene replication events are crucial for the formation of gene families. Therefore, we studied the replication events of *CXE* family genes in rice. Our analysis revealed three collinear gene pairs in *OsCXE*, 12 collinear gene pairs in *ChaoMeoCXE*, 8 collinear gene pairs in *93-11CXE*, and 10 collinear gene pairs in *IR64CXE* (**Supplementary Figure S1**). Further analysis showed that the replication mode was fragment replication, indicating that fragment replication has played an important role in the expansion of CXE family in rice.

### Ka/Ks analysis of rice CXE family members

**Figure 3:**
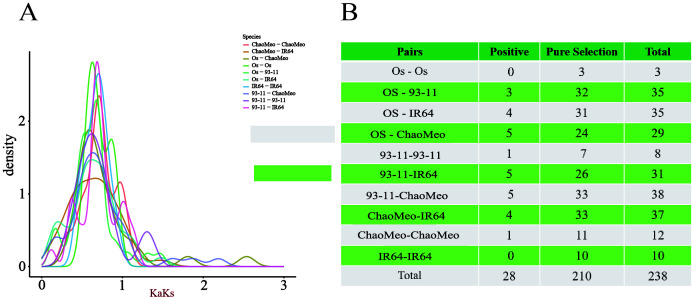
Selection pressure (Ka/Ks) analysis. **(A)** Selection pressure diagram. **(B)** Prediction of the number of genes in different combinations of four rice species,Where positive indicates Ka/Ks> 1 and pure selection indicates Ka/Ks<1.

Ka/Ks< 1 represents purification selection, indicating that natural selection has eliminated harmful mutations and left the protein unchanged. Ka/Ks > 1 indicates positive selection, which indicates that natural selection acts on the change of protein and causes the rapid disappearance of mutation site in the population to accelerate gene evolution. Ka/Ks = 1 denotes neutral selection, indicating that natural selection has no effect on mutation (Hurst, 2002). We determined 10 combinations of four rice species (Os-Os, Os-93-11, Os-IR64, Os-ChaoMeo, 93-11-93-11, 93-11-IR64, 93-11-ChaoMeo, ChaoMeo-IR64, ChaoMeo-ChaoMeo, and IR64-IR64). The results (**Figure 3**) showed that 28 gene pairs had Ka/Ks > 1 (**Figure 3B**); 210 gene pairs had Ka/Ks< 1, and most gene pairs had 0.5< Ka/Ks< 0.99 (**Figure 3A**). These results indicated that the *CXE* family genes have undergone strong purification selection in rice.

### Analysis of conserved motifs, domains, and gene structure of *OsCXE*

**Figure 4:**
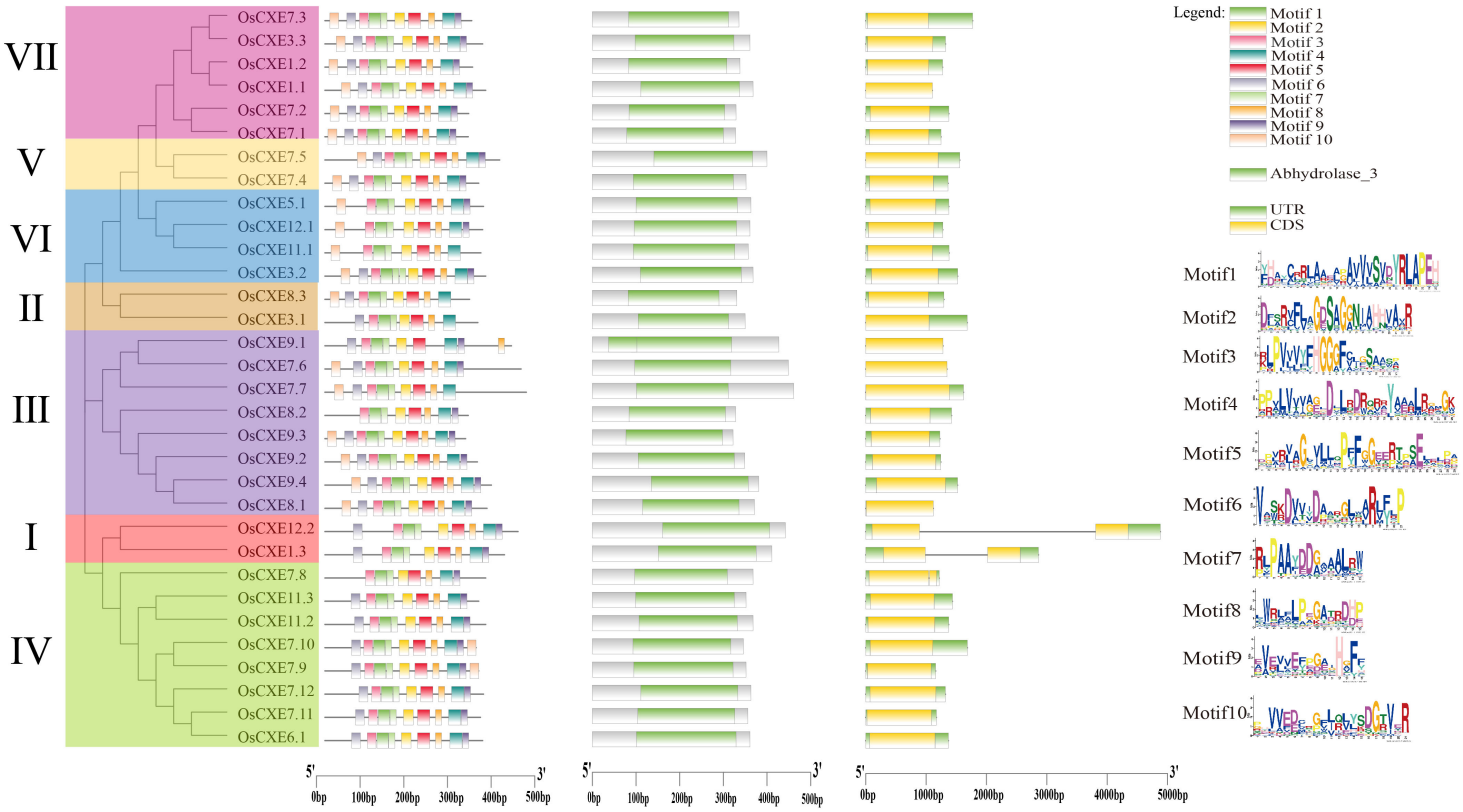
From left to right, phylogenetic tree,conserved motif,conserved domainc and gene structure analysis of 32 *OsCXE* (Nipponbare).

Phylogenetic analysis revealed that *OsCXE* can be divided into seven clades based on their amino acid sequences (**Figure 4**). First of all, in order to predict the evolution and functional diversity of *OsCXE*, exon-intron analysis of 32 *OsCXE* gene sequences was performed using TBtools. The results showed that most *OsCXE* genes had two exons and one intron, and only four genes (*OsCXE1.1*, *OsCXE9.1*, *OsCXE7.6*, and *OsCXE8.1*) had no exon. Three genes (*OsCXE7.5*, *OsCXE3.1*, and *OsCXE7.7*) had only one exon, and three genes (*OsCXE1.3*, *OsCXE12.2*, *OsCXE7.8*) harbored two introns. The exon/intron analysis and the phylogenetic tree analysis results indicate that the distribution of *OsCXE* across different clades is random, and within the same phylogenetic clades, similar genes cluster together. Next, the motifs of *OsCXE* gene sequences were analyzed through online MEME, and a total of 10 motifs were identified (**Figure 4**). Clade I genes all contained nine motifs, namely motifs 6, 3, 1, 7, 2, 5, 8, 4, and 9. Motifs 6, 3, 1, 7, 2, 8, 5 and 4 were found in *OsCXE3.1* of clade II, while an additional motif 10 was found in *OsCXE8.3*. Most genes of clade III contained 10 motifs, which are motifs 1 to 10. Interestingly, *OsCXE9.1* lacked motif 10, while *OsCXE8.2* lacked motif 6. Most genes in clade IV contained nine motifs, namely motifs 6, 3, 1, 7, 2, 5, 8, 4 and 9, while *OsCXE7.8* was lack of motif 6, and *OsCXE7.9* and *OsCXE7.10* contained 10 motifs. Clades V and VII contained similar sequences with 10 motifs, namely motifs 1 to 10. In clade VI, *OsCXE3.2* had 10 motifs; *OsCXE5.1* and *OsCXE5.2* lacked motif 6; and *OsCXE11.1* lacked motifs 6 and 9. These motifs are distributed in the order of each subgroup, with similar motif arrangements within the same subgroup, indicating that the protein structure is conserved within specific subgroups, while the functions of most conserved motifs remain to be elucidated. Finally, Domain analysis showed that all *OsCXE* genes contained the Abhydrolase_3domain (**Figure 4**).

### Analysis of cis-regulatory elements in *OsCXE* promoters

**Figure 5:**
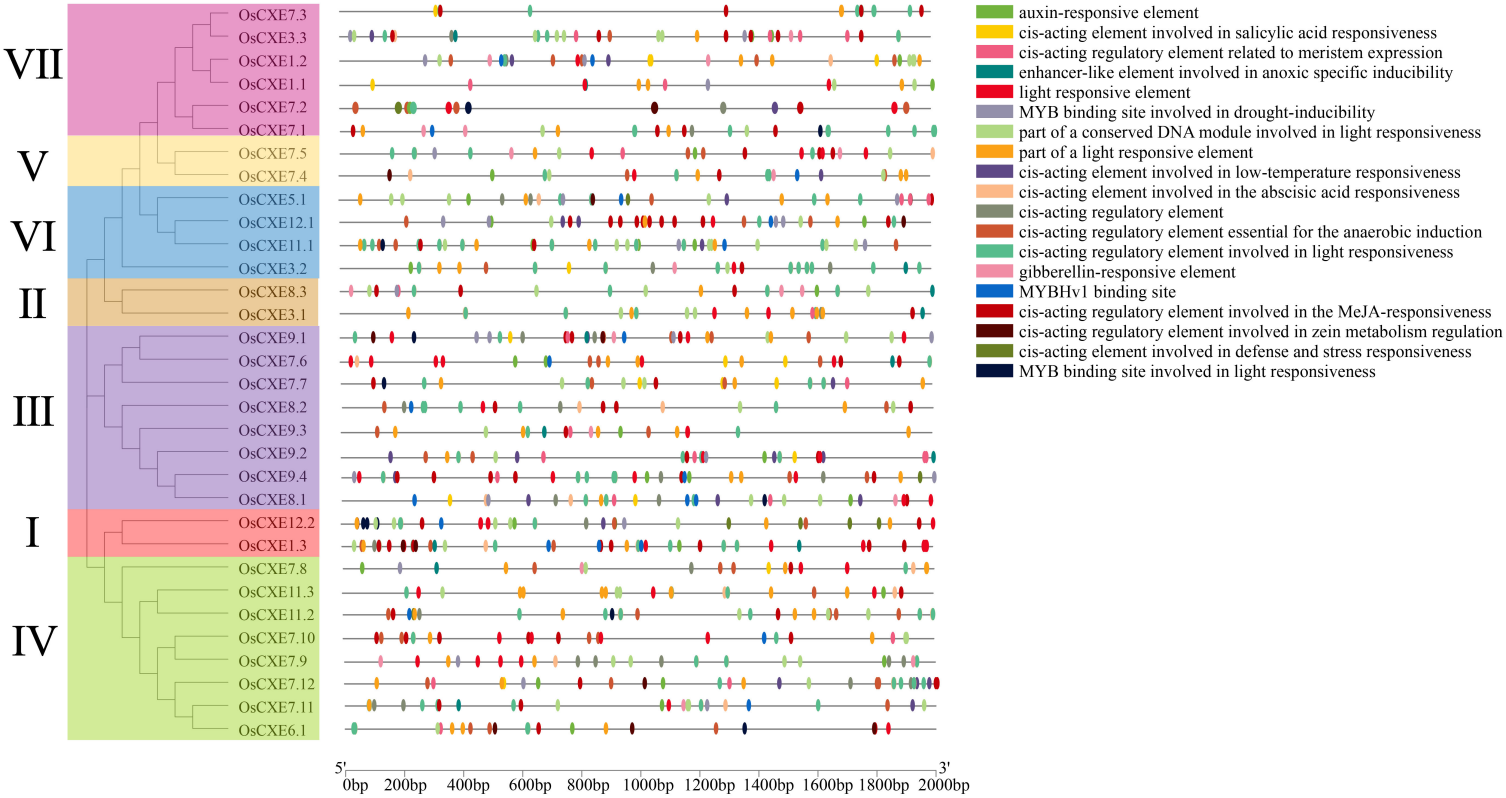
Detection of *cis*-regulatory elements in 32 *OsCXE* (Nipponbare) genes. *Cis*-elements with similar functions are shown in the same color. Black lines indicate the promoter length of *OsCXE* genes, and boxes of different colors represent *cis*-regulatory elements with different functions.

The distribution of *cis-*regulatory elements in response to stress and hormone is shown in **Figure 5**. *Cis*-regulatory elements in *OsCXE* are involved in a variety of abiotic stress responses, including MYB binding sites involved in drought induction, low temperature response, and anaerobic induction. *Cis*-regulatory elements of light response signaling and plant hormone synthesis are present in the promoter of each *OsCXE*, including ABA reaction, MeJA reaction, IAA response, and GA response. These results suggested that *OsCXE* play key roles in plant development and responses to abiotic stress and hormone.

### Three-dimensional structure prediction and interaction network analysis of *OsCXE* proteins

**Figure 6:**
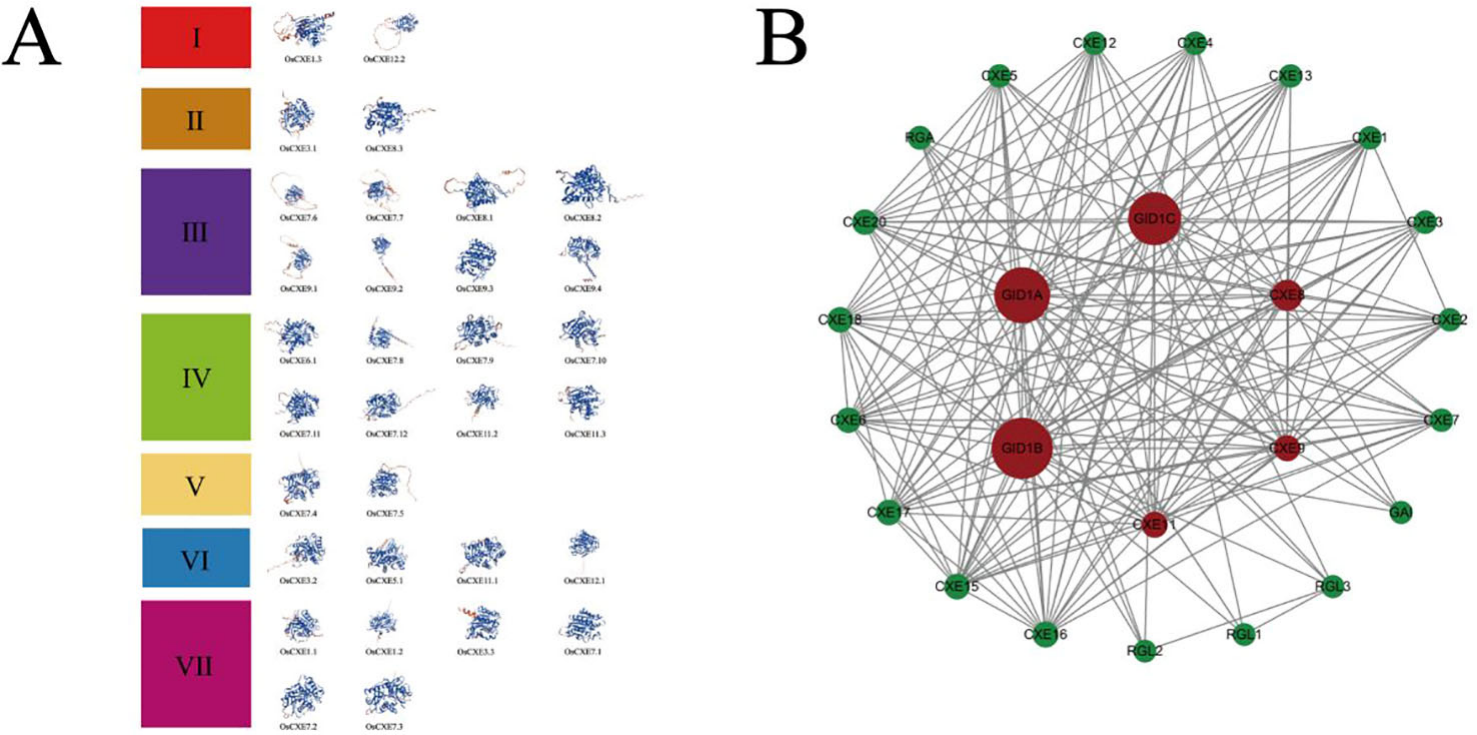
3D structure and network interactions of 32 *OsCXE* (Nipponbare) proteins. **(A)** Prediction of *OsCXE* protein tertiary structure. According to the phylogenetic relationship, these proteins were divided into seven clades, and the blue and red color of the three-dimensional protein structure represent low activity and high activity, respectively. **(B)** *OsCXE* protein interaction network. Red denotes the central gene, and lines indicate interactions.

The three-dimensional structure of a protein is mainly composed of α-helix, β-fold, and random helix, as shown in **Figure 6A**. These proteins were grouped according to their phylogenetic relationships. The prediction results showed that the 32 *OsCXE* proteins had different three-dimensional structures, indicating that they may have diverse functions. To further investigate the function of the *OsCXE* (Nipponbare) protein, we based on the homologous genes of *Arabidopsis thaliana*, conducted an interaction network analysis using an online platform(https://string-db.org/ (accessed on Sep 26, 2023), and utilized the Cytoscape software to beautify the network diagrams (**Figure 6B**). As shown in **Figure 6**, most of the promoters of *OsCXE* members had gibberellin-responsive elements, and the core gibberellin receptor genes *GID1A*, *GID1B*, and *GID1C* also demonstrated interactions wit*h OsCXE*. Moreover, *OsCXE* is also closely associated with the *RGL1*, *RGL2*, and *RGL3* genes in the interaction network map. By analyzing the cis-acting elements of the *OsCXE* proteins and their interactions within the protein network, we can gain a deeper understanding of the mechanisms of action of these proteins.

### Expression of *OsCXE* in different tissues and under abiotic stress

**Figure 7:**
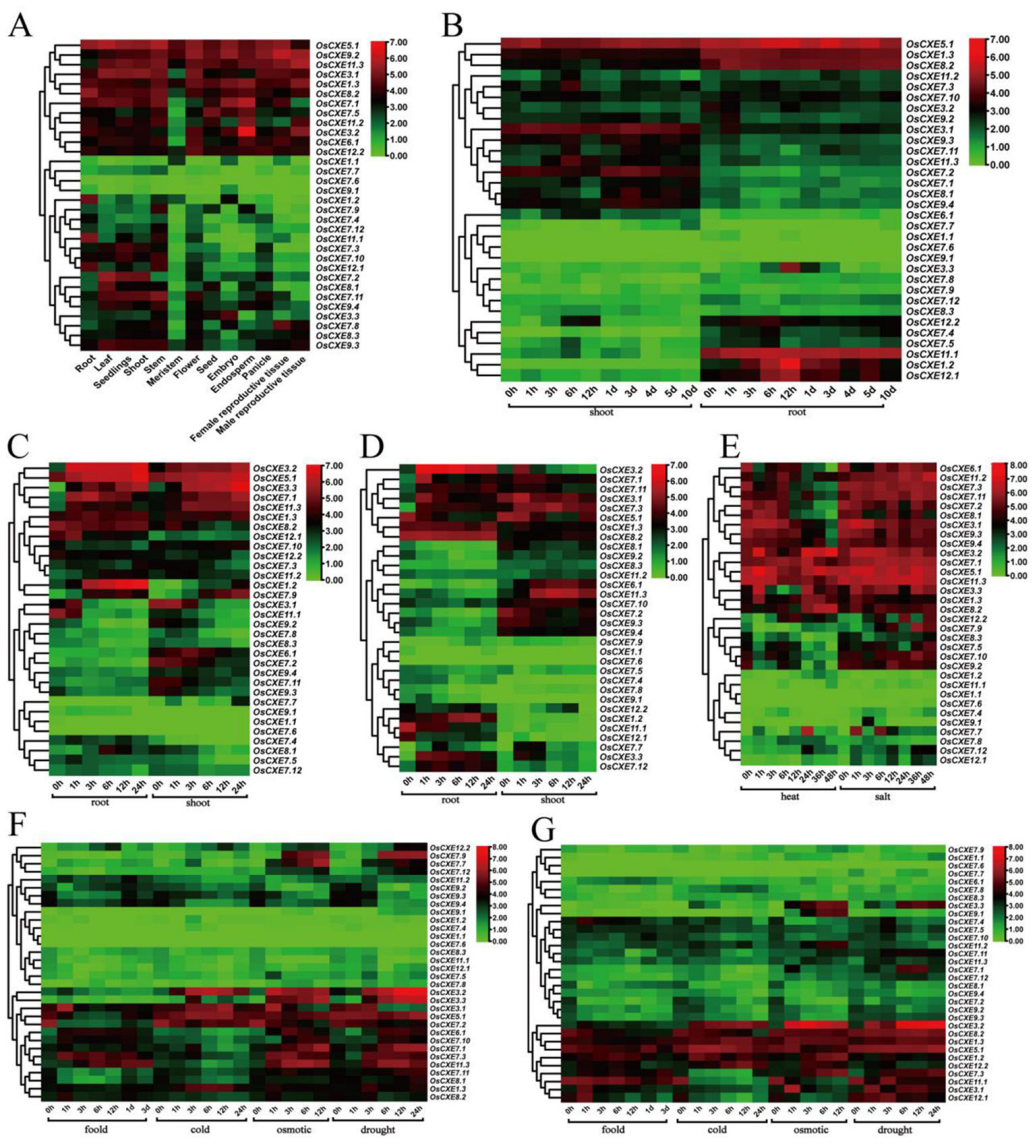
Analysis of 32 *OsCXE* (Nipponbare) gene expression. Color markers indicate changes in gene expression, with red indicating high expression and green indicating low expression. **(A)** Expression of *OsCXE* in the root, leaf, seedlings, shoot, stem, meristem, flower, seed, embryo, endosperm, panicle, female reproductive tissue, and male reproductive tissue under normal conditions. **(B)** Expression of *OsCXE* genes in shoots and roots at different developmental stages. **(C)** Expression levels of *OsCXE* genes in shoots and roots at different time after ABA treatment. **(D)** Expression levels of *OsCXE* genes in shoots and roots at different time after JA treatment. **(E)** Expression levels of *OsCXE* genes in stems and leaves under heat and salt stress at different time. **(F)** Expression levels of *OsCXE* genes at different time during flooding, cold, osmotic, and drought stress. **(G)** Expression of *OsCXE* genes at different time in roots under flooding, cold, osmotic and drought conditions.

To identify the potential function of *OsCXE* genes, we analyzed the transcription levels of *OsCXE* in various tissues of rice and under different stresses (**Figure 7**). The genes showed different levels of expression in different tissues, suggesting that they have different functions. Among them, *OsCXE1.1*, *OsCXE7.7*, *OsCXE7.6*, and *OsCXE9.1* had low expression levels in all tissues as shown in **Figure 7A**. However, *OsCXE5.1*, *OsCXE9.2*, *OsCXE11.3*, and *OsCXE1.3* were all highly expressed. *OsCXE3.2* was highly expressed in the endosperm. The expression of *OsCXE1.2* in the root was obviously up-regulated at 12h of development, and *OsCXE3.3* also had a certain increase in expression at 12h of development in root (**Figure 7B**). After ABA treatment, *OsCXE3.2*, *OsCXE5.1*, *OsCXE3.3*, *OsCXE7.1*, and *OsCXE11.3* were obviously up-regulated in the root and shoot, while *OsCXE1.2* was obviously up-regulated only in the root (**Figure 7C**). After ABA treatment, the expression of *OsCXE3.1* and *OsCXE11.1* decreased obviously in the root and shoot. After JA treatment, the expression of *OsCXE3.2* was obviously up-regulated in the root, while that of *OsCXE11.1* decreased. *OsCXE7.3* and *OsCXE11.3* showed increases in expression in the shoot after JA treatment (**Figure 7D**). The expression of *OsCXE7.7* increased in stems and leaves, while that of *OsCXE3.1*, *OsCXE7.2*, *OsCXE8.1*, *OsCXE9.3* and *OsCXE9.4* decreased after 12h of high-temperature treatment (**Figure 7E**). The expression of *OsCXE7.7* increased in stems and leaves after 1h and 6h of salt treatment (**Figure 7E**). *OsCXE3.1* expression in stems was obviously up-regulated after 1h of flooding treatment, and *OsCXE7.3* and *OsCXE11.3* also showed increases in expression afterl flooding stress (**Figure 7F**). The expression of *OsCXE3.1*, *OsCXE3.2*, *OsCXE5.1*, and *OsCXE1.3* in stems was increased after low-temperature stress (**Figure 7F**). *OsCXE3.2*, *OsCXE3.3*, *OsCXE7.1*, *OsCXE7.3* and *OsCXE11.3* in stems were up-regulated after osmotic stress. Interestingly, the expression of *OsCXE3.1* increased only after 1h of treatme(**Figure 7F**). *OsCXE3.2, OsCXE3.3, OsCXE7.1, OsCXE7.3, OsCXE7.9* and *OsCXE11.3* in stems showed increases in expression after drought stress. Interestingly, the expression of *OsCXE3.1* decreased obviously after 6h of treatment (**Figure 7F**). *OsCXE3.2* was obviously up-regulated in roots after low-temperature, osmotic, and drought stress (**Figure 7G**). *OsCXE* showed no obvious change in expression in shoots and roots after P stress, and in leaves after N and K treatment (**Supplementary Figure S2**).

### The expression of *OsCXE* in leaves under abiotic stress

**Figure 8:**
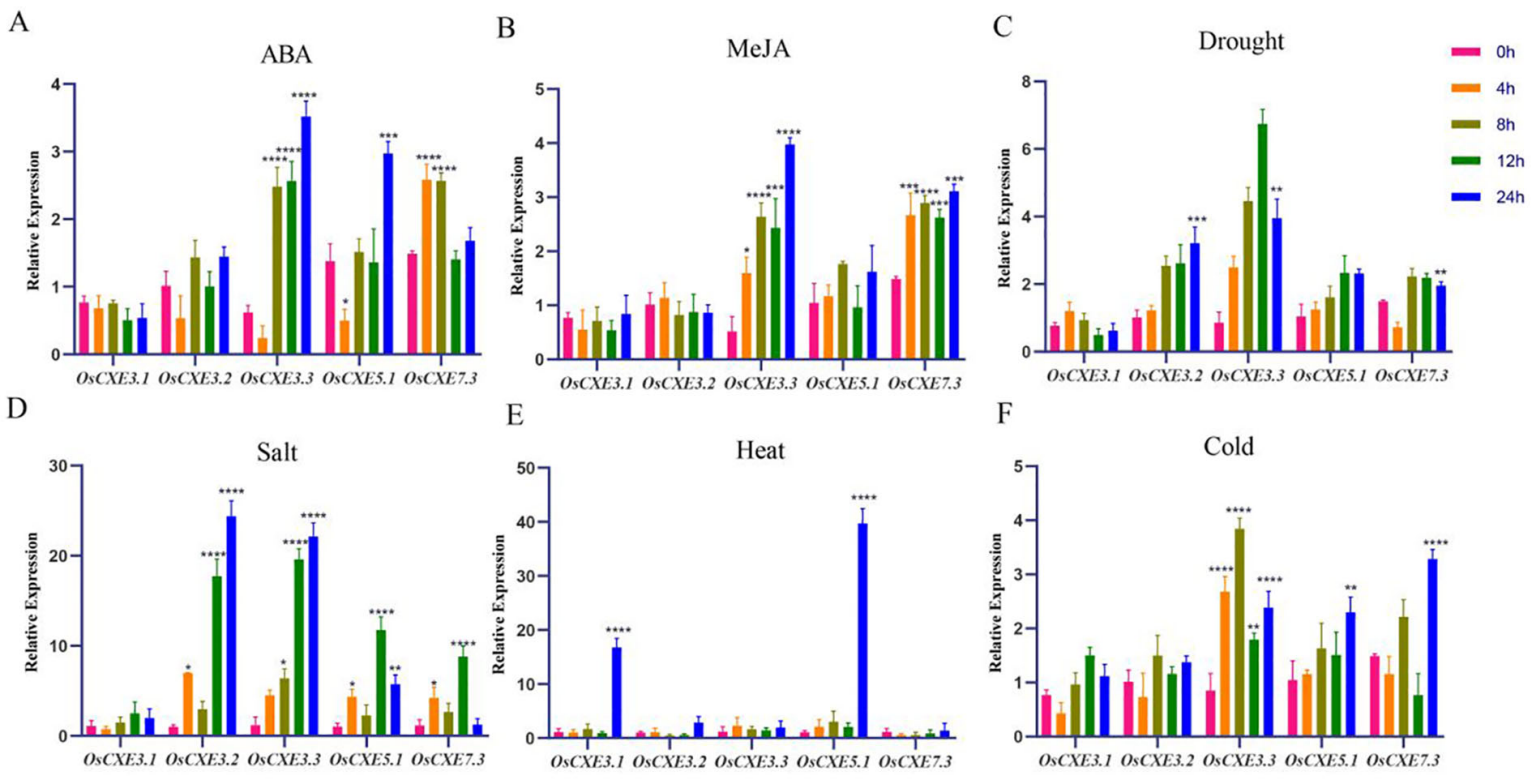
Analysis of expression levels of five genes of the *OsCXE* (Nipponbare) family under different treatments. **(A)** ABA treatment. **(B)** MeJA treatment. **(C)** Drought stress. **(D)** NaCl stress. **(E)** High temperature stress. **(F)** Low temperature stress. Statistical analysis of the data was performed using WPS2023 software, and IBM SPSS Statistics 25 statistics analysis software was used to perform analysis of variance; the significance level was defined as **** p < 0.0001, *** p < 0.001, ** p < 0.01, * p < 0.05.

Five genes (*OsCXE3.1, OsCXE3.2, OsCXE3.3, OsCXE7.3 and OsCXE11.3*) were selected as research objects based on *cis*-regulatory elements and RNA-seq analysis results, and their expression patterns under different treatments in leaves were analyzed (**Figure 8**). The expression levels of *OsCXE3.1* and *OsCXE3.2* showed no significant change under ABA hormone treatment, while those of *OsCXE3.3* and *OsCXE5.1* first decreased and then increased, and reached the peak at 24h. The expression of *OsCXE7.3* peaked at 4h and then decreased. After MeJA hormone treatment, *OsCXE3.3* and *OsCXE7.3* were significantly up-regulated, and both reached the highest expression at 24h. Under drought stress, the expression levels of both *OsCXE3.2* and *OsCXE3.3* were significantly up-regulated, but *OsCXE3.2* had a lower degree of up-regulation than *OsCXE3.3*, with the former reaching its peak at 24h while the latter reaching its maximum value at 12h. After NaCL stress treatment, the expression of all genes was extremely significantly up-regulated, except for O*sCXE3.1*, with the expression of *OsCXE3.2* and *OsCXE3.3* reaching the peak at 24 h, while that of *OsCXE5.1* and *OsCXE7.3* reaching the peak at 12h, among which *OsCXE3.2* was the most significantly up-regulated. After high-temperature treatment, only the expression of *OsCXE3.1* and *OsCXE5.1* showed explosive growth and reached the highest value in 24h. After low-temperature stress treatment, the expression of *OsCXE3.3* showed a general first increasing and then decreasing trend, reaching the peak at 12h. *OsCXE5.1* and *OsCXE7.3* showed an upward trend and both reached the peak in expression at 24h.

### Analysis of the diversity of *OsCXE* alleles in different populations

**Figure 9:**
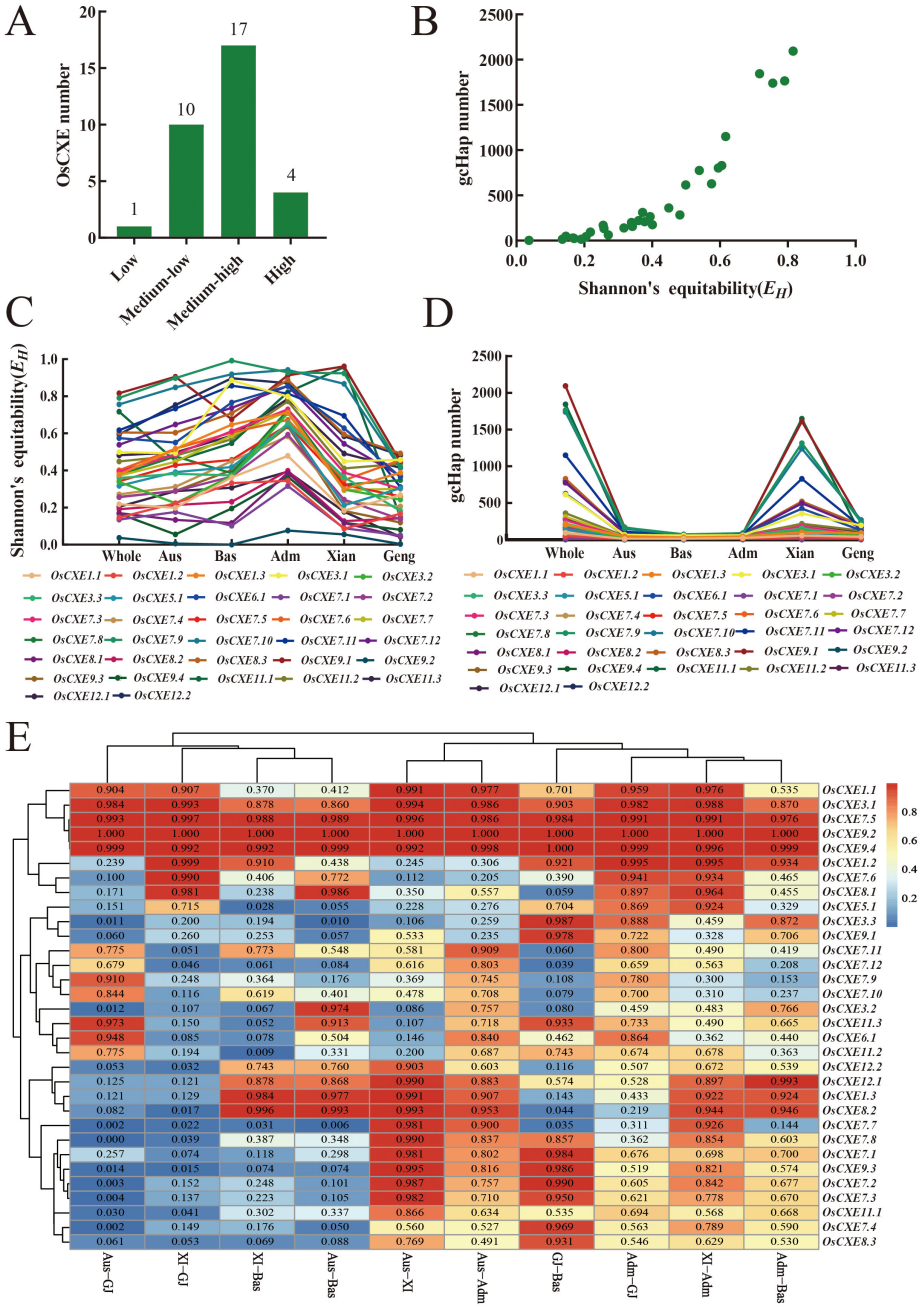
Population diversity of *OsCXE* genes in 3010 rice accessions. **(A)** Conserved genes (HK) (*E_H_* = 0 and gcHapN = 1), low diversity genes (0< *E_H_* < 0.05, $\bar{EH}$ = 0.020 ± 0.015, and gcHapN =12 ± 10), medium-low diversity genes (0.05 ≤ *E_H_* < 0.3, $\bar{EH}$ = 0.171 ± 0.065, and gcHapN = 82 ± 67), medium-high diversity gene (0.3≤*E_H_* <0.7, $\bar{EH}$ =0.444 ± 0.110,and gcHapN=498 ± 305), and high diversity gene (0.7≤*E_H_ ≤* 1, $\bar{EH}$ =0.804 ± 0.075,and gcHapN =1767 ± 458). **(B)** Relationship between Shannon fairness (*E_H_*) of 32 *OsCXE* gene and gcHapN. **(C)** The *E_H_* value of 32 *OsCXE* genes in different populations. **(D)** gcHap number (gcHapN) of *OsCXE* gene in different populations. **(E)** *I_Nei_* values of *OsCXE* genes between all paired populations calculated from gcHap data.

To understand the potential role of *OsCXE* alleles in the improvement of rice, we used CDS haplotype (gcHap) data from 3KRG. We obtained Shannon Fairness (*E_H_)*, gcHaps, and the number of major gcHaps (frequency ≥1% in 3KRG) for 32 *OsCXE* genes in four major rice populations (**Figure 9**; **Supplementary Table S3**). In 3KRG materials, there were one low-diversity *OsCXE* gene, ten medium-low diversity *OsCXE* genes, 17 medium-high diversity *OsCXE* genes, and four high-diversity *OsCXE* genes (**Figure 9A**). The average values of gcHaps (major gcHaps) and *E_H_* among the 32 polymorphic *OsCXE* genes in 3KRG materials were 482.3 (9.1) and 0.399, respectively. Different *OsCXE* svaried greatly in diversity, and the *E_H_* value generally increased with the number of gcHaps. *OsCXE9.1* had the highest diversity, with *E_H_* of 0.816 and 2094 gcHaps (**Figures 9C, D**), while *OsCXE9.2* had the lowest diversity, with *E_H_* of 0.037 and four gcHaps (**Supplementary Table S3**). The diversity of *OsCXE* salso varied greatly among different rice populations. The average *E_H_* of 32 *OsCXE* swas 0.393, 0.275, 0.435, 0.515, and 0.658 in *Xian*, *Geng*, Aus, Bas, and Adm population, respectively. The average number of gcHaps and major gcHaps was 341.6 and 8.3 in *Xian* population, 93 and 6.5 in *Geng* population, 50.3 and 7 in Aus population, and 27.6 and 6.3 in Bas population, respectively. There were 45.9 and 2.8 gcHaps and major gcHaps in the Adm population (**Supplementary Table S3**). It is clear that the number of gcHaps and major gcHaps has an important relationship with the size of the population, and most gcHaps are rare and low-frequency (**Figures 9C, D**).

To understand the differences in *OsCXE* sbetween different rice populations, we analyzed the gcHaps data of 32 polymorphic *OsCXE* in pairwise populations using genetic diversity data (*I_Nei_*) values. Among the 32 *OsCXE*, *OsCXE3.2*, *OsCXE7.1*, *OsCXE7.2, OsCXE7.4, OsCXE8.2, OsCXE9.3*, and *OsCXE12.1* showed strong XI-GJ differentiation (**Figure 9E**; **Supplementary Table S4**). In addition, the 32 *OsCXE* also showed strong differentiation in other populations, such as *O_S_CXE3.2* in Aus-XI, Aus-GJ, XI-Bas, and GJ-Bas (**Figure 9E**; **Supplementary Table S4**). These results indicated that allelic variation of *OsCXE* sites have contributed significantly to the differentiation of rice populations and the adaptation of different populations to the environment.

### Effects of modern breeding on gcHap diversity of *OsCXE*

In order to understand the effects of modern breeding on the gcHap diversity of *OsCXE* genes in rice in recent decades, we analyzed modern varieties (MVs) and local varieties (LANs), including 732 local varieties (LANs-*Xian*), 358 modern *Xian* varieties (MVs-*Xian*), 328 local verities of *Geng* (LANs-*Geng*), and 139 modern varieties of *Geng* (MVs-*Geng*) (**Supplementary Table S5**). Compared with LANs, MVs in *Xian* and *Geng* populations showed increases in average *E_H_*. Seven genes showed a significant increase in diversity in both *Xian* and *Geng* populations, while 12 genes showed a significant increase in their diversity only in *Xian*, and one gene exhibited significant increase in diversity only in *Geng* population (**Supplementary Table S5**). It was observed that MVs-*Xian* had an average of 91.9 gcHaps/locus, which is lower than that of LANs-*Xian* (159.8 gcHaps/locus), and MVs-*Geng* had an average of 28.1 gcHaps/locus, which is lower than that of LANs-*Geng* (49 gcHaps/locus). Further examination showed that MVs-*Xian* lost an average of 131.1 gcHaps/locus relative to LANs-*Xian* and MVs-*Geng* lost an average of 37.3 gcHaps/locus relative to LANs-*Geng*, which may be caused by the bottleneck effect of genetics.

With an average of 65.2 new gcHaps/locus obtained in MVs-*Xian*, which were not present in LANs-*Xian*, MVs-*Geng* obtained an average of 16.3 new gcHaps/locus, which were not present in *LANs-Geng*. The new gcHaps were apparently generated by intragenic recombination during breeding. We found deletions and new majority as rare gcHaps/locus. It is worth noting that the newly emerged gcHaps/locus in *Xian* population did not become the main gcHaps/locus in *Xian* population, but some newly emerged gcHaps/locus in *Geng* population have become major gcHaps/locus (**Supplementary Table S5**).

The 32 *OsCXE* in *Xian* population showed a significant increase in the frequency of major gcHaps F_(P)_ in three genes and a significant decrease in the frequency of the major gcHaps F_(P)_ in 12 genes. In the *Geng* population, the frequency of major gcHap F_(P)_ in only one gene was significantly increased, while that of five genes was significantly decreased. Further observations showed that most genes in the *OsCXE* family had the same major gcHaps in *Xian* and *Geng* populations, suggesting that most of the major gcHaps play a role in both *Xian* and *Geng* (**Supplementary Table S6**).

### Comparison of trait values between the predominant and ‘unfavorable’ gcHaps of *OsCXE* genes in rice

The dominant gcHap in CXE locus in rice populations, namely the gcHap with the highest frequency, is thought to have been favored by natural selection during evolution. Conversely, the major gcHap with the lowest frequency in the group is more likely to be an “unfavorable” gcHap (Zeng et al., 2023). We compared the phenotypic differences between the predominant and “unfavorable” gcHap on each of 32 CXE locus for 15 agronomic traits. Differences between the predominant and “unfavorable” gcHaps at CXE locus were detected in 196 (40.8%) cases out of a total of 480 comparisons (**Supplementary Table S3**-**S34**). In 25 out of the 32 *OsCXE*, phenotypic differences were observed between dominant gcHap and “unfavorable” gcHap in the trait of TGW. Among the 15 traits, *OsCXE7.6* showed the most differences, with phenotypic differences between the predominant gcHap and “unfavorable” gcHap for 10 traits. In addition, *OsCXE1.2, OsCXE3.2, OsCXE6.1, OsCXE7.4, OsCXE7.9, OsCXE8.1, OsCXE8.2*, and *OsCXE8.3* showed phenotypic differences between the predominant and “unfavorable” gcHap. More differences suggested that they may affect more traits (**Supplementary Table S7**).

### Correlation analysis of major gcHaps of *OsCXE* genes and important agronomic traits

**Figure 10:**
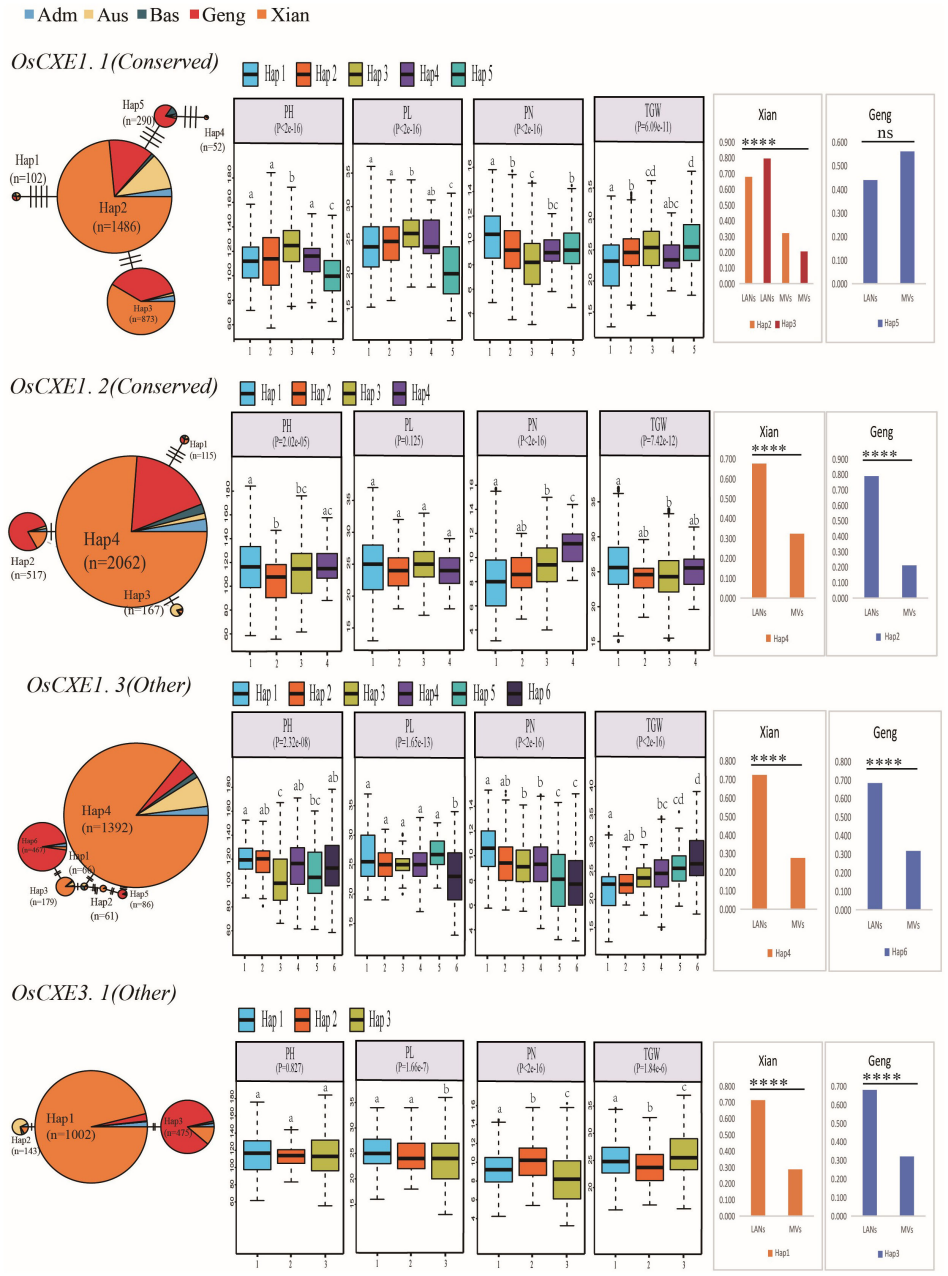
Haplotype network analysis of *OsCXE* genes and their associations with four agronomic traits in 3KRG.P-values under the trait names indicate differences between haplotypes assessed by a two-factor ANOVA, where different letters on the boxplot indicate statistically significant differences at P< 0.05 based on Duncan’s multiple range test. The bar chart on the right shows the difference in frequency of dominant gcHaps between local varieties (LANs) and modern varieties (MVs) of *Xian* and *Geng*. Chi-square tests were used to determine significant differences in the proportion of the same gcHap between different populations ****P<0.0001, ***P<0.001, **P<0.01, *P<0.05 and N.S., not significant.

To demonstrate the functional importance of *OsCXE* genes, we constructed a gcHaps network for the dominant alleles of 32 genes in five rice populations, and analyzed the dominant alleles of 32 *OsCXE* genes in 3KRG and four agronomic traits, including panicle number per plant (PN), panicle length (PL), plant height (PH), thousand grain weight (TGW) (**Figure 10**; **Supplementary Figures S35**–**S41**). Strong (P< 10^-7^) associations were observed in 64 (50%) of 128 (32 × 4) cases, and the major alleles of multiple *OsCXE* genes were strongly associated with the values of one or more traits. Further analysis of *OsCXE* genes showed that *OsCXE1.1* is a conserved gene with five major gcHaps. There were three non-synonymous mutations between Hap2 and Hap5, which significantly increased PN during breeding compared with Hap2. *OsCXE1.2* is a conserved gene with four major gcHaps. Hap4 has a high frequency in LANs-*Xian* and differs from Hap2 in two non-synonymous mutations that have become dominant gcHaps in LANs-*Geng* and significantly increase TGW during breeding. *OsCXE1.3* is an “other” gene, and there are six major gcHaps. Hap4 and Hap6 have a high frequency in LANs-*Xian* and LANs-*Geng*, respectively. Hap6 has significantly increased PL and PN during breeding compared with Hap4. *OsCXE3.1* is also an “other” gene, and Hap1 has a high frequency in LANs-*Xian*. Hap3 differs from Hap1 in two non-synonymous mutations that have become dominant gcHaps of LANs-*Geng* and significantly increase TGW during breeding. Among the 32 *OsCXE* sgenes, *OsCXE1.1, OsCXE5.1*, and *OsCXE7.7* were closely related to the values of the four traits.

### Mining of “favorable” alleles at *OsCXE* locus to increase yield

**Figure 11:**
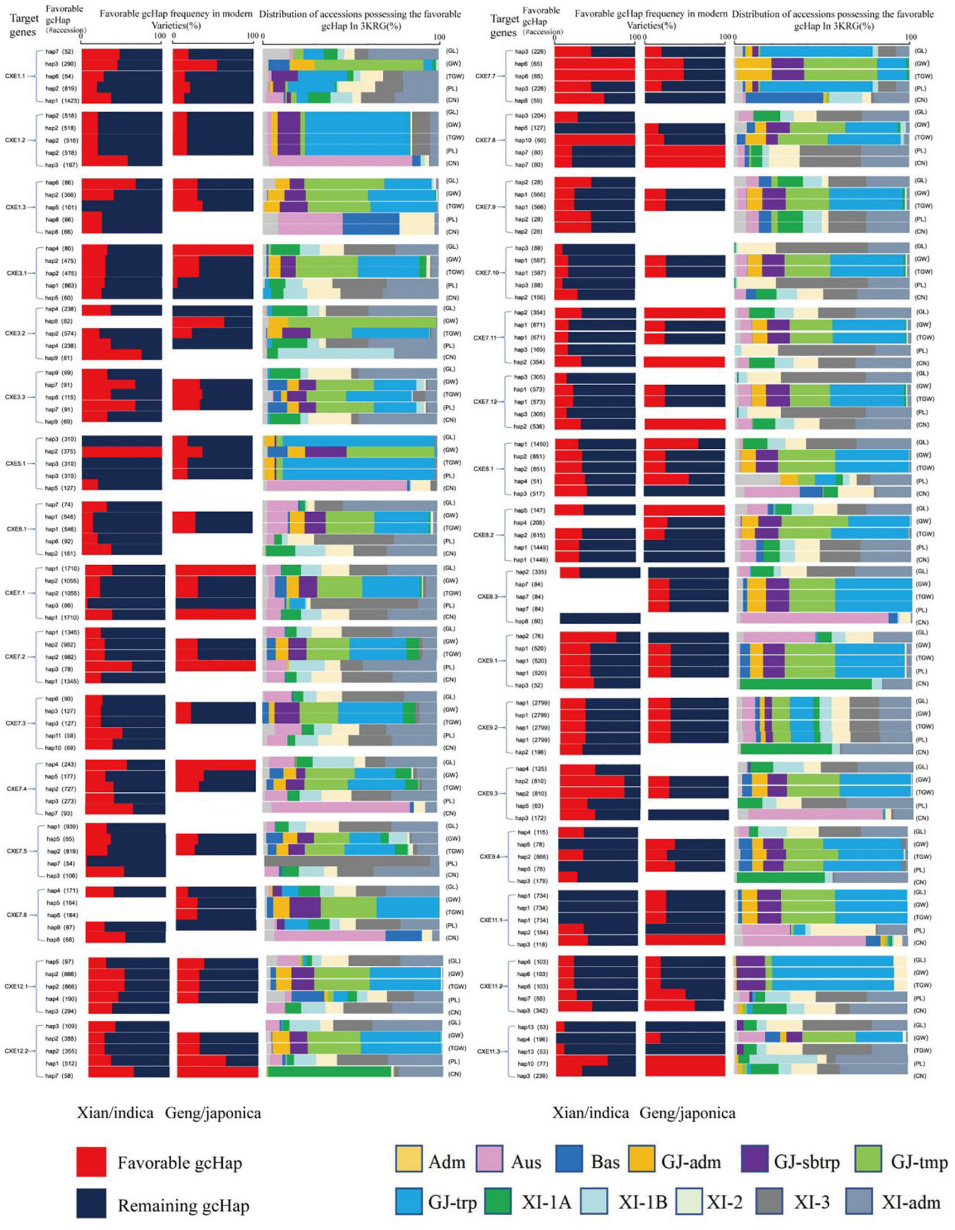
Favorable gcHap frequencies of 32 *OsCXE* genes affecting TGW, GL, GW, PL and CN in *Xian/Indica* rice (XI), *Geng*/Japonica rice (GJ) and different rice subpopulations.The “favorable” gcHaps of a gene are defined as the gcHAPs associated with the highest trait values. “#accession” indicates the number of accessions with “favorable” gcHaps. Distribution frequency of five subpopulations of *XI* (*XI-1A*, *XI-1B*, *XI-2*, *XI-3*, and *XI-ADM*) and four subpopulations of GJ (temperate GJ [GJ-TMP], subtropical GJ [GJ-SBTRP], tropical GJ [GJ-TRP], and GJ-ADM).

The effects of major gcHaps at *OsCXE* locus on important agronomic traits of rice were significantly different. The “favorable” allele frequency was different for different yield traits, CXE locus, yield traits, rice populations, and favorable allele frequencies. For example, Hap4 at *OsCXE3.1* was the most associated with the GL trait in 3KRG germplasm, and its frequency was fixed in MVs-*Geng* but less frequent in MVs-*Xian*. In addition, Hap1 at *OsCXE7.1* had the highest correlation with GL and CN. Hap3 at *OsCXE7.2* was the most correlated with PL. Hap7 at *OsCXE7.8* had the highest correlation with PL and CN. Hap2 at *OsCXE7.11* showed the highest correlation with GL and CN. Hap2 at *OsCXE7.12* had the highest correlation with CN. Hap5 at *OsCXE8.2* showed the highest correlation with GL. Hap3 at *OsCXE11.1* had the highest correlation with CN. Hap10 at *OsCXE11.3* had the highest correlation with PL and Hap13 with CN. Hap7 at *OsCXE12.2* has the highest correlation with CN, and their frequency is fixed at MVs-*Geng*. Among the genes with fixed frequency in MVs-*Xian*, *OsCXE7.2* and Hap3 had the highest correlation with PL. Hap6 at *OsCXE7.7* was the most correlated with TGW and GW. Hap10 at *OsCXE7.8* had the highest correlation with TGW (**Figure 11**). These results suggested that the “favorable” alleles for different yield traits may vary significantly between the two rice subspecies or genetic contexts, and possibly in different environments.

## Discussion

CXE is a hydrolase that can catalyze ester and amide compounds, playing important roles in plant growth and development, disease resistance and stress response (Li et al., 2024; Wang et al., 2024). It is a large gene family in the rice genome, but the molecular and functional characteristics of most *OsCXE* genes remain largely unknown. In this study, we performed a whole-genome identification of the CXE gene family in rice through bioinformatics analysis, gene haplotype diversity analysis, and some other methods, and selected some non-biological genes with potential functions through structural analysis and transcriptome analysis. We also studied the expression patterns of these genes in response to environmental stress. A diversity analysis was performed in 3010 rice samples of gcHap, which provide important insights into the function and evolutionary history of *OsCXE* genes.

Firstly, most *OsCXE* proteins are unstable and hydrophilic according to the analysis of physical and chemical properties (**Supplementary Table S2**). Subcellular localization prediction showed that most of *OsCXE* genes are located on the cell membrane and chloroplast (**Supplementary Table S2**), this provides clues as to their biological functions.Gene structure analysis showed that *OsCXE* genes have a very similar exon-intron distribution pattern and a conserved motif distribution. Some clades have specific structure pattern, and the structure pattern is similar within the same group (**Figure 4**). The collinearity analysis (**Figure 2**; **Supplementary Figure S1**) and evolutionary selection pressure analysis (**Figure 3**) showed that the *OsCXE* family has experienced strong purification selection pressure during evolution,fragment duplication plays a key role in family amplification.In order to understand the evolutionary relationship between indica and japonica rice, four rice species (two japonica rice and two indica rice) were subjected to pairwise comparison, and collinearity analysis result (**Figure 2**) between them supported the close evolutionary relationship between japonica and indica rice (Garris et al., 2005).

Secondly, the results of the cis-acting element analysis (**Figure 5**) indicate that most promoters of the *OsCXE* family members contain gibberellin-responsive elements, which is consistent with the results of protein interaction analysis based on the homologous genes of *Arabidopsis thaliania* CXE (**Figure 6**). Previous studies have shown that *GID*1 in rice is involved in GA signal transduction by binding to GA and forming a *GID1-GA-SLR1* complex, thereby affecting the expression of various proteins and genes, and is related to the stress resistance of rice (Ueguchi-Tanaka et al., 2005, 2007; Yamamoto et al., 2010). Therefore, it is speculated that the *OsCXE* family members may be closely related to *GID1*. Transcriptome data analysis (**Figure 7**) and real-time fluorescence quantitative analysis (**Figure 8**) show that the *OsCXE* genes exhibit different expression patterns in different tissues and stress conditions of rice, suggesting their specific functions in plant physiology.

Thirdly,by analyzing the CDS haplotype data of 3 KRG,we found that the *OsCXE* gene may play an important role in the evolution and population differentiation of rice, with most *OsCXE* loci showing strong differentiation across different populations. Additionally, in the 3KRG materials, the genetic diversity of the *OsCXE* gene family exhibits significant differences among various rice populations (**Figure 9**; **Supplementary Tables S3**–**S4**). The study also revealed that modern breeding has had a significant impact on the genetic diversity of these genes, which is consistent with the results of Zeng et al.’s research (Zeng et al., 2023) For example, in the *Xian* population, modern varieties (MVs-*Xian*) compared to local varieties (LANs-*Xian*), the average number of gcHaps decreased from 159.8 to 91.9, indicating a possible genetic bottleneck effect. Furthermore, modern breeding has also led to the emergence of some new gcHaps, such as MVs-*Xian*, which on average acquired 65.2 new gcHaps (**Supplementary Table S5**).

Finally, further association analysis has revealed a significant link between the *OsCXE* gene and important agronomic traits (**Figure 10**; **Supplementary Figures S35**–**S41**), suggesting that the *OsCXE* gene may play an important role in rice improvement. For instance, two main gcHaps (Hap2 and Hap5) of the *OsCXE1.1* gene are significantly correlated with the increase in TGW (**Figure 10**). The *OsCXE7.6* gene shows phenotypic differences between dominant and “unfavorable” gcHaps across 10 distinct traits(**Supplementary Table S7**), Suggesting that it may play a key role in regulating rice traits.Besides,*OsCXE1.2*, *OsCXE3.2, OsCXE6.1, OsCXE7.4, OsCXE7.9, OsCXE8.1, OsCXE8.2*, and *OsCXE8.3* had phenotypic differences between dominant and “unfavorable” gcHaps. More differences indicated that they may affect many more traits (**Figure 11**). The existence of such differences may imply that natural selection has favored specific alleles during the evolutionary process, which may have a positive effect on improving rice yield and agronomic traits, necessitating further research. The study also found that the frequency distribution of some advantageous gcHaps at the *OsCXE* gene loci varies among different rice populations (**Figure 11**), which may reflect genetic variations adapted to different ecological environments (Zhang et al., 2021; Zeng et al., 2023). These findings provide different perspectives for the precise improvement of rice traits, while also indicating that the specific needs of different populations and the maintenance of genetic diversity should be considered during the breeding process.

## Data Availability

The datasets presented in this study can be found in online repositories. The names of the repository/repositories and accession number(s) can be found in the article/**Supplementary Material**.

## Supplementary material

Supplementary Figure 1 Collinear relationship of CXE genes in rice.

Supplementary Figure 2 Analysis of 32 *OsCXE* (Nipponbare) gene expression. Color markers indicate changes in gene expression, red indicates high expression and green low expression. **(A)** Expression of *OsCXE* in root and aerial parts at different times under P stress. **(B)** Expression of *OsCXE* in leaves at different times under N and K treatment.

Supplementary Figure 3 Comparison and analysis of 15 agronomic traits among the predominant gcHap, unfavorable gcHap, and major gcHaps of *OsCXE1.1*.

Supplementary Figure 4 Comparison and analysis of 15 agronomic traits among the predominant gcHap, unfavorable gcHap, and major gcHaps of *OsCXE1.2*.

Supplementary Figure 5 Comparison and analysis of 15 agronomic traits among the predominant gcHap, unfavorable gcHap, and major gcHaps of *OsCXE1.3*.

Supplementary Figure 6 Comparison and analysis of 15 agronomic traits among the predominant gcHap, unfavorable gcHap, and major gcHaps of *OsCXE3.1*.

Supplementary Figure 7 Comparison and analysis of 15 agronomic traits among the predominant gcHap, unfavorable gcHap, and major gcHaps of *OsCXE3.2*.

Supplementary Figure 8 Comparison and analysis of 15 agronomic traits among the predominant gcHap, unfavorable gcHap, and major gcHaps of *OsCXE3.3*.

Supplementary Figure 9 Comparison and analysis of 15 agronomic traits among the predominant gcHap, unfavorable gcHap, and major gcHaps of *OsCXE5.1*.

Supplementary Figure 10 Comparison and analysis of 15 agronomic traits among the predominant gcHap, unfavorable gcHap, and major gcHaps of *OsCXE6.1*.

Supplementary Figure 11 Comparison and analysis of 15 agronomic traits among the predominant gcHap, unfavorable gcHap, and major gcHaps of *OsCXE7.1*.

Supplementary Figure 12 Comparison and analysis of 15 agronomic traits among the predominant gcHap, unfavorable gcHap, and major gcHaps of *OsCXE7.2*.

Supplementary Figure 13 Comparison and analysis of 15 agronomic traits among the predominant gcHap, unfavorable gcHap, and major gcHaps of *OsCXE7.3*.

Supplementary Figure 14 Comparison and analysis of 15 agronomic traits among the predominant gcHap, unfavorable gcHap, and major gcHaps of *OsCXE7.4*.

Supplementary Figure 15 Comparison and analysis of 15 agronomic traits among the predominant gcHap, unfavorable gcHap, and major gcHaps of *OsCXE7.5*.

Supplementary Figure 16 Comparison and analysis of 15 agronomic traits among the predominant gcHap, unfavorable gcHap, and major gcHaps of *OsCXE7.6*.

Supplementary Figure 17 Comparison and analysis of 15 agronomic traits among the predominant gcHap, unfavorable gcHap, and major gcHaps of *OsCXE7.7*.

Supplementary Figure 18 Comparison and analysis of 15 agronomic traits among the predominant gcHap, unfavorable gcHap, and major gcHaps of *OsCXE7.8*.

Supplementary Figure 19 Comparison and analysis of 15 agronomic traits among the predominant gcHap, unfavorable gcHap, and major gcHaps of *OsCXE7.9*.

Supplementary Figure 20 Comparison and analysis of 15 agronomic traits among the predominant gcHap, unfavorable gcHap, and major gcHaps of *OsCXE7.10*.

Supplementary Figure 21 Comparison and analysis of 15 agronomic traits among the predominant gcHap, unfavorable gcHap, and major gcHaps of *OsCXE7.11*.

Supplementary Figure 22 Comparison and analysis of 15 agronomic traits among the predominant gcHap, unfavorable gcHap, and major gcHaps of *OsCXE7.12*.

Supplementary Figure 23 Comparison and analysis of 15 agronomic traits among the predominant gcHap, unfavorable gcHap, and major gcHaps of *OsCXE8.1*.

Supplementary Figure 24 Comparison and analysis of 15 agronomic traits among the predominant gcHap, unfavorable gcHap, and major gcHaps of *OsCXE8.2*.

Supplementary Figure 25 Comparison and analysis of 15 agronomic traits among the predominant gcHap, unfavorable gcHap, and major gcHaps of *OsCXE8.3*.

Supplementary Figure 26 Comparison and analysis of 15 agronomic traits among the predominant gcHap, unfavorable gcHap, and major gcHaps of *OsCXE9.1*.

Supplementary Figure 27 Comparison and analysis of 15 agronomic traits among the predominant gcHap, unfavorable gcHap, and major gcHaps of *OsCXE9.2*.

Supplementary Figure 28 Comparison and analysis of 15 agronomic traits among the predominant gcHap, unfavorable gcHap, and major gcHaps of *OsCXE9.3*.

Supplementary Figure 29 Comparison and analysis of 15 agronomic traits among the predominant gcHap, unfavorable gcHap, and major gcHaps of *OsCXE9.4*.

Supplementary Figure 30 Comparison and analysis of 15 agronomic traits among the predominant gcHap, unfavorable gcHap, and major gcHaps of *OsCXE11.1*.

Supplementary Figure 31 Comparison and analysis of 15 agronomic traits among the predominant gcHap, unfavorable gcHap, and major gcHaps of *OsCXE11.2*.

Supplementary Figure 32 Comparison and analysis of 15 agronomic traits among the predominant gcHap, unfavorable gcHap, and major gcHaps of *OsCXE11.3*.

Supplementary Figure 33 Comparison and analysis of 15 agronomic traits among the predominant gcHap, unfavorable gcHap, and major gcHaps of *OsCXE12.1*.

Supplementary Figure 34 Comparison and analysis of 15 agronomic traits among the predominant gcHap, unfavorable gcHap, and major gcHaps of *OsCXE12.2*.

Supplementary Figure 35 Haplotype network analysis of *OsCXE* genes and their associations with four agronomic traits in 3KRG.P-values under the trait names indicate differences between haplotypes assessed by a two-factor ANOVA, where different letters on the boxplot indicate statistically significant differences at P< 0.05 based on Duncan’s multiple range test. The bar chart on the right shows the difference in frequency of dominant gcHaps between local varieties (LANs) and modern varieties (MVs) of *Xian* and *Geng*. Chi-square tests were used to determine significant differences in the proportion of the same gcHap between different populations ****P<0.0001, ***P<0.001, **P<0.01, *P<0.05 and N.S.,not significant.

Supplementary Figure 36 Haplotype network analysis of *OsCXE* genes and their associations with four agronomic traits in 3KRG.P-values under the trait names indicate differences between haplotypes assessed by a two-factor ANOVA, where different letters on the boxplot indicate statistically significant differences at P< 0.05 based on Duncan’s multiple range test. The bar chart on the right shows the difference in frequency of dominant gcHaps between local varieties (LANs) and modern varieties (MVs) of *Xian* and *Geng*. Chi-square tests were used to determine significant differences in the proportion of the same gcHap between different populations ****P<0.0001, ***P<0.001, **P<0.01, *P<0.05 and N.S.,not significant.

Supplementary Figure 37 Haplotype network analysis of *OsCXE* genes and their associations with four agronomic traits in 3KRG.P-values under the trait names indicate differences between haplotypes assessed by a two-factor ANOVA, where different letters on the boxplot indicate statistically significant differences at P< 0.05 based on Duncan’s multiple range test. The bar chart on the right shows the difference in frequency of dominant gcHaps between local varieties (LANs) and modern varieties (MVs) of *Xian* and *Geng*. Chi-square tests were used to determine significant differences in the proportion of the same gcHap between different populations ****P<0.0001, ***P<0.001, **P<0.01, *P<0.05 and N.S.,not significant.

Supplementary Figure 38 Haplotype network analysis of *OsCXE* genes and their associations with four agronomic traits in 3KRG.P-values under the trait names indicate differences between haplotypes assessed by a two-factor ANOVA, where different letters on the boxplot indicate statistically significant differences at P< 0.05 based on Duncan’s multiple range test. The bar chart on the right shows the difference in frequency of dominant gcHaps between local varieties (LANs) and modern varieties (MVs) of *Xian* and *Geng*. Chi-square tests were used to determine significant differences in the proportion of the same gcHap between different populations ****P<0.0001, ***P<0.001, **P<0.01, *P<0.05 and N.S.,not significant.

Supplementary Figure 39 Haplotype network analysis of *OsCXE* genes and their associations with four agronomic traits in 3KRG.P-values under the trait names indicate differences between haplotypes assessed by a two-factor ANOVA, where different letters on the boxplot indicate statistically significant differences at P< 0.05 based on Duncan’s multiple range test. The bar chart on the right shows the difference in frequency of dominant gcHaps between local varieties (LANs) and modern varieties (MVs) of *Xian* and *Geng*. Chi-square tests were used to determine significant differences in the proportion of the same gcHap between different populations ****P<0.0001, ***P<0.001, **P<0.01, *P<0.05 and N.S.,not significant.

Supplementary Figure 40 Haplotype network analysis of *OsCXE* genes and their associations with four agronomic traits in 3KRG.P-values under the trait names indicate differences between haplotypes assessed by a two-factor ANOVA, where different letters on the boxplot indicate statistically significant differences at P< 0.05 based on Duncan’s multiple range test. The bar chart on the right shows the difference in frequency of dominant gcHaps between local varieties (LANs) and modern varieties (MVs) of *Xian* and *Geng*. Chi-square tests were used to determine significant differences in the proportion of the same gcHap between different populations ****P<0.0001, ***P<0.001, **P<0.01, *P<0.05 and N.S.,not significant.

Supplementary Figure 41 Haplotype network analysis of *OsCXE* genes and their associations with four agronomic traits in 3KRG.P-values under the trait names indicate differences between haplotypes assessed by a two-factor ANOVA, where different letters on the boxplot indicate statistically significant differences at P< 0.05 based on Duncan’s multiple range test. The bar chart on the right shows the difference in frequency of dominant gcHaps between local varieties (LANs) and modern varieties (MVs) of *Xian* and *Geng*. Chi-square tests were used to determine significant differences in the proportion of the same gcHap between different populations ****P<0.0001, ***P<0.001, **P<0.01, *P<0.05 and N.S.,not significant.
