# Supplementary figures and images for: Genome-wide identification of rice CXE gene family and mining of alleles for potential application in rice improvement
Source: Front Plant Sci. 2024 Oct 17;15:1435420. doi: 10.3389/fpls.2024.1435420 (PMC11524881; doi:10.3389/fpls.2024.1435420)

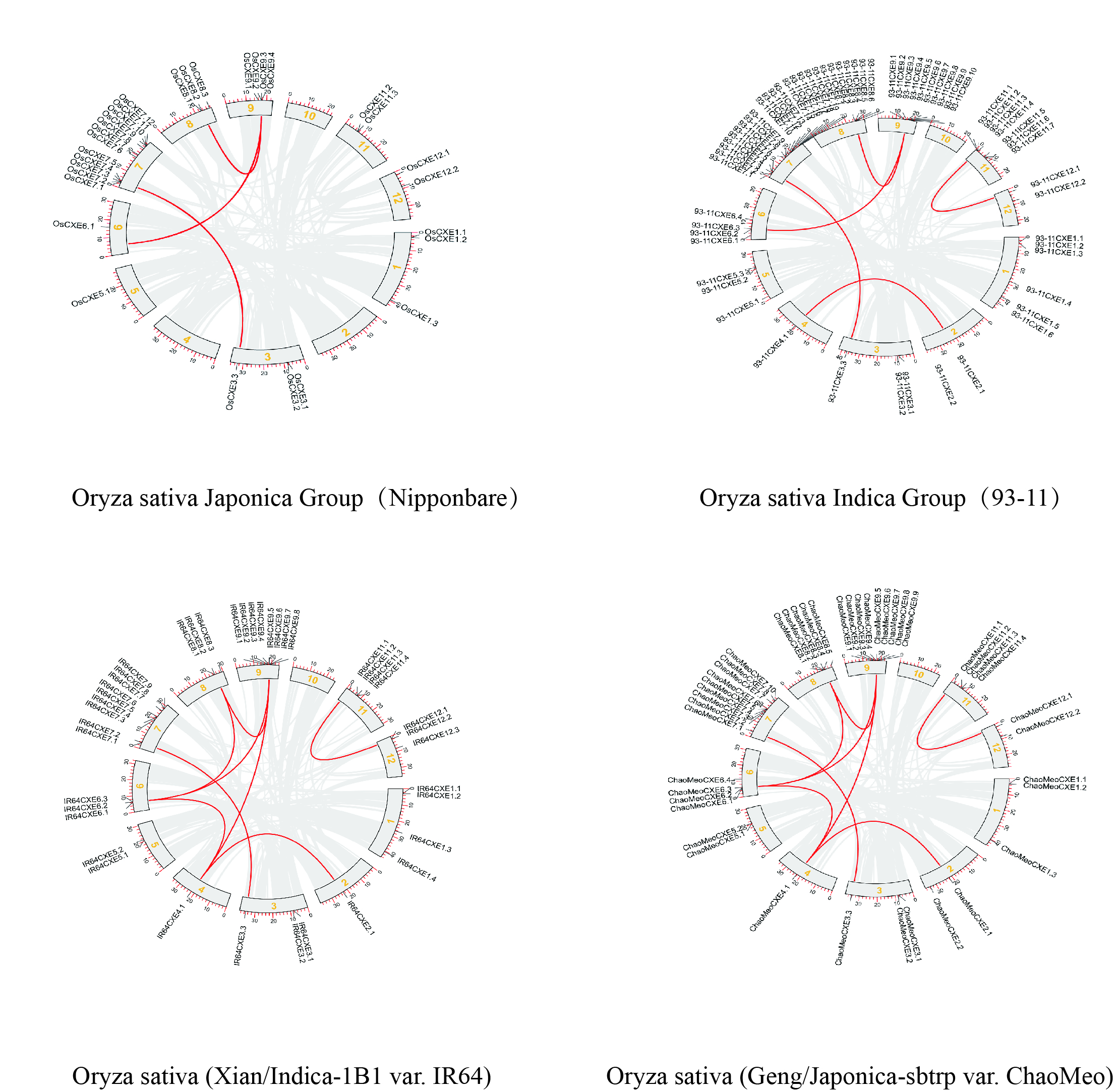

Supplement: Supplementary Figure 1 — Collinear relationship of CXE genes in rice. [file DataSheet3.zip › Supplementary Figure 1-15/Supplementary Figure 1 Collinear relationship of CXE genes in rice.jpg]

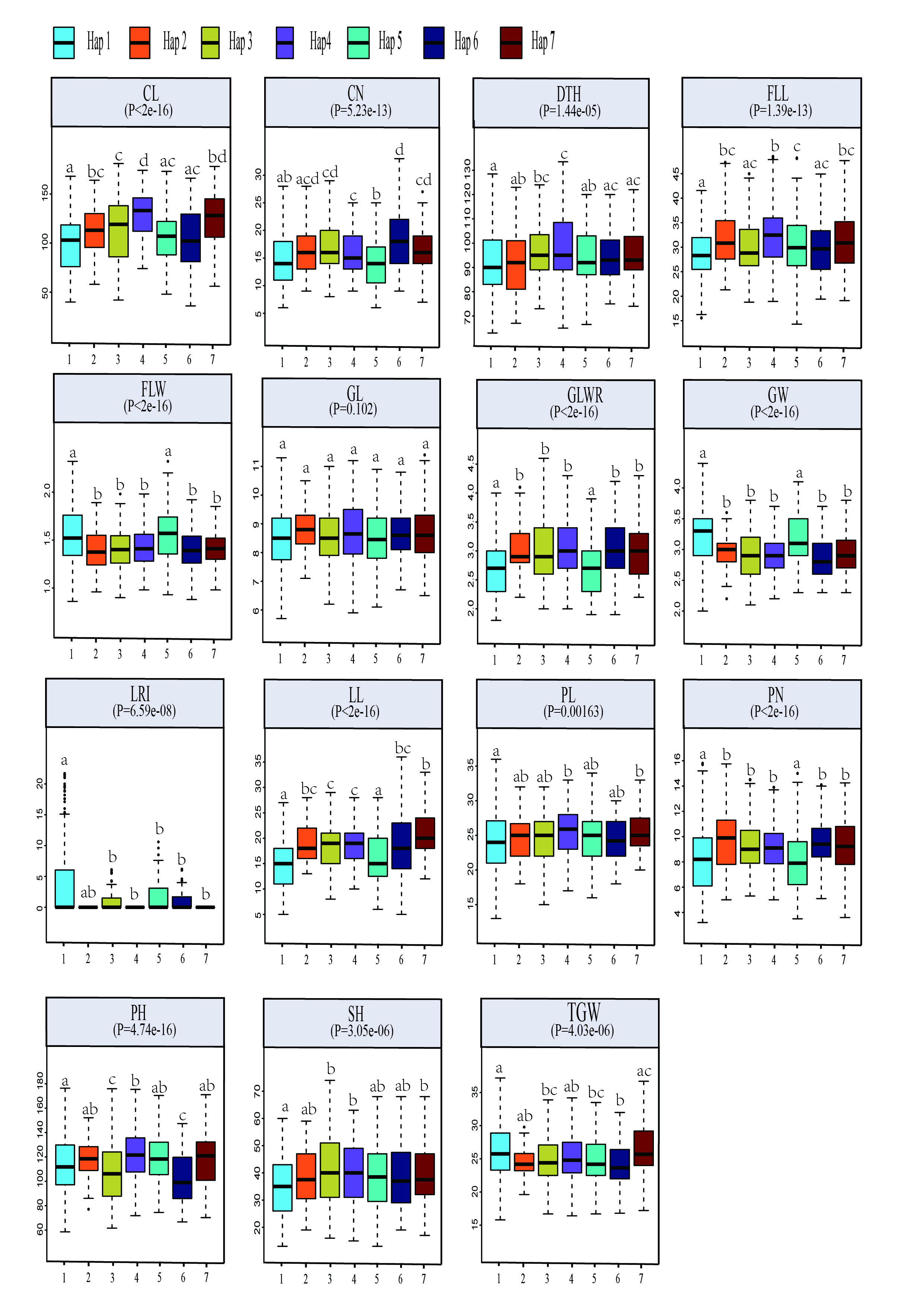

Supplement: Supplementary Figure 1 — Collinear relationship of CXE genes in rice. [file DataSheet3.zip › Supplementary Figure 1-15/Supplementary Figure 10 cxe6.1.jpg]

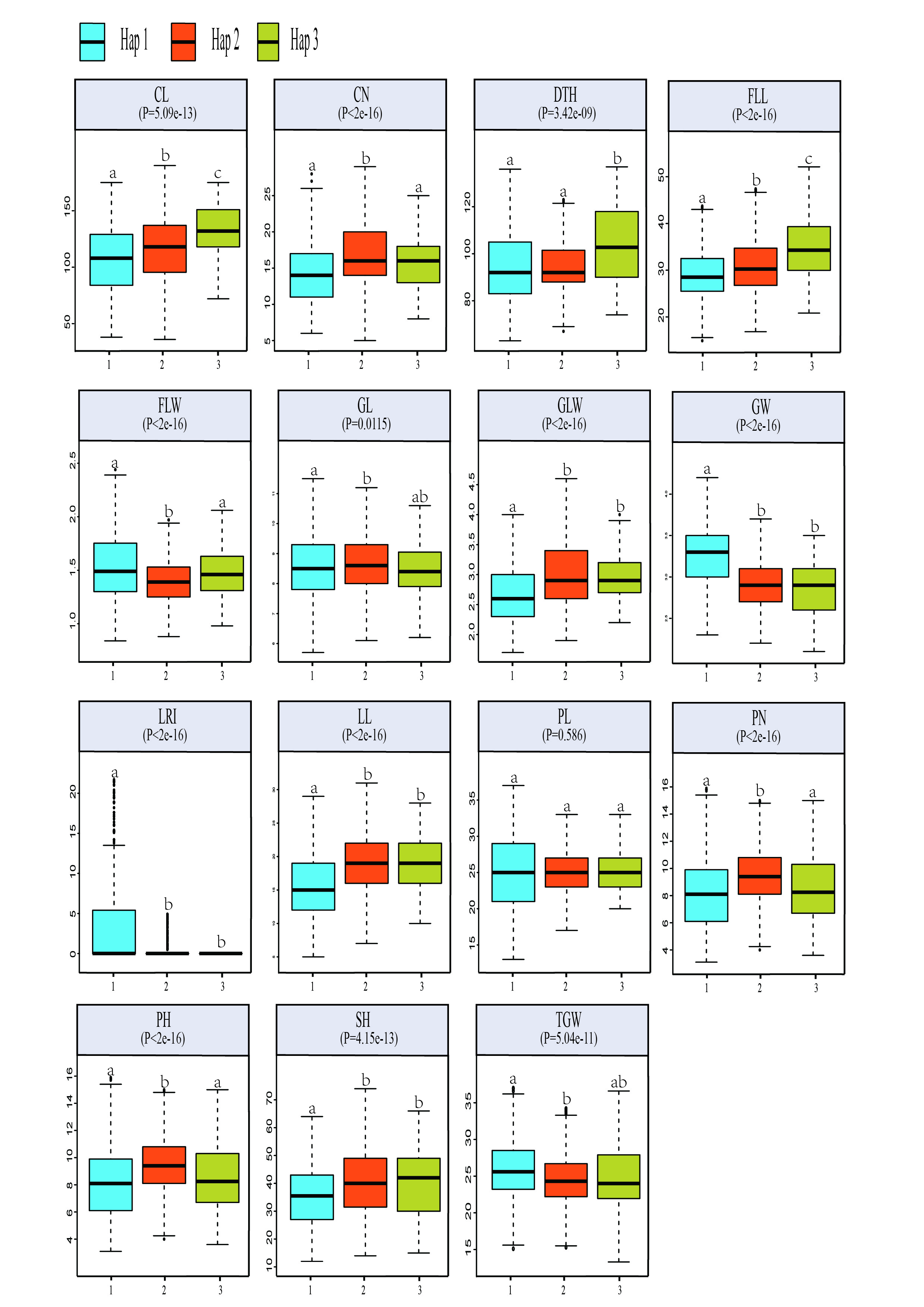

Supplement: Supplementary Figure 1 — Collinear relationship of CXE genes in rice. [file DataSheet3.zip › Supplementary Figure 1-15/Supplementary Figure 11 cxe7.1.jpg]

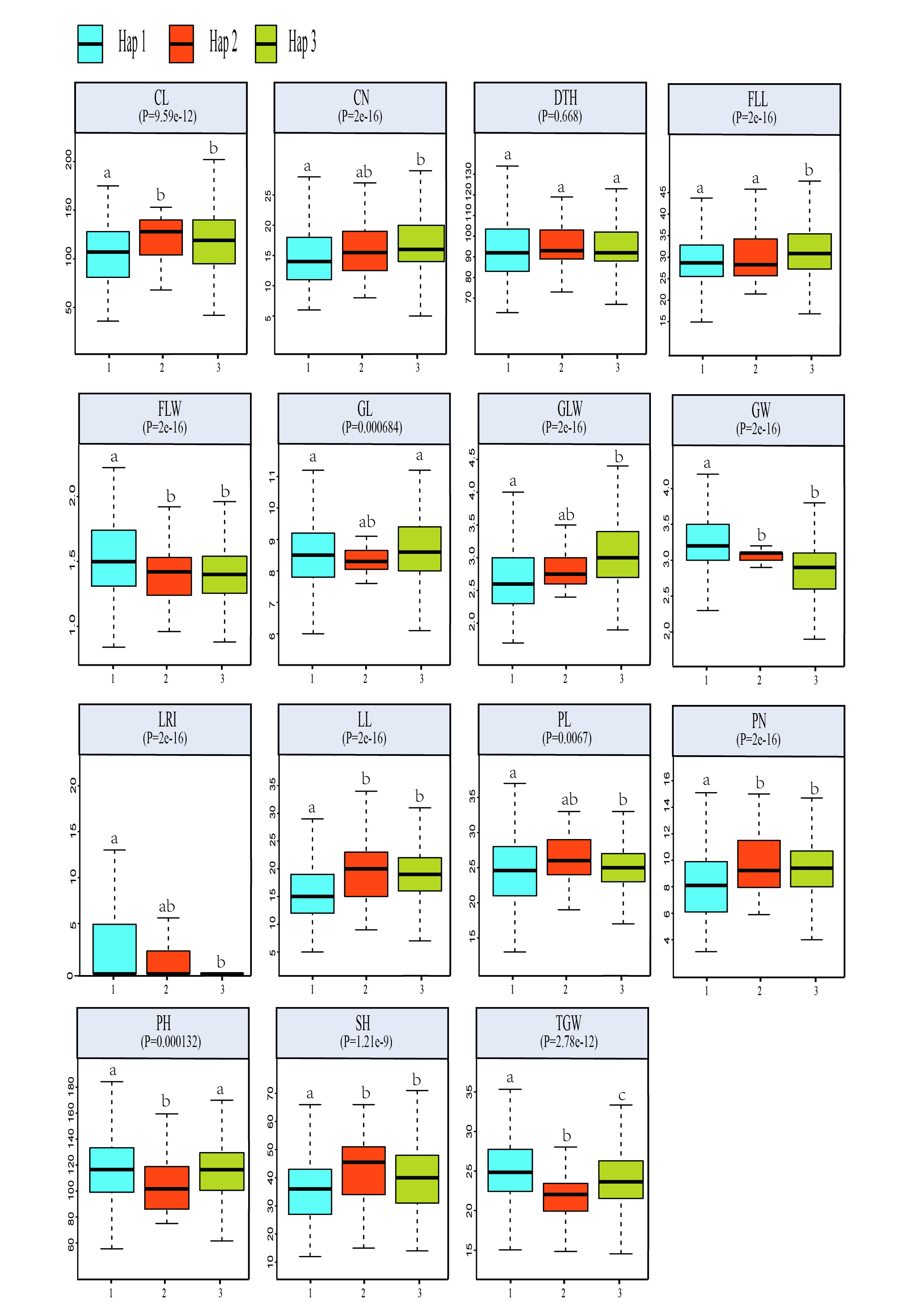

Supplement: Supplementary Figure 1 — Collinear relationship of CXE genes in rice. [file DataSheet3.zip › Supplementary Figure 1-15/Supplementary Figure 12 cxe7.2.jpg]

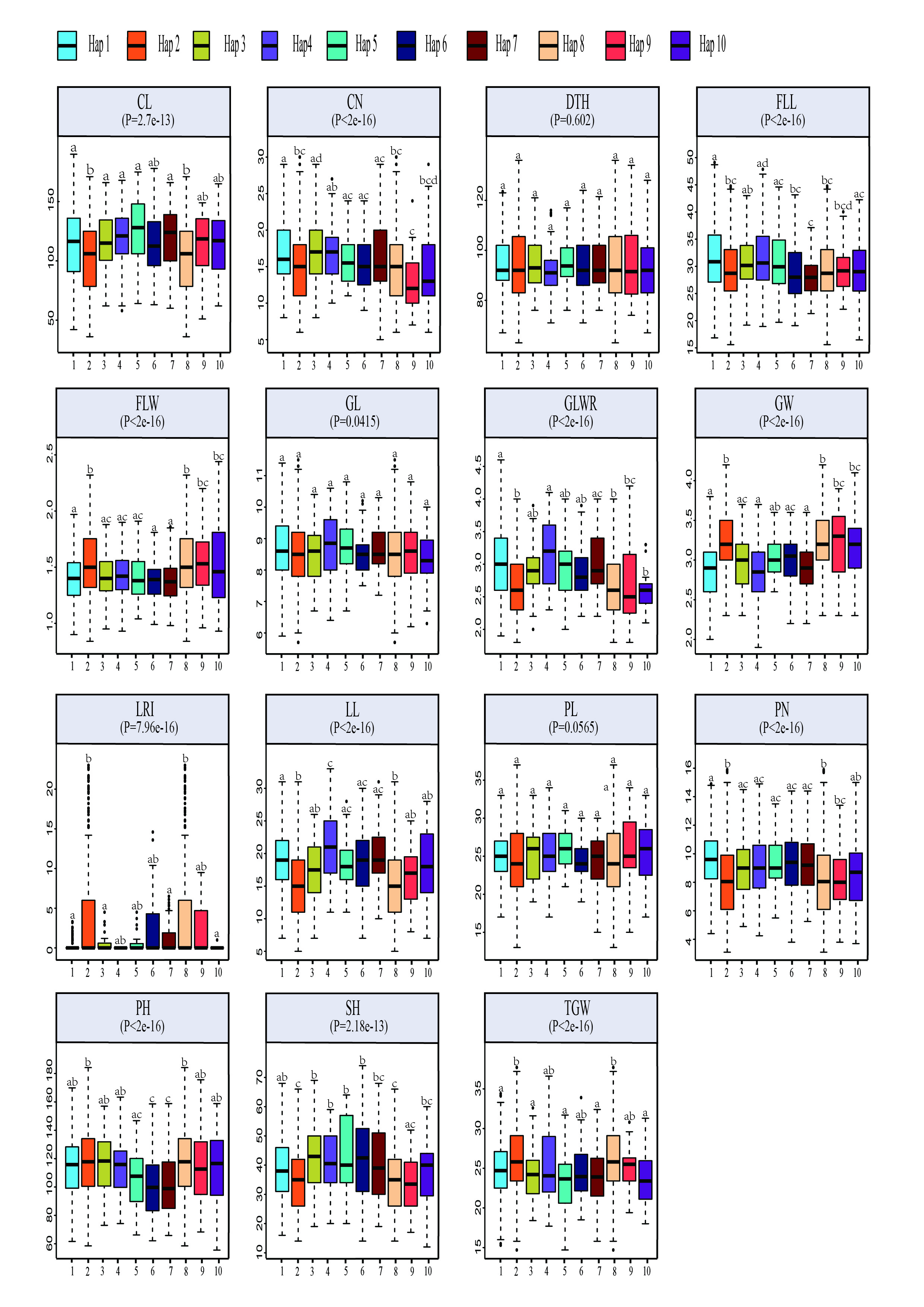

Supplement: Supplementary Figure 1 — Collinear relationship of CXE genes in rice. [file DataSheet3.zip › Supplementary Figure 1-15/Supplementary Figure 13 cxe7.3.jpg]

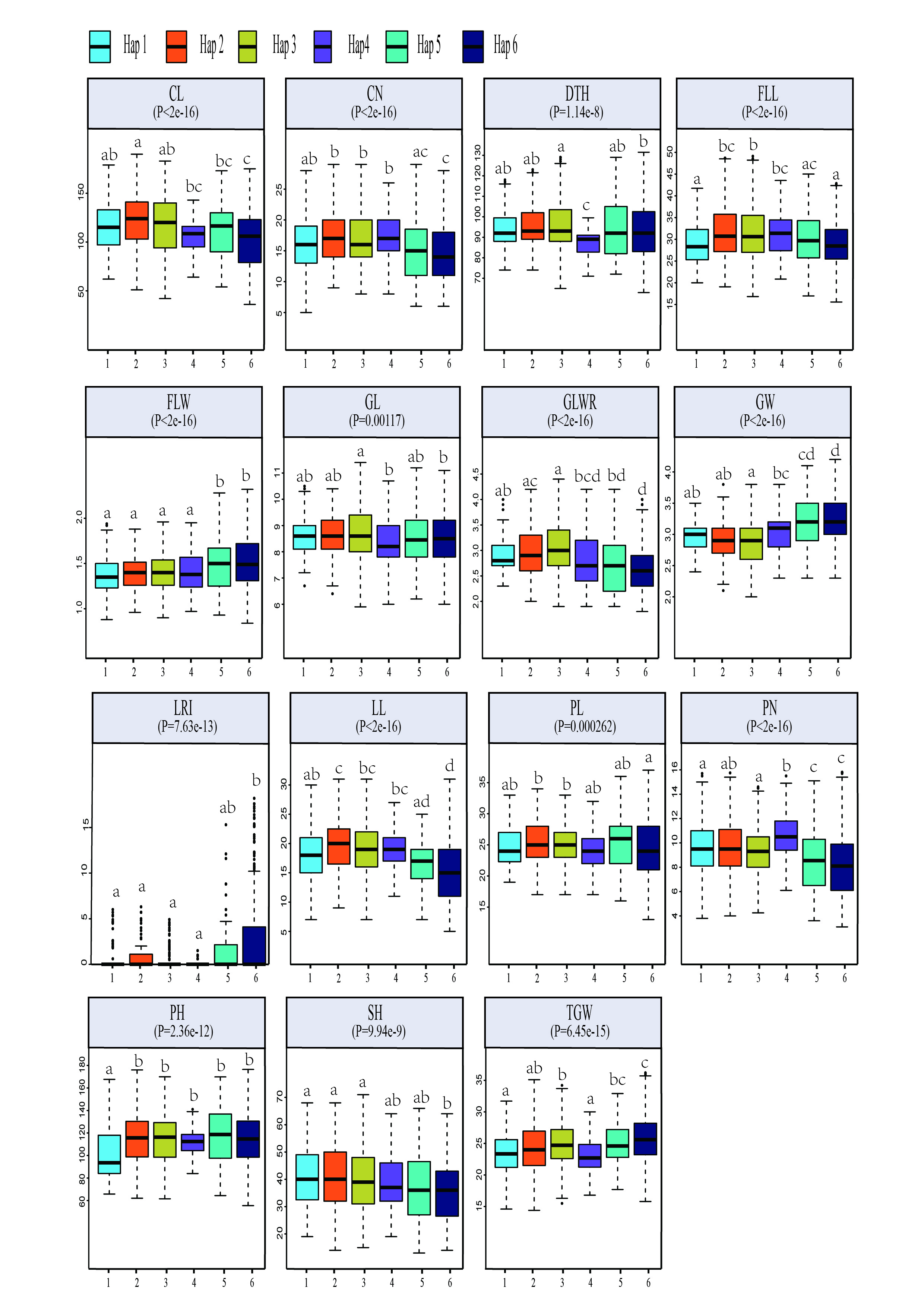

Supplement: Supplementary Figure 1 — Collinear relationship of CXE genes in rice. [file DataSheet3.zip › Supplementary Figure 1-15/Supplementary Figure 14 cxe7.4.jpg]

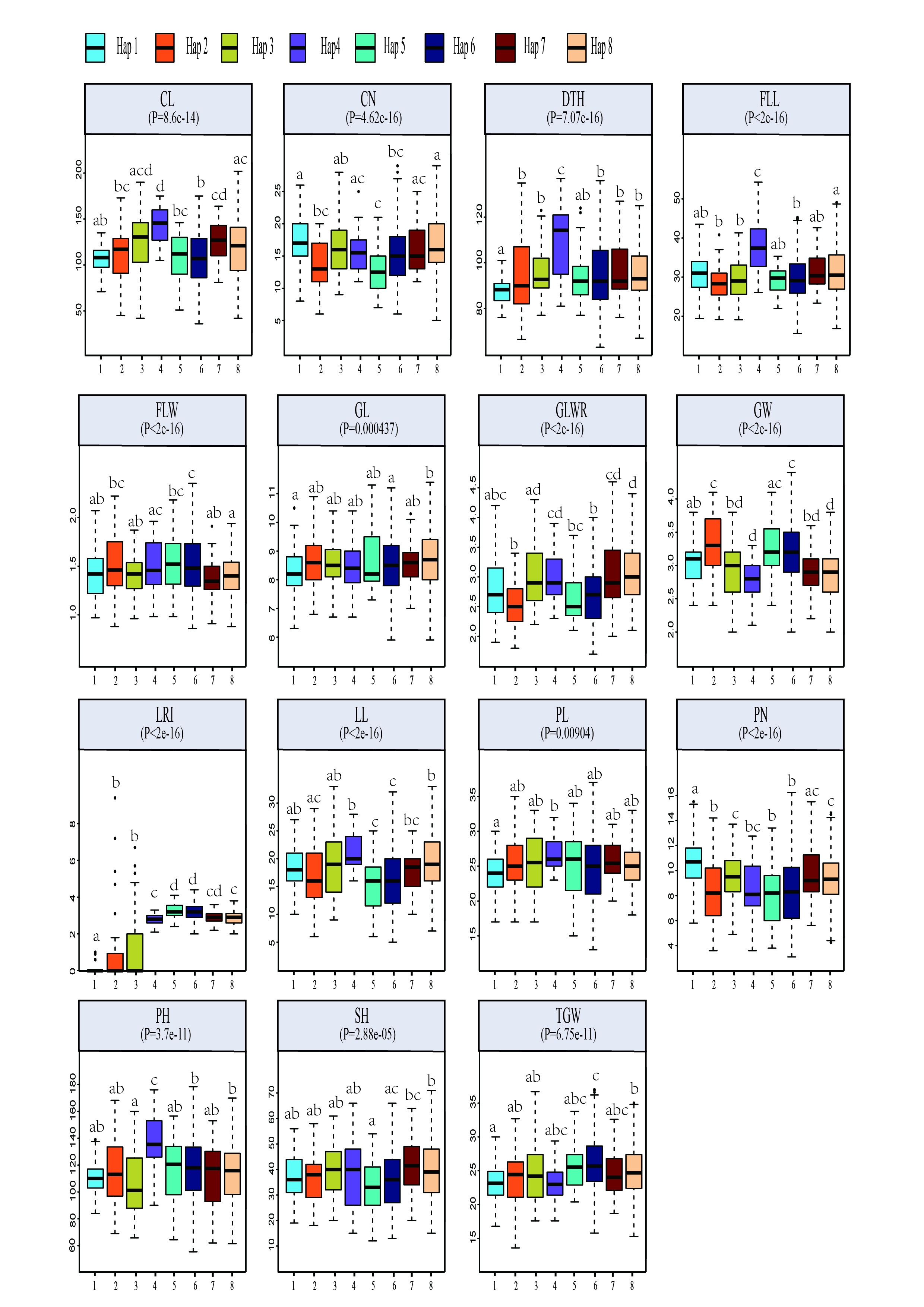

Supplement: Supplementary Figure 1 — Collinear relationship of CXE genes in rice. [file DataSheet3.zip › Supplementary Figure 1-15/Supplementary Figure 15 cxe7.5.jpg]

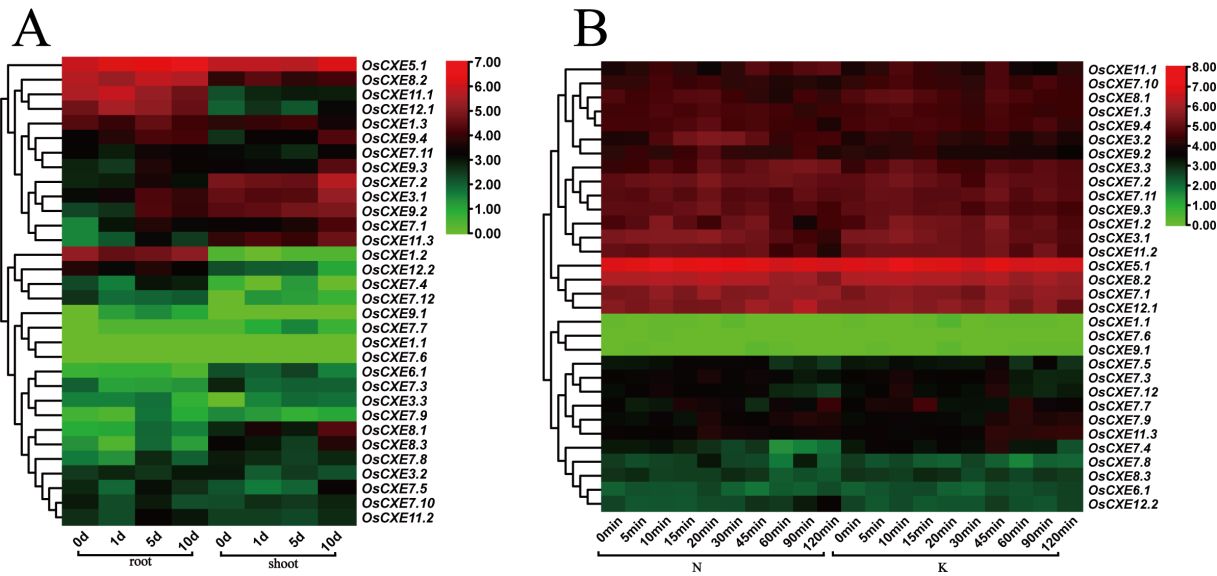

Supplement: Supplementary Figure 1 — Collinear relationship of CXE genes in rice. [file DataSheet3.zip › Supplementary Figure 1-15/Supplementary Figure 2 Analysis of CEXs gene expression in rice.jpg]

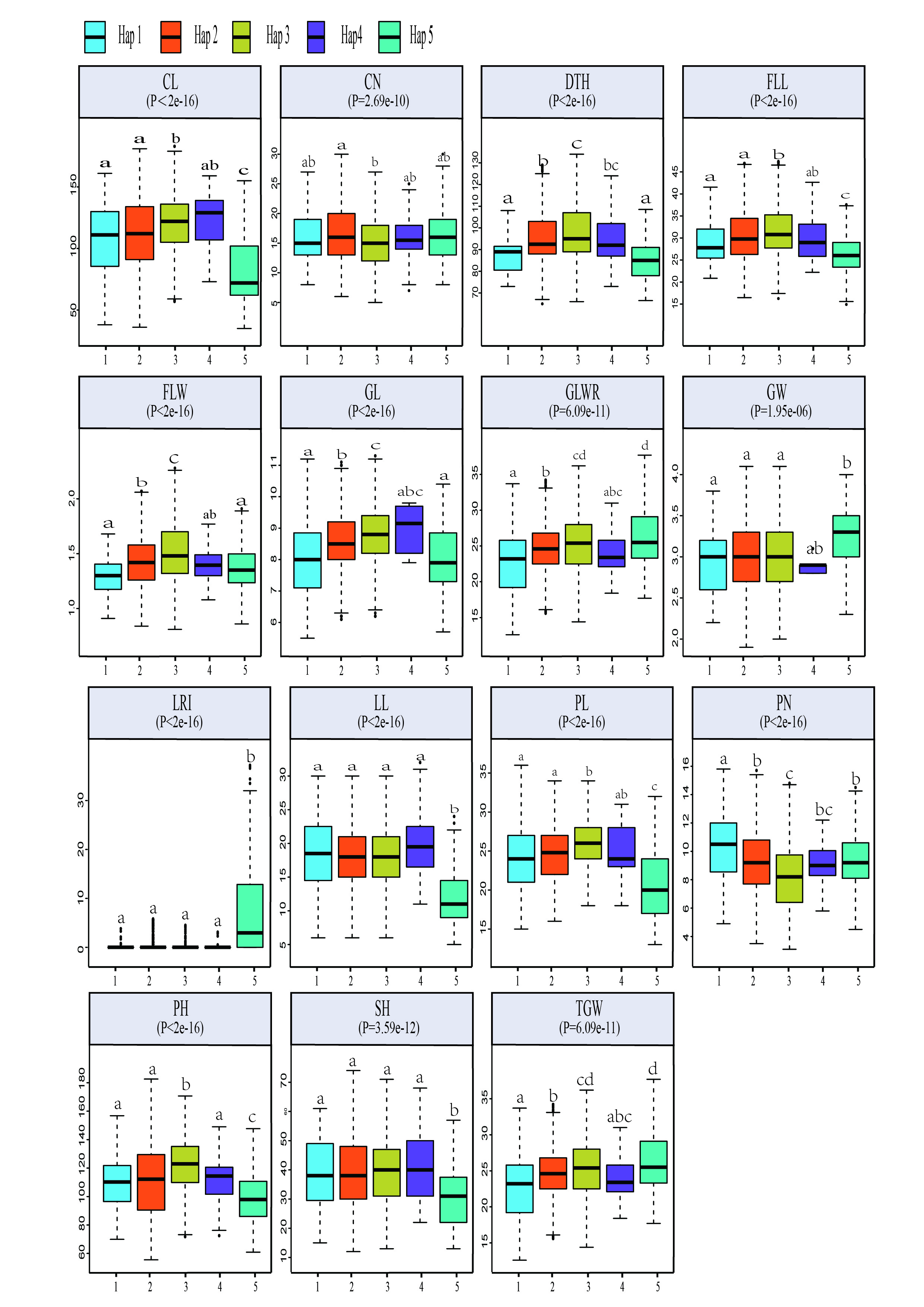

Supplement: Supplementary Figure 1 — Collinear relationship of CXE genes in rice. [file DataSheet3.zip › Supplementary Figure 1-15/Supplementary Figure 3 cxe1.1.jpg]

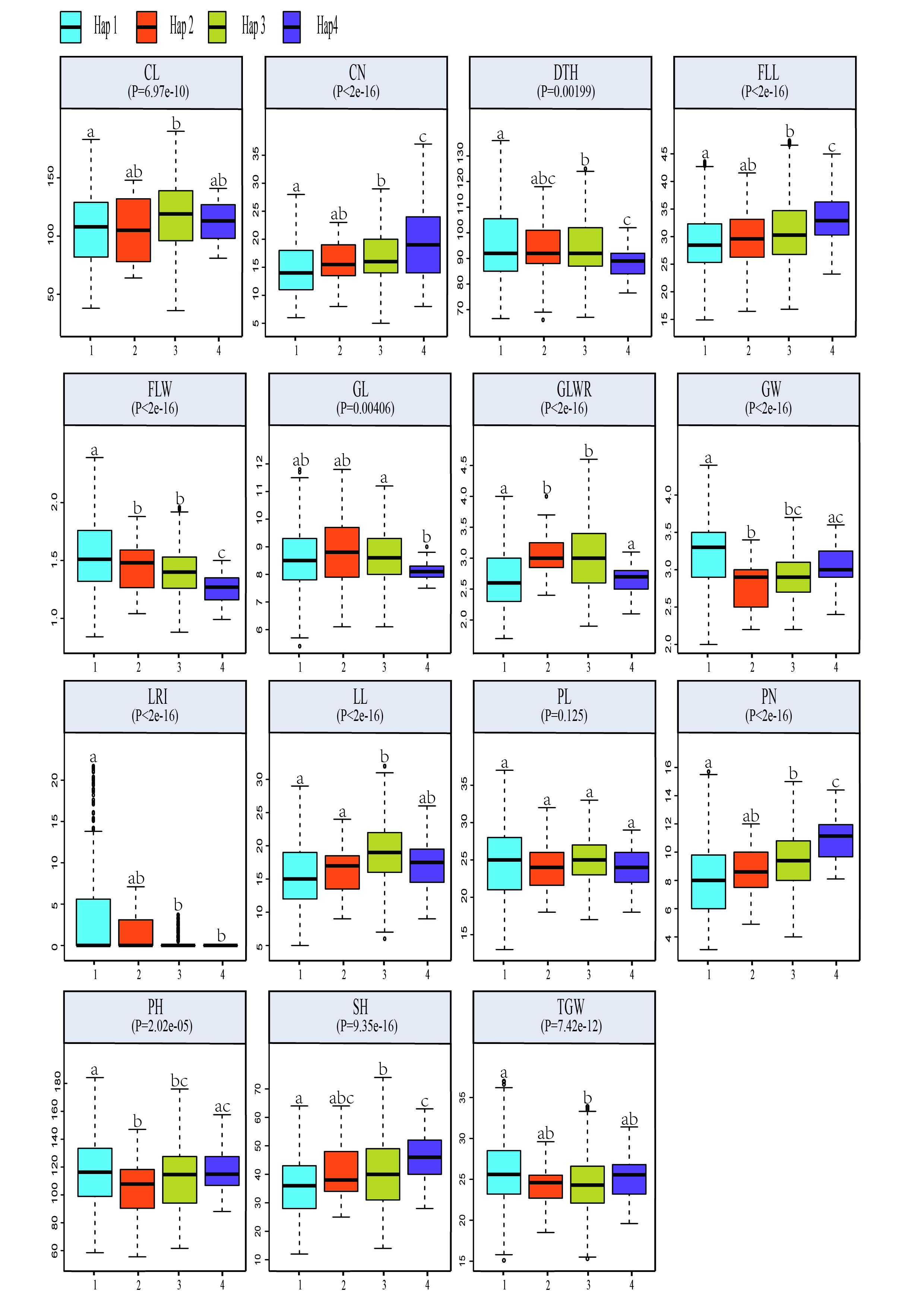

Supplement: Supplementary Figure 1 — Collinear relationship of CXE genes in rice. [file DataSheet3.zip › Supplementary Figure 1-15/Supplementary Figure 4 cxe1.2.jpg]

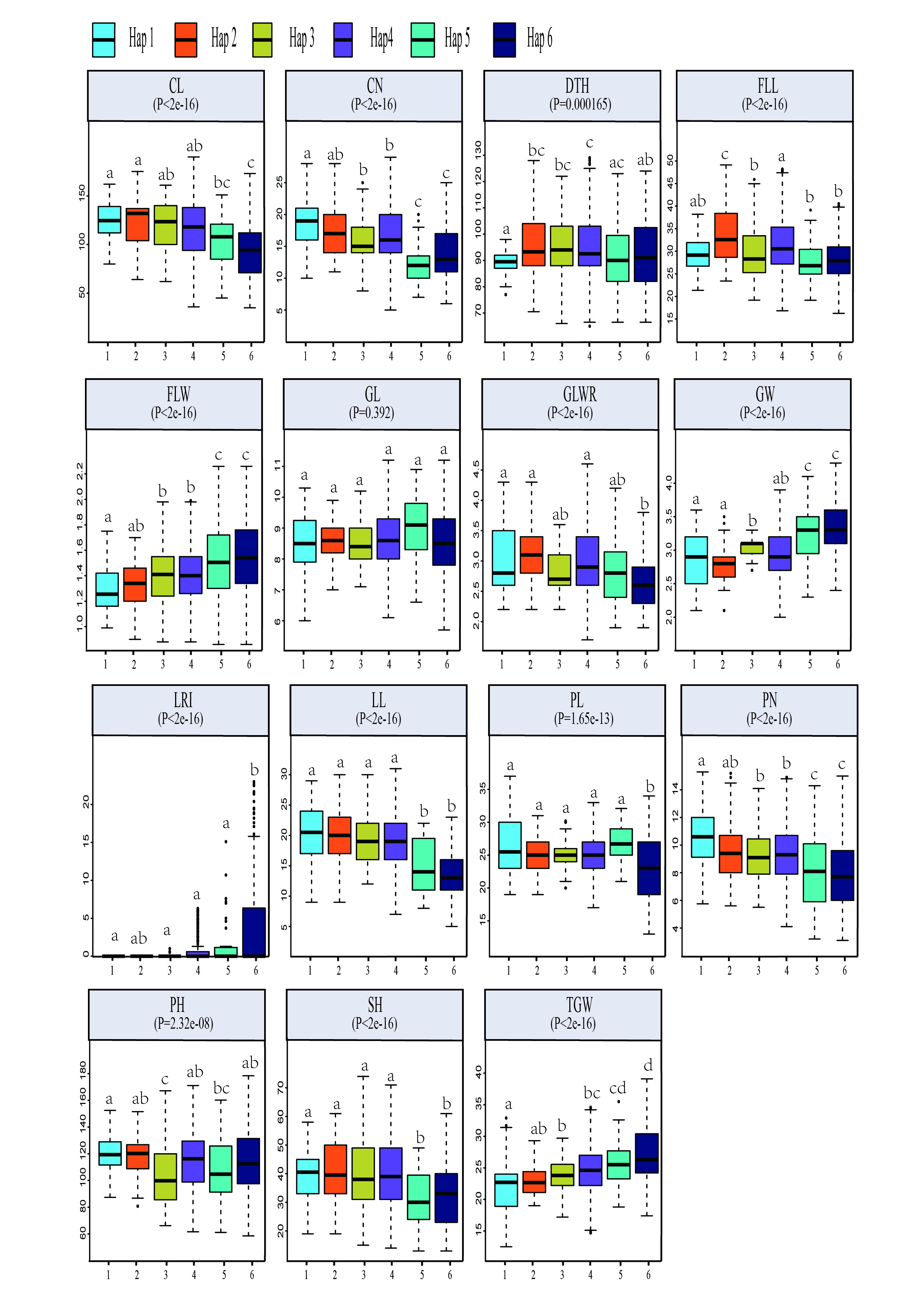

Supplement: Supplementary Figure 1 — Collinear relationship of CXE genes in rice. [file DataSheet3.zip › Supplementary Figure 1-15/Supplementary Figure 5 cxe1.3.jpg]

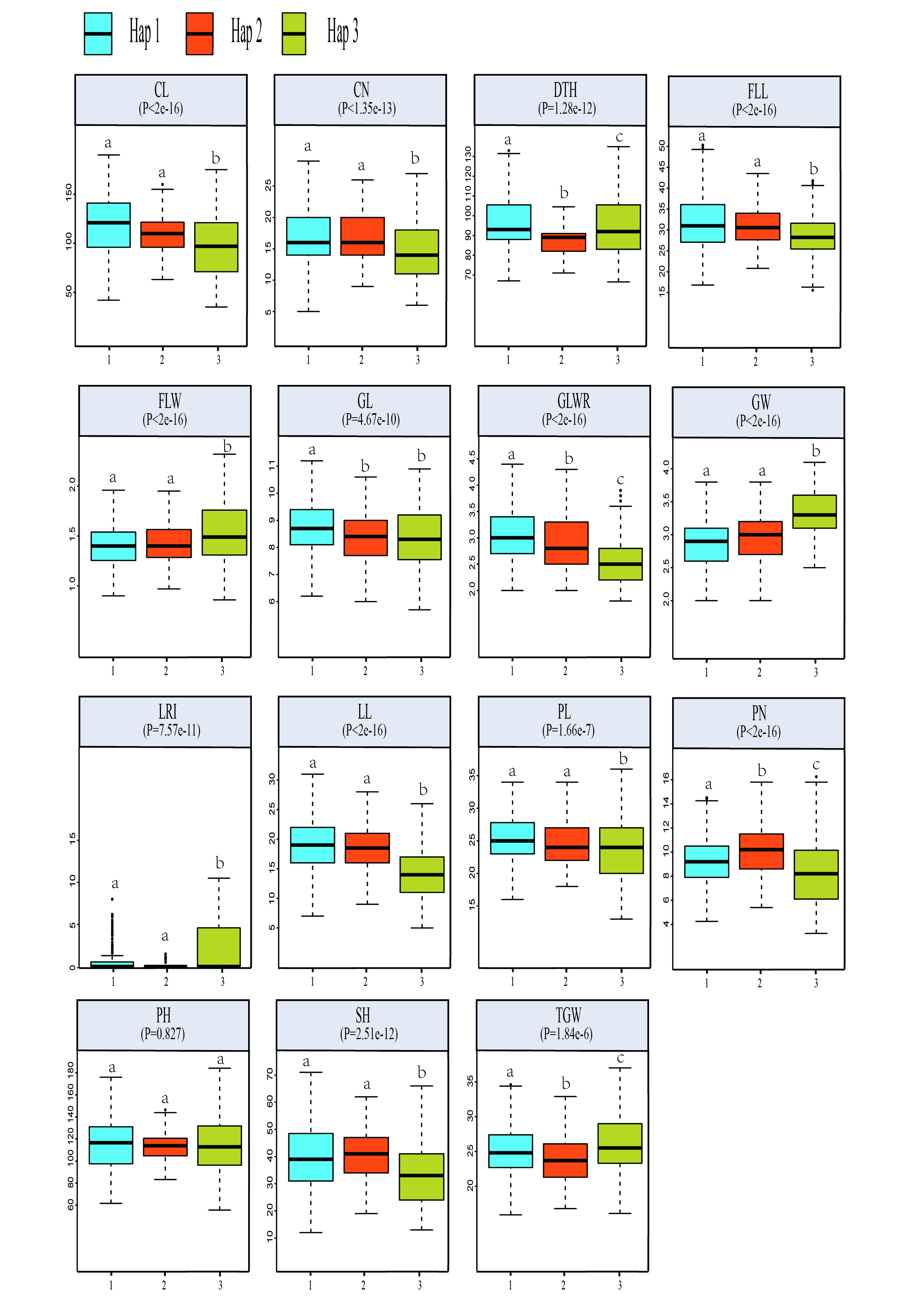

Supplement: Supplementary Figure 1 — Collinear relationship of CXE genes in rice. [file DataSheet3.zip › Supplementary Figure 1-15/Supplementary Figure 6 cxe3.1.jpg]

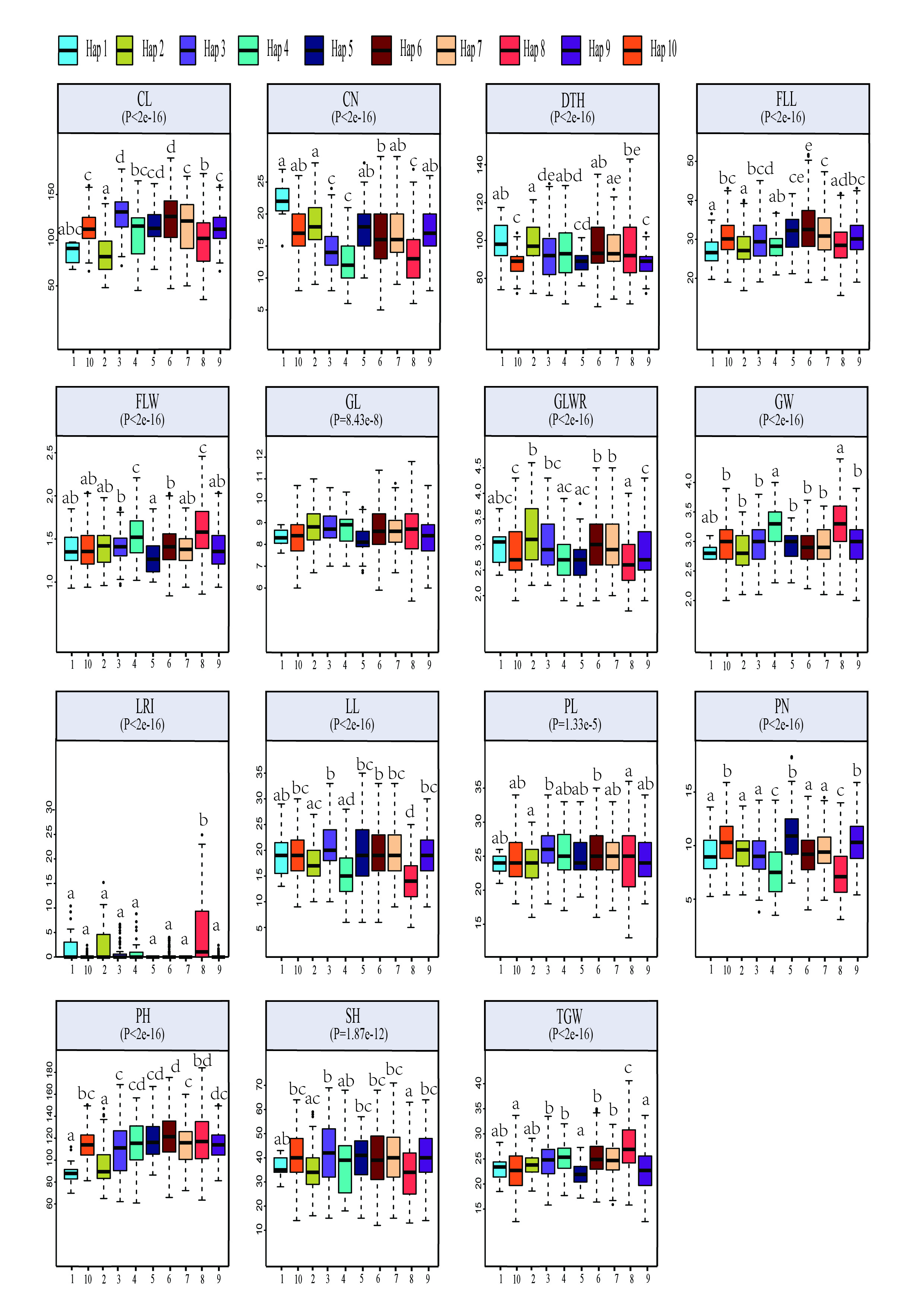

Supplement: Supplementary Figure 1 — Collinear relationship of CXE genes in rice. [file DataSheet3.zip › Supplementary Figure 1-15/Supplementary Figure 7 cxe3.2.jpg]

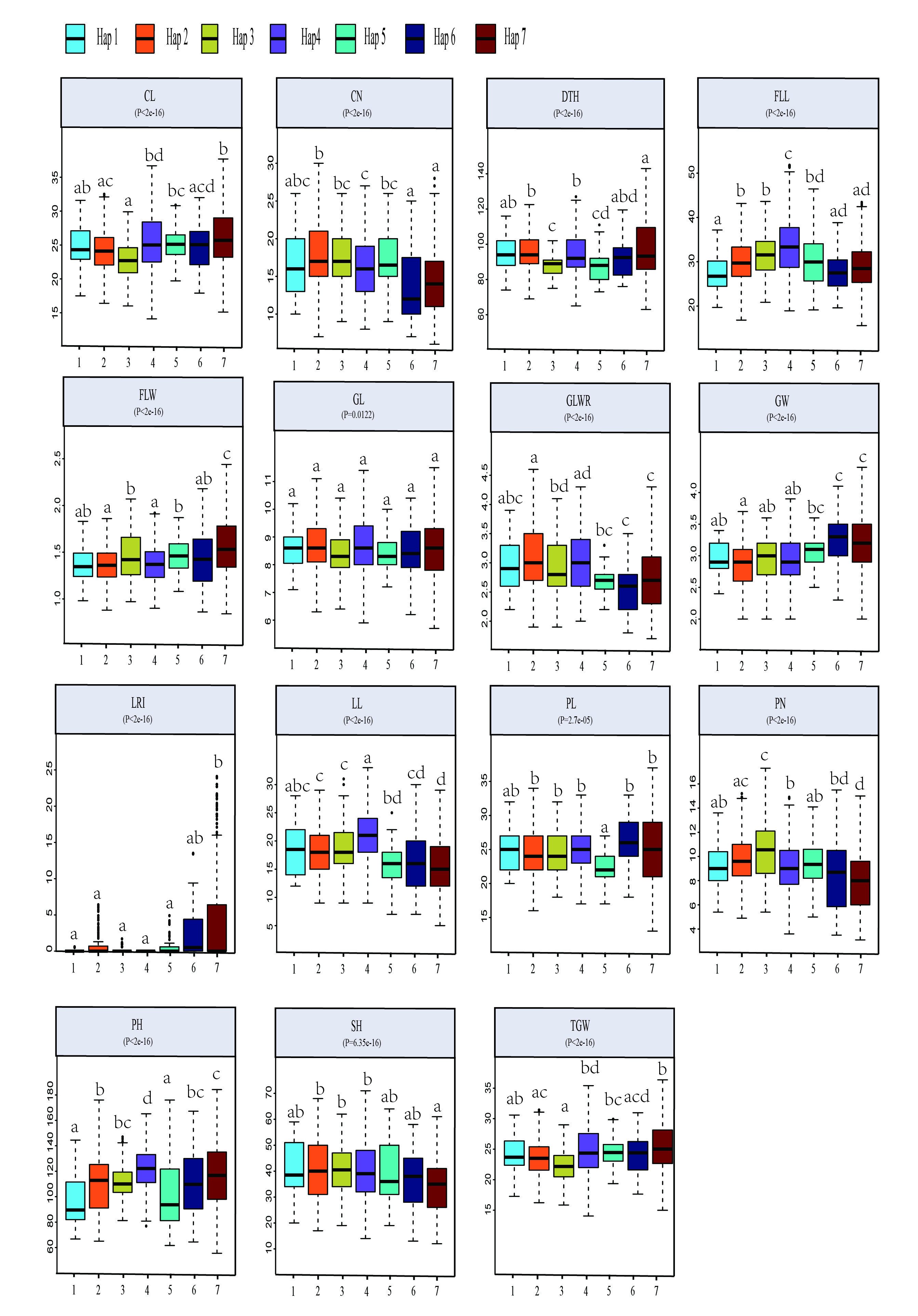

Supplement: Supplementary Figure 1 — Collinear relationship of CXE genes in rice. [file DataSheet3.zip › Supplementary Figure 1-15/Supplementary Figure 8 cxe3.3.jpg]

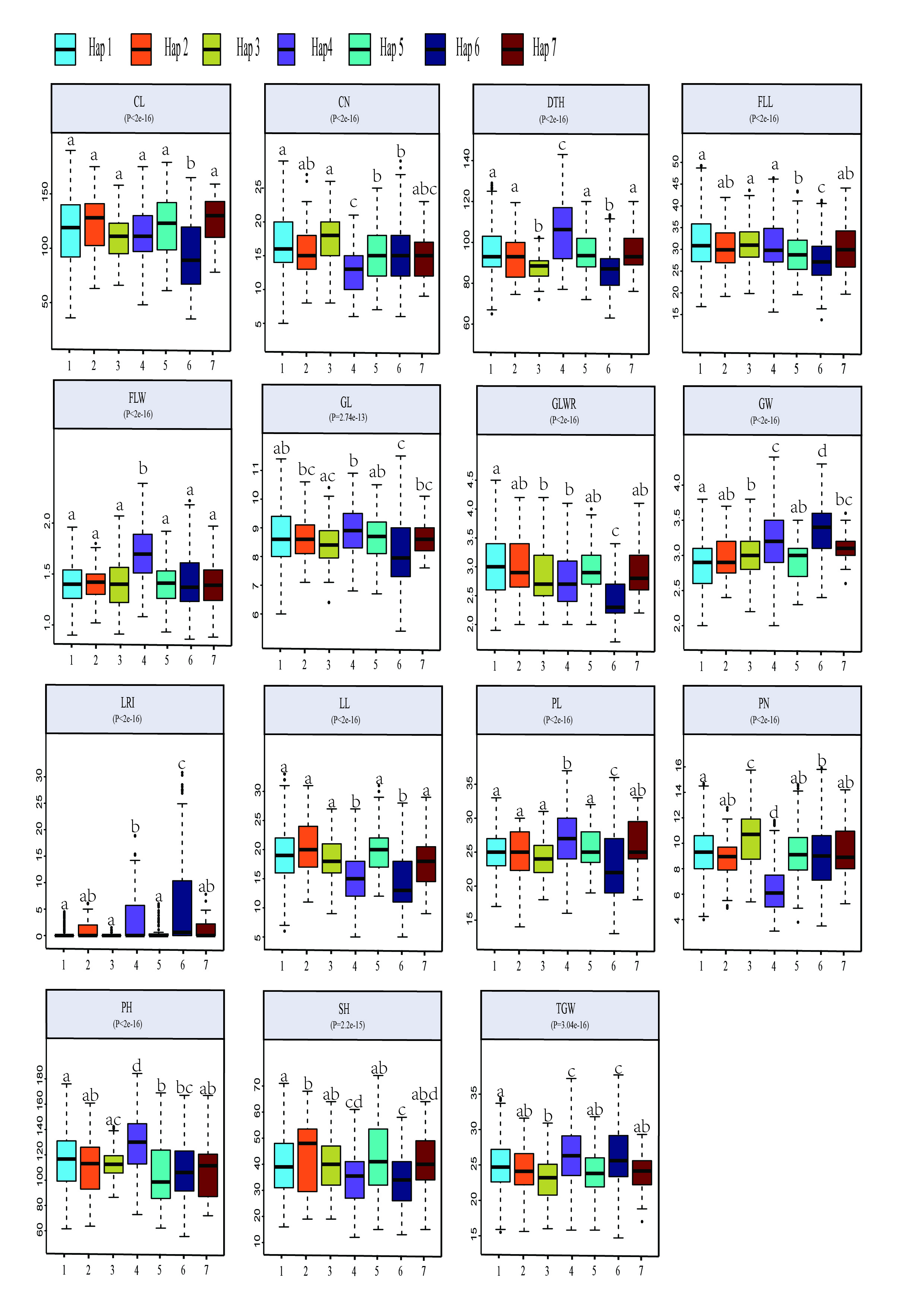

Supplement: Supplementary Figure 1 — Collinear relationship of CXE genes in rice. [file DataSheet3.zip › Supplementary Figure 1-15/Supplementary Figure 9 cxe5.1.jpg]

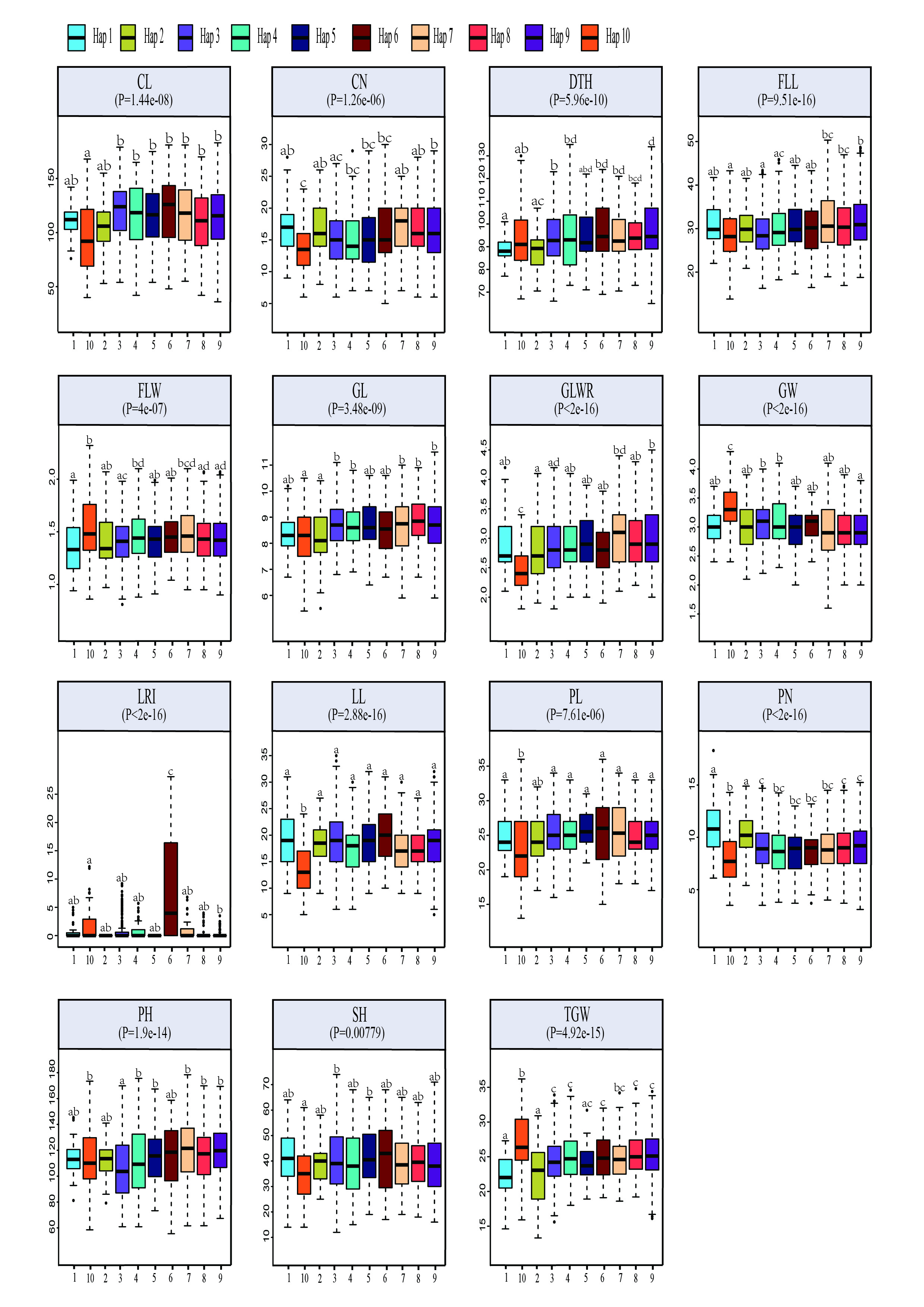

Supplement: Supplementary Figure 16 — Comparison and analysis of 15 agronomic traits among the predominant gcHap, unfavorable gcHap, and major gcHaps of OsCXE7.6. [file DataSheet1.zip › Supplementary Figure 16-30/Supplementary Figure 16 cxe7.6.jpg]

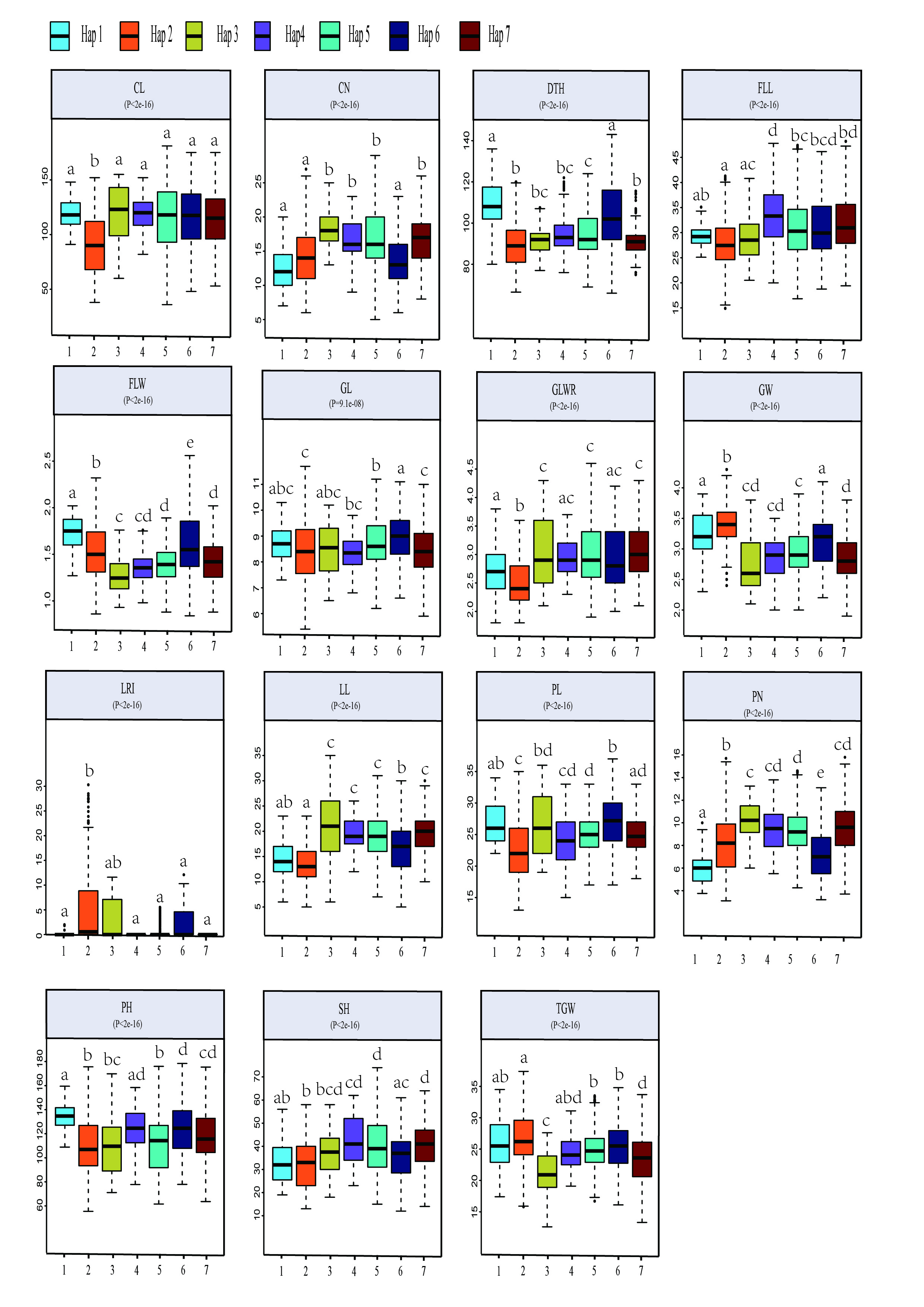

Supplement: Supplementary Figure 16 — Comparison and analysis of 15 agronomic traits among the predominant gcHap, unfavorable gcHap, and major gcHaps of OsCXE7.6. [file DataSheet1.zip › Supplementary Figure 16-30/Supplementary Figure 17 cxe7.7.jpg]

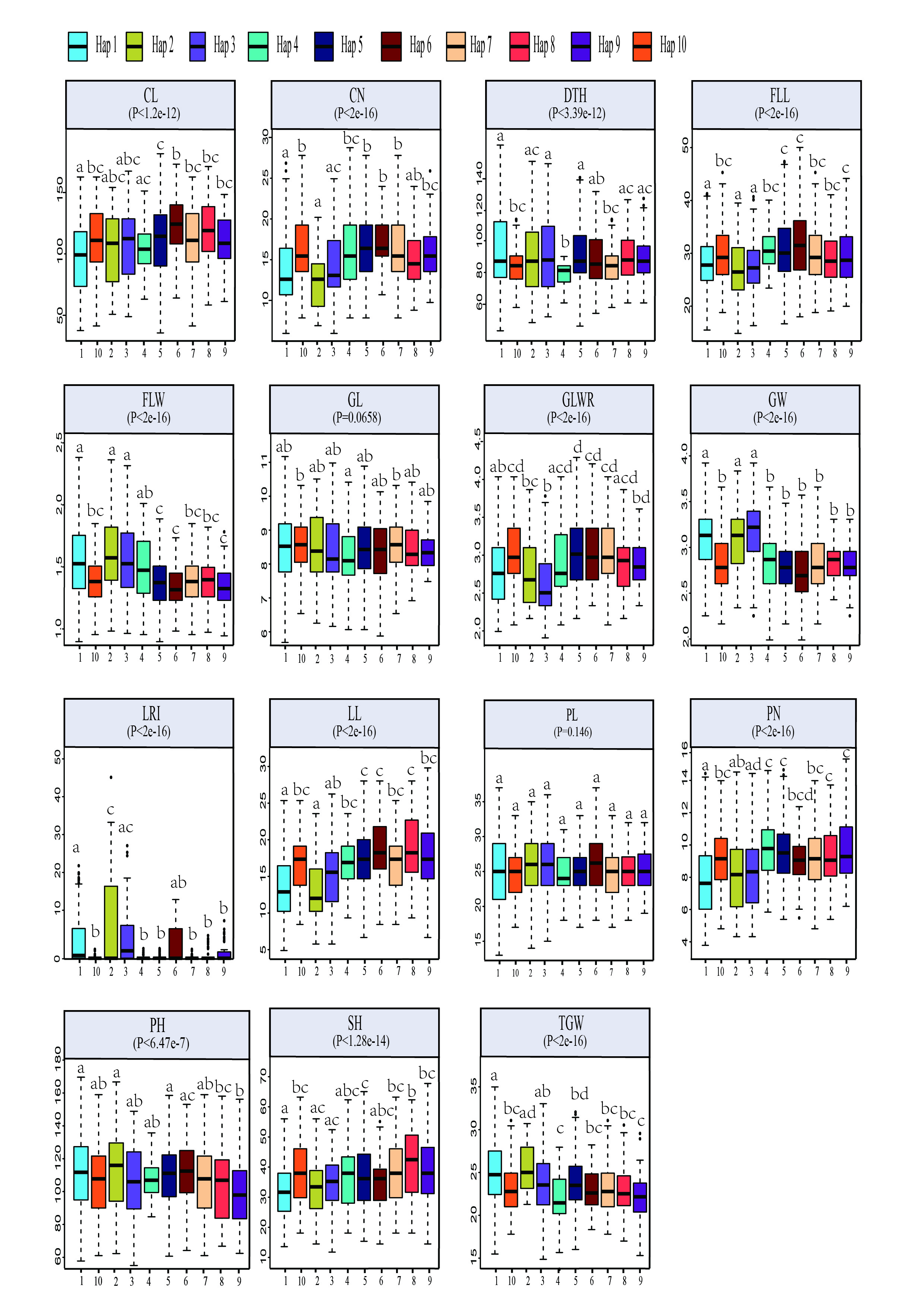

Supplement: Supplementary Figure 16 — Comparison and analysis of 15 agronomic traits among the predominant gcHap, unfavorable gcHap, and major gcHaps of OsCXE7.6. [file DataSheet1.zip › Supplementary Figure 16-30/Supplementary Figure 18 cxe7.8.jpg]

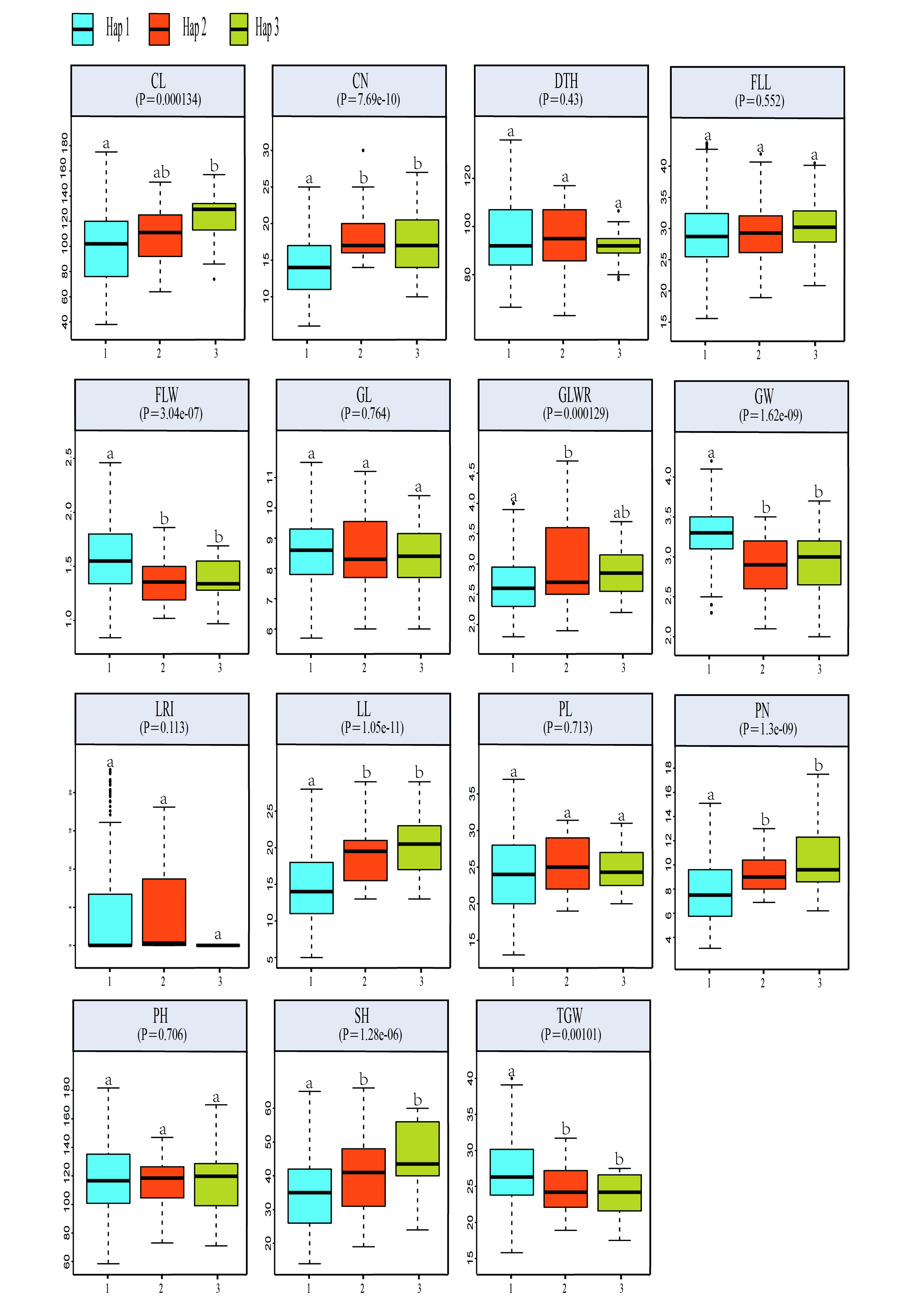

Supplement: Supplementary Figure 16 — Comparison and analysis of 15 agronomic traits among the predominant gcHap, unfavorable gcHap, and major gcHaps of OsCXE7.6. [file DataSheet1.zip › Supplementary Figure 16-30/Supplementary Figure 19 cxe7.9.jpg]

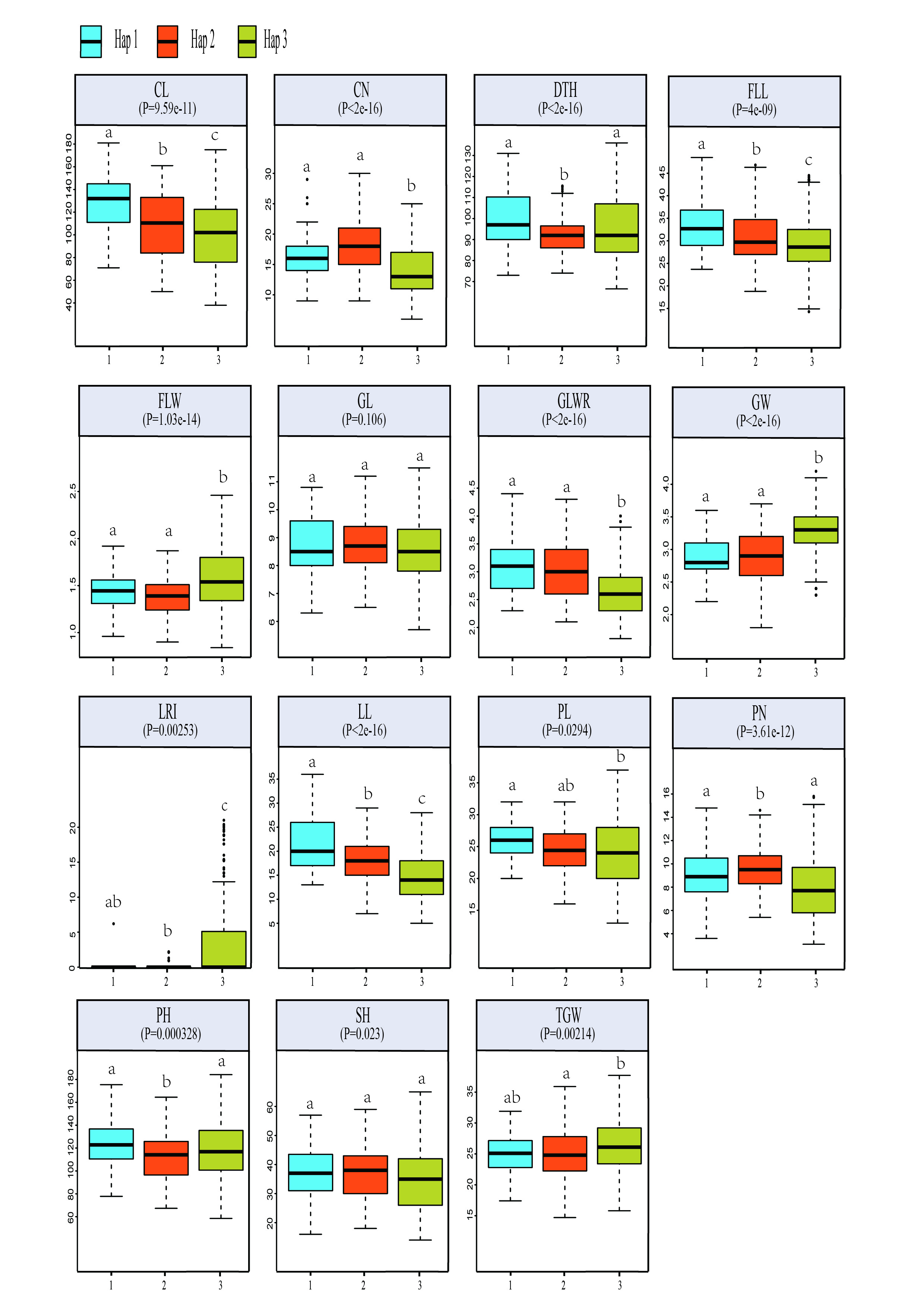

Supplement: Supplementary Figure 16 — Comparison and analysis of 15 agronomic traits among the predominant gcHap, unfavorable gcHap, and major gcHaps of OsCXE7.6. [file DataSheet1.zip › Supplementary Figure 16-30/Supplementary Figure 20 cxe7.10.jpg]

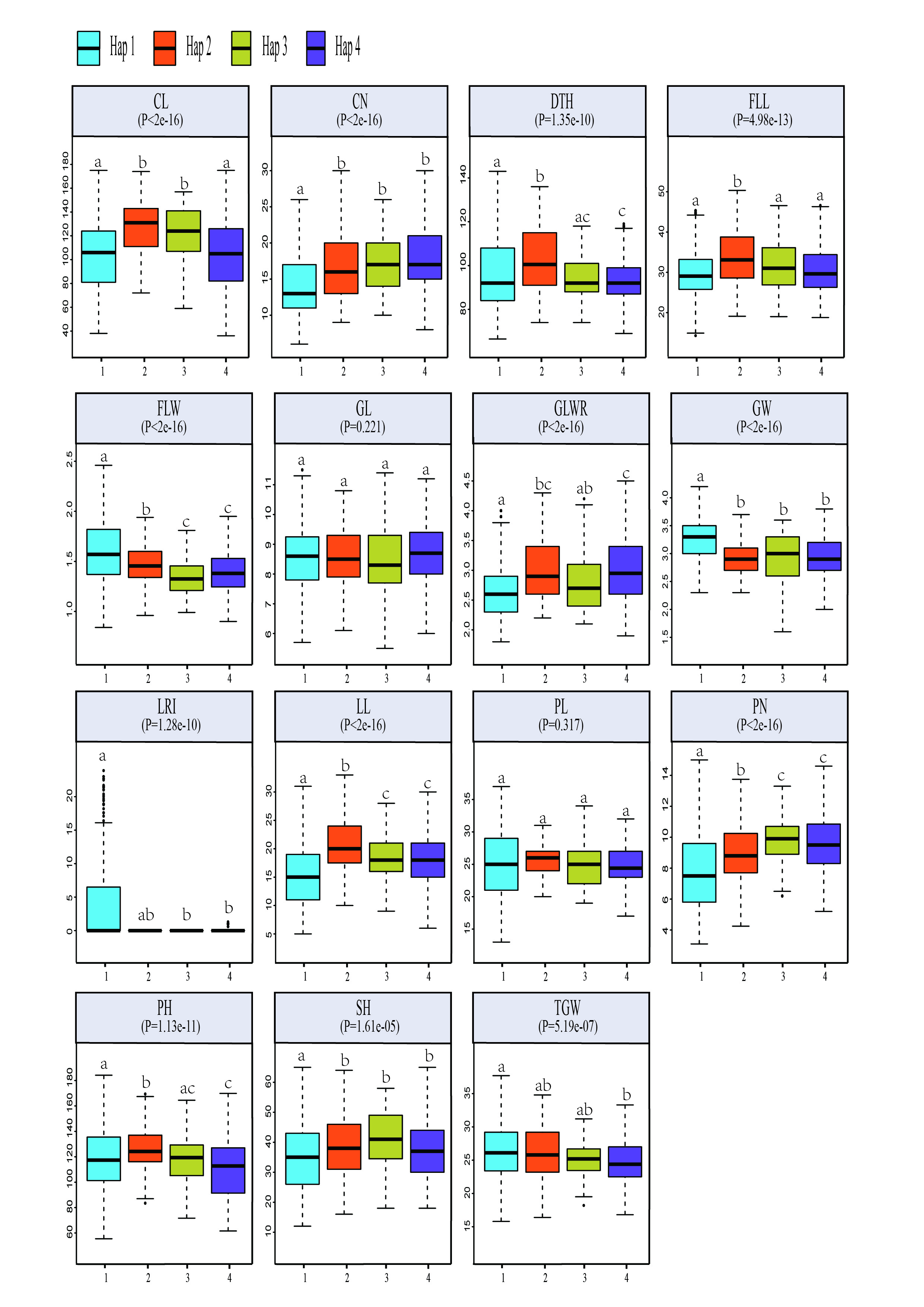

Supplement: Supplementary Figure 16 — Comparison and analysis of 15 agronomic traits among the predominant gcHap, unfavorable gcHap, and major gcHaps of OsCXE7.6. [file DataSheet1.zip › Supplementary Figure 16-30/Supplementary Figure 21 cxe7.11.jpg]

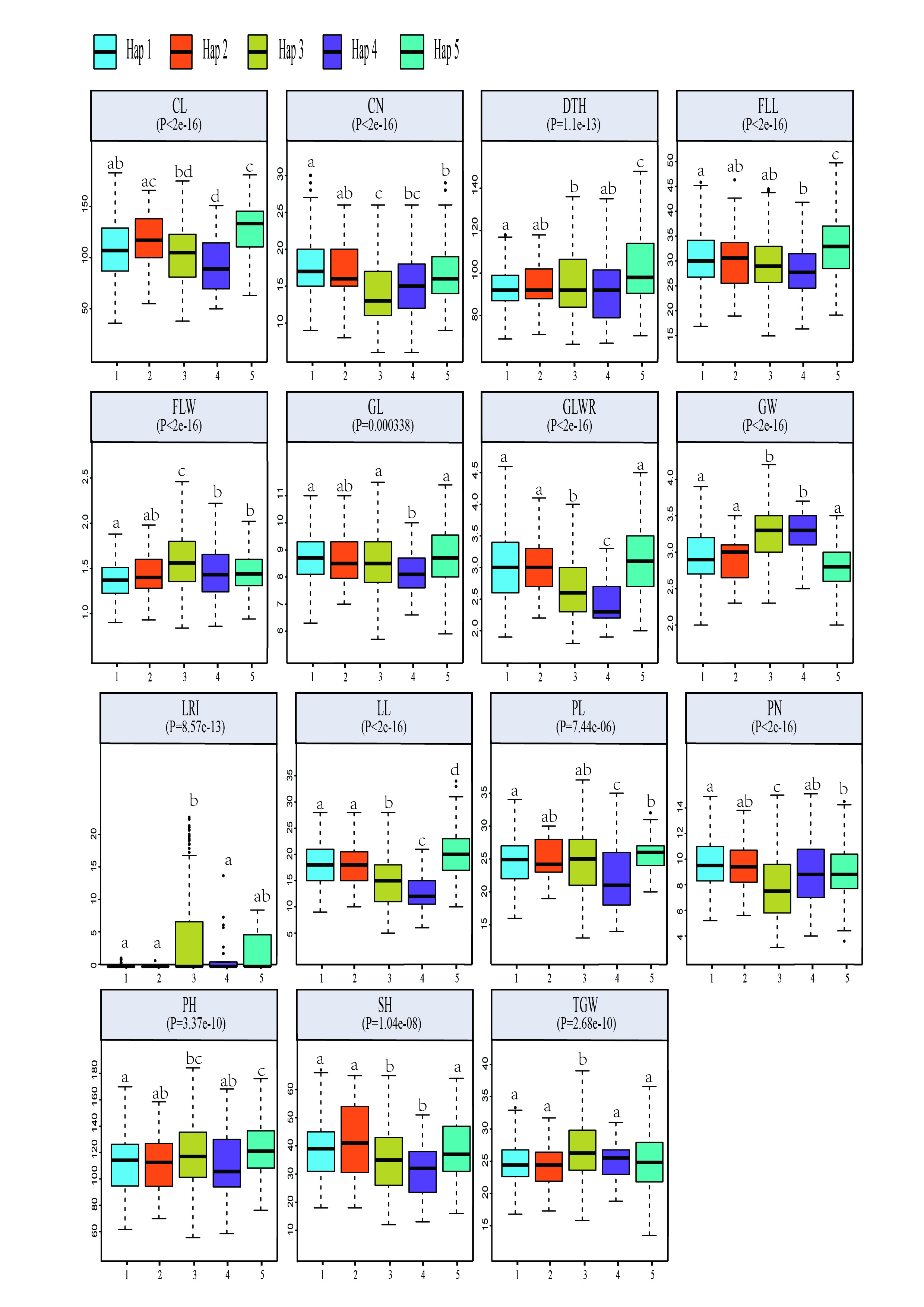

Supplement: Supplementary Figure 16 — Comparison and analysis of 15 agronomic traits among the predominant gcHap, unfavorable gcHap, and major gcHaps of OsCXE7.6. [file DataSheet1.zip › Supplementary Figure 16-30/Supplementary Figure 22 cxe7.12.jpg]

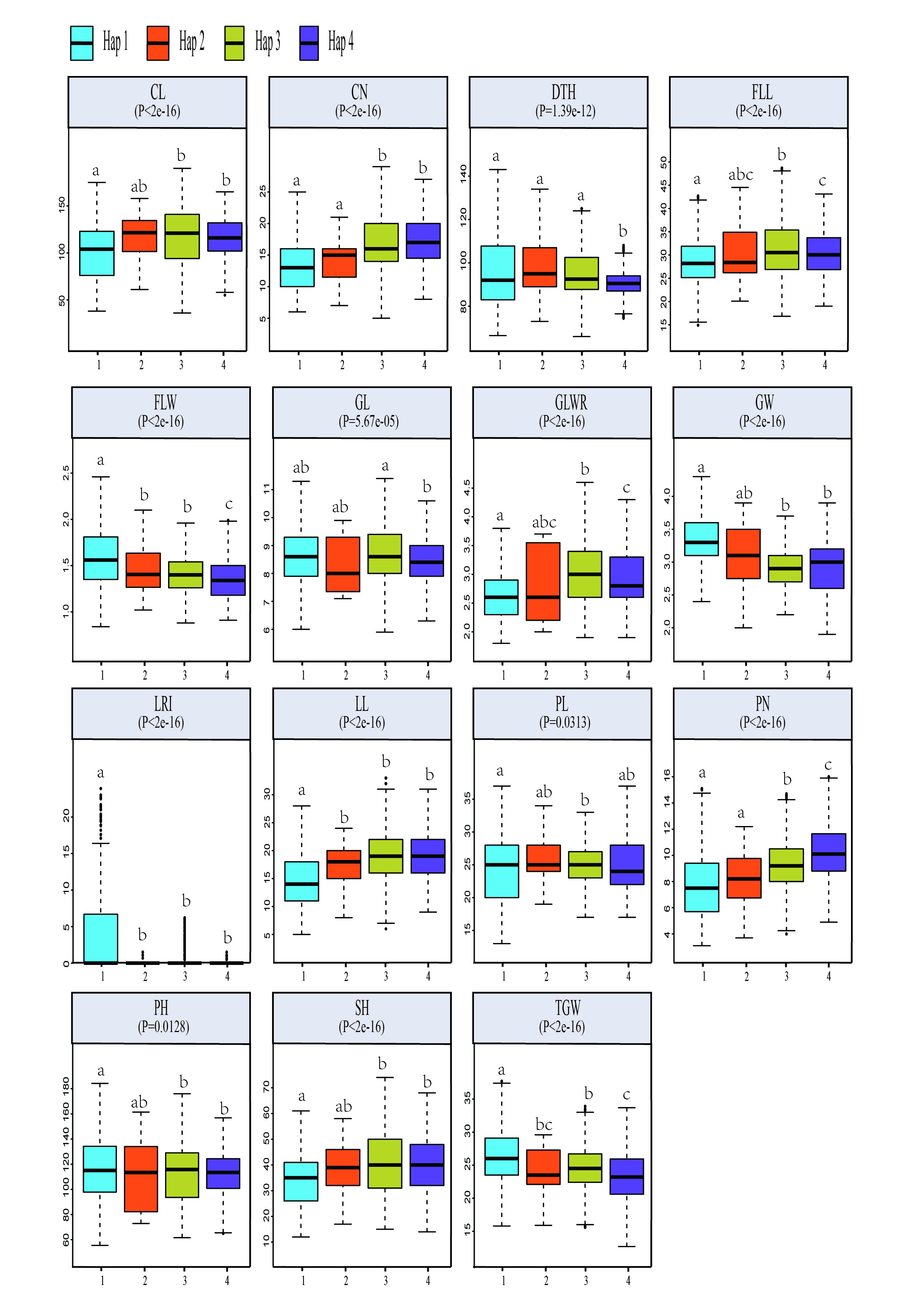

Supplement: Supplementary Figure 16 — Comparison and analysis of 15 agronomic traits among the predominant gcHap, unfavorable gcHap, and major gcHaps of OsCXE7.6. [file DataSheet1.zip › Supplementary Figure 16-30/Supplementary Figure 23 cxe8.1.jpg]

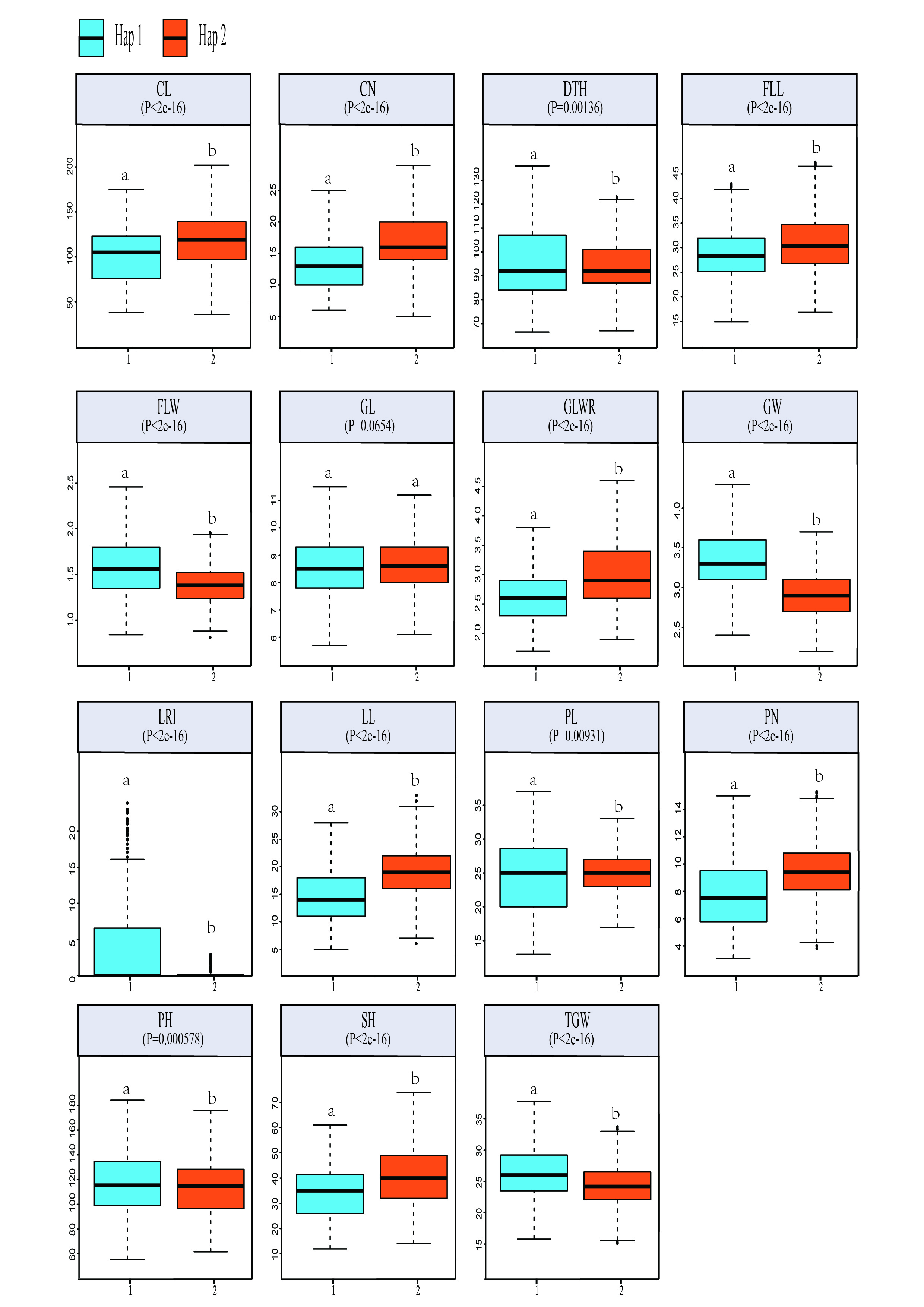

Supplement: Supplementary Figure 16 — Comparison and analysis of 15 agronomic traits among the predominant gcHap, unfavorable gcHap, and major gcHaps of OsCXE7.6. [file DataSheet1.zip › Supplementary Figure 16-30/Supplementary Figure 24 cxe8.2.jpg]

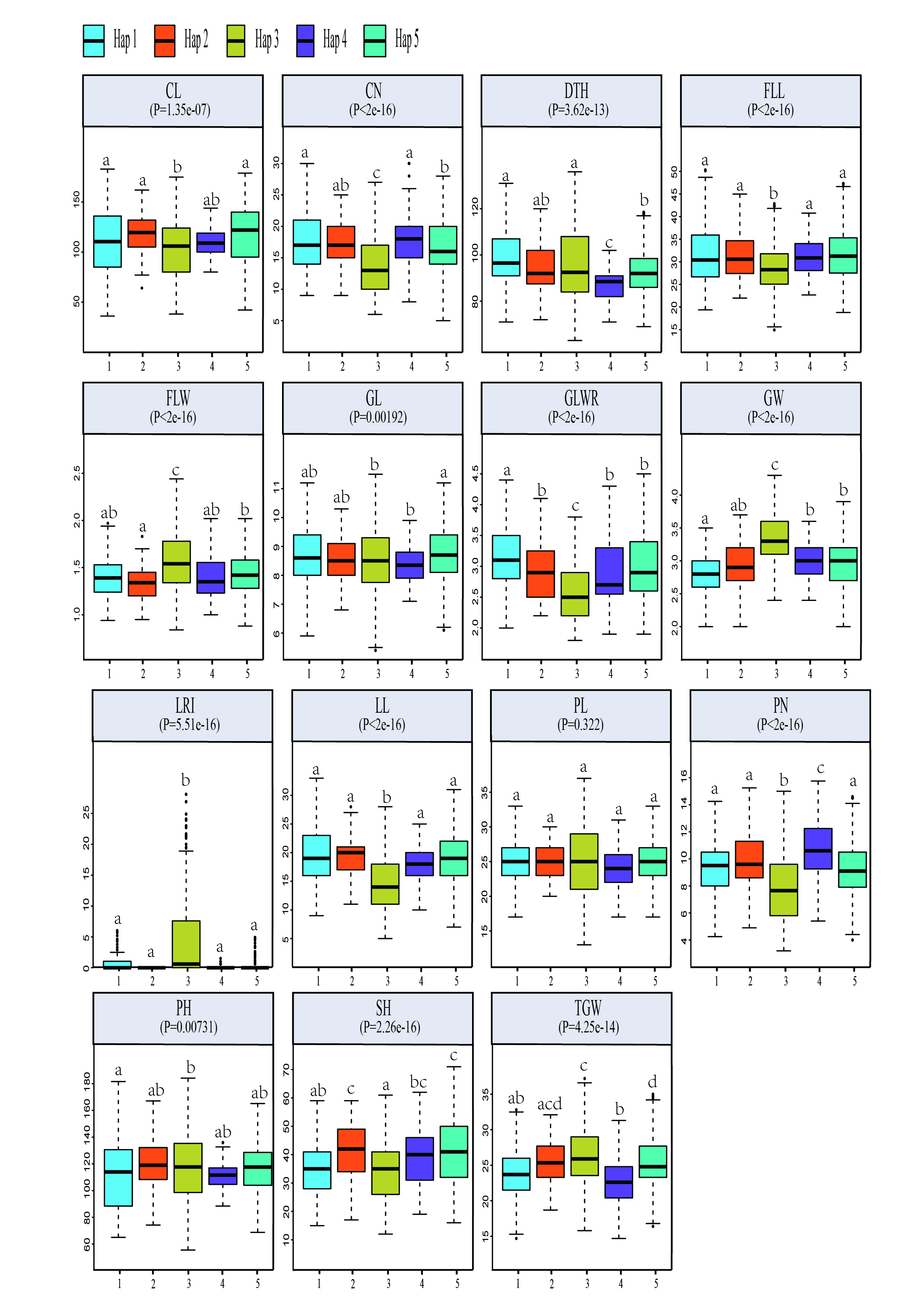

Supplement: Supplementary Figure 16 — Comparison and analysis of 15 agronomic traits among the predominant gcHap, unfavorable gcHap, and major gcHaps of OsCXE7.6. [file DataSheet1.zip › Supplementary Figure 16-30/Supplementary Figure 25 cxe8.3.jpg]

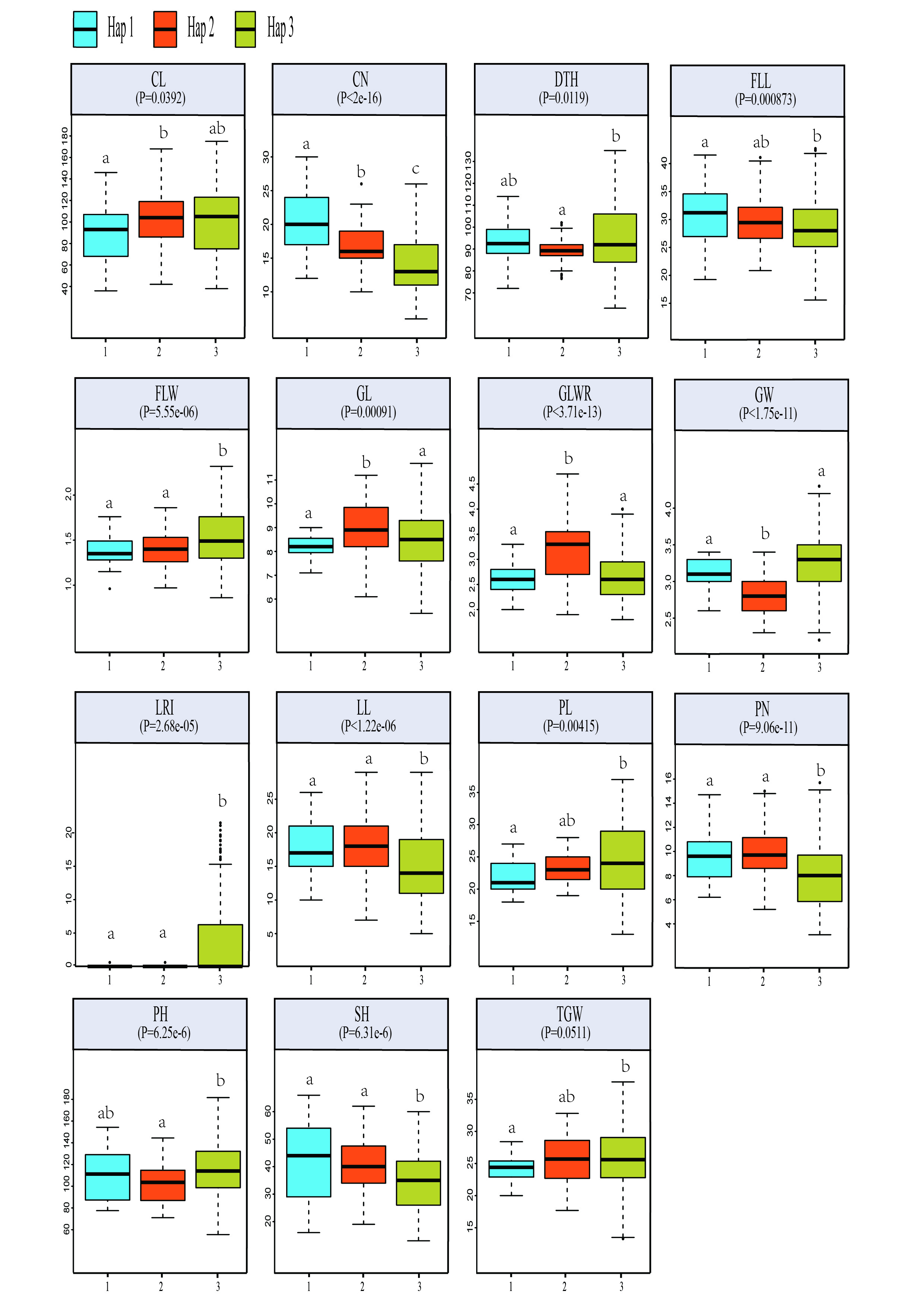

Supplement: Supplementary Figure 16 — Comparison and analysis of 15 agronomic traits among the predominant gcHap, unfavorable gcHap, and major gcHaps of OsCXE7.6. [file DataSheet1.zip › Supplementary Figure 16-30/Supplementary Figure 26 cxe9.1.jpg]

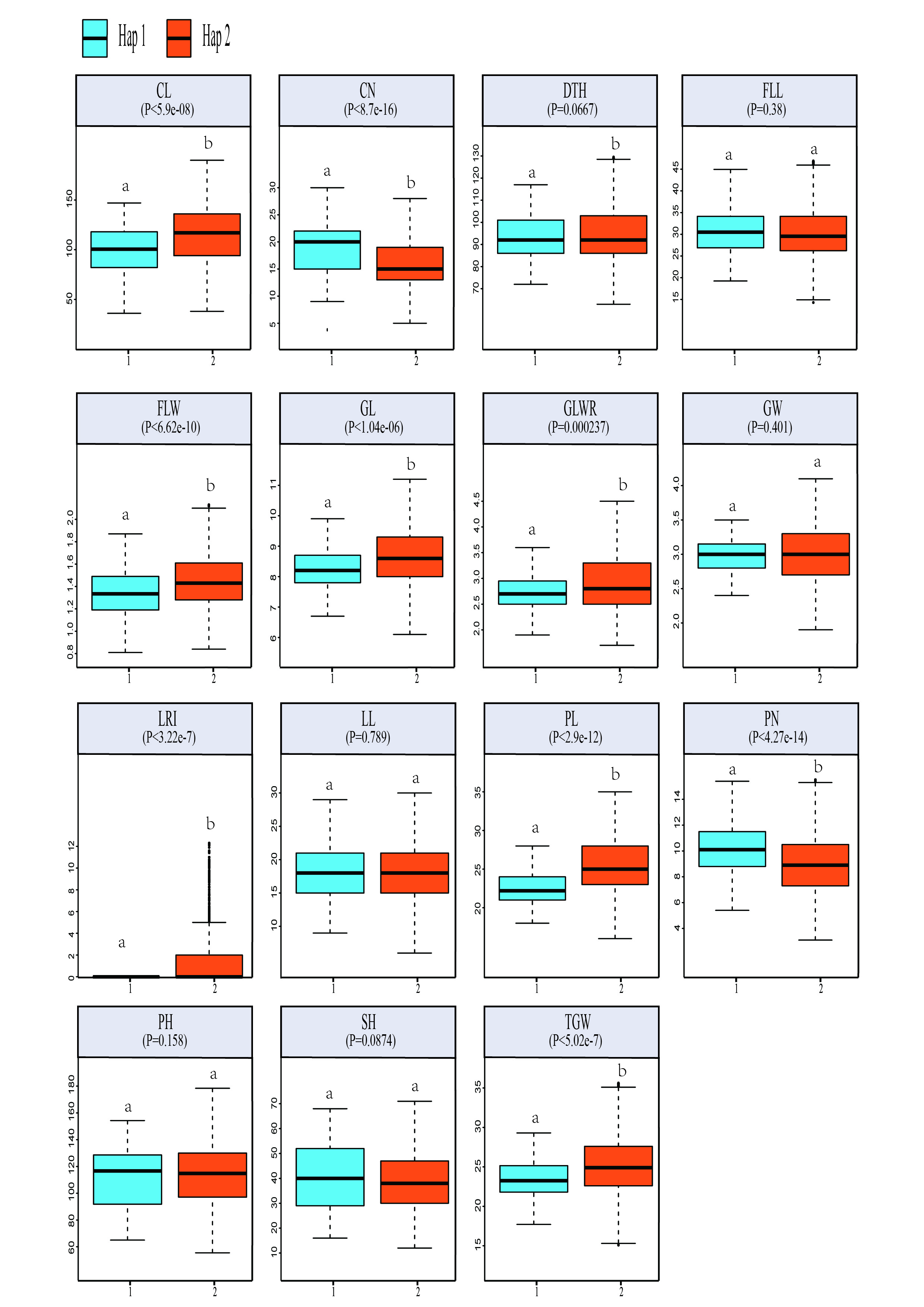

Supplement: Supplementary Figure 16 — Comparison and analysis of 15 agronomic traits among the predominant gcHap, unfavorable gcHap, and major gcHaps of OsCXE7.6. [file DataSheet1.zip › Supplementary Figure 16-30/Supplementary Figure 27 cxe9.2.jpg]

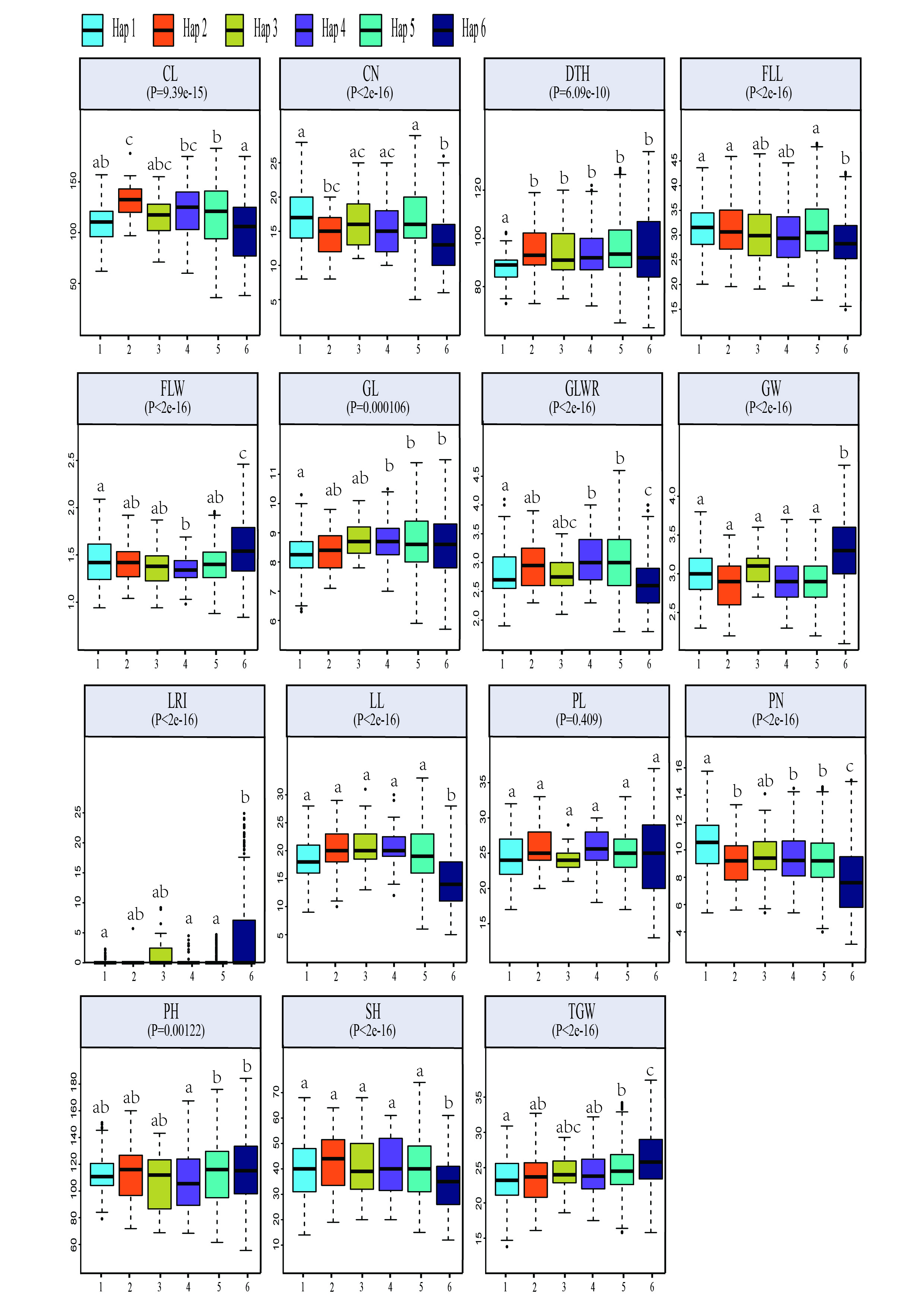

Supplement: Supplementary Figure 16 — Comparison and analysis of 15 agronomic traits among the predominant gcHap, unfavorable gcHap, and major gcHaps of OsCXE7.6. [file DataSheet1.zip › Supplementary Figure 16-30/Supplementary Figure 28 cxe9.3.jpg]

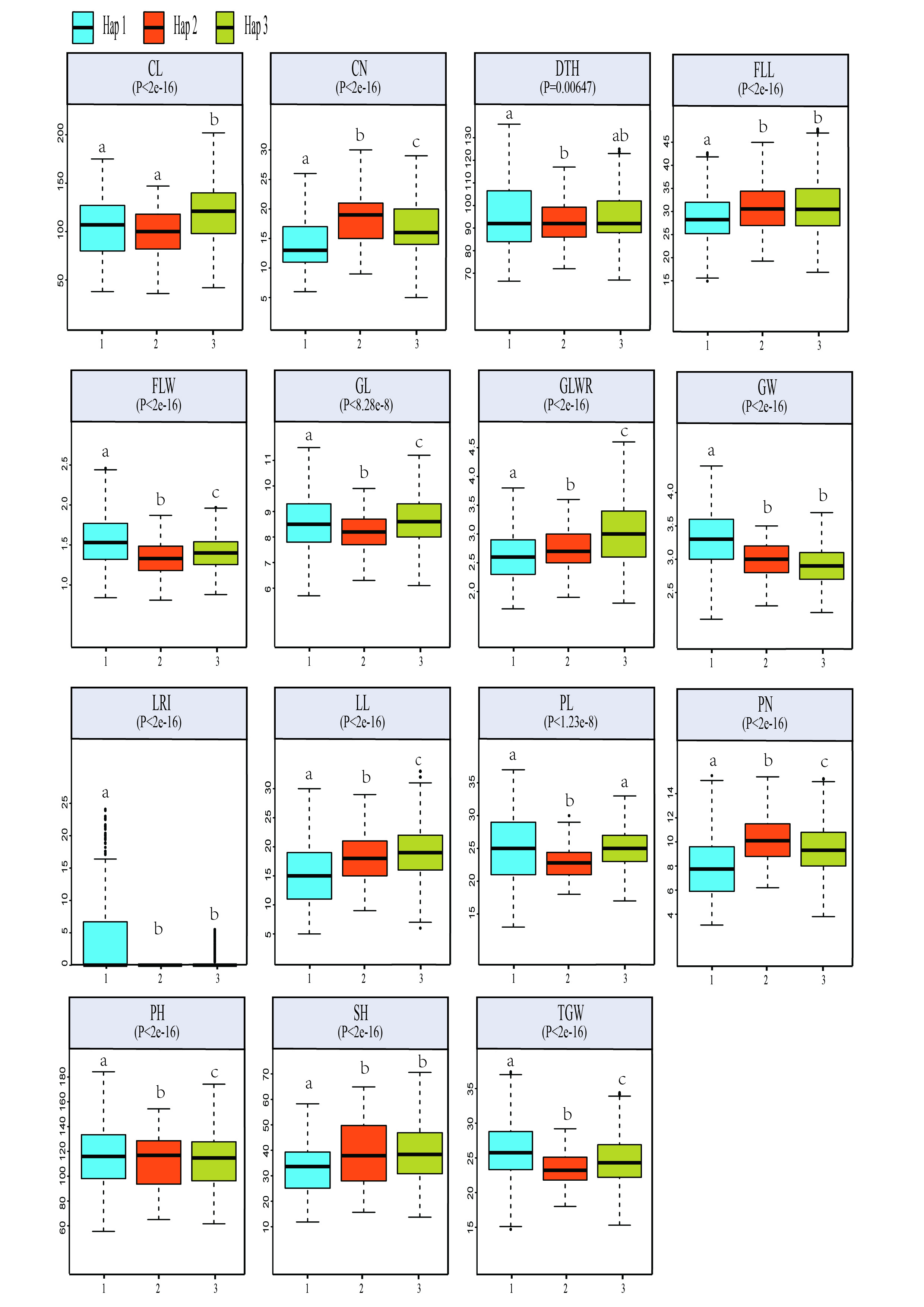

Supplement: Supplementary Figure 16 — Comparison and analysis of 15 agronomic traits among the predominant gcHap, unfavorable gcHap, and major gcHaps of OsCXE7.6. [file DataSheet1.zip › Supplementary Figure 16-30/Supplementary Figure 29 cxe9.4.jpg]

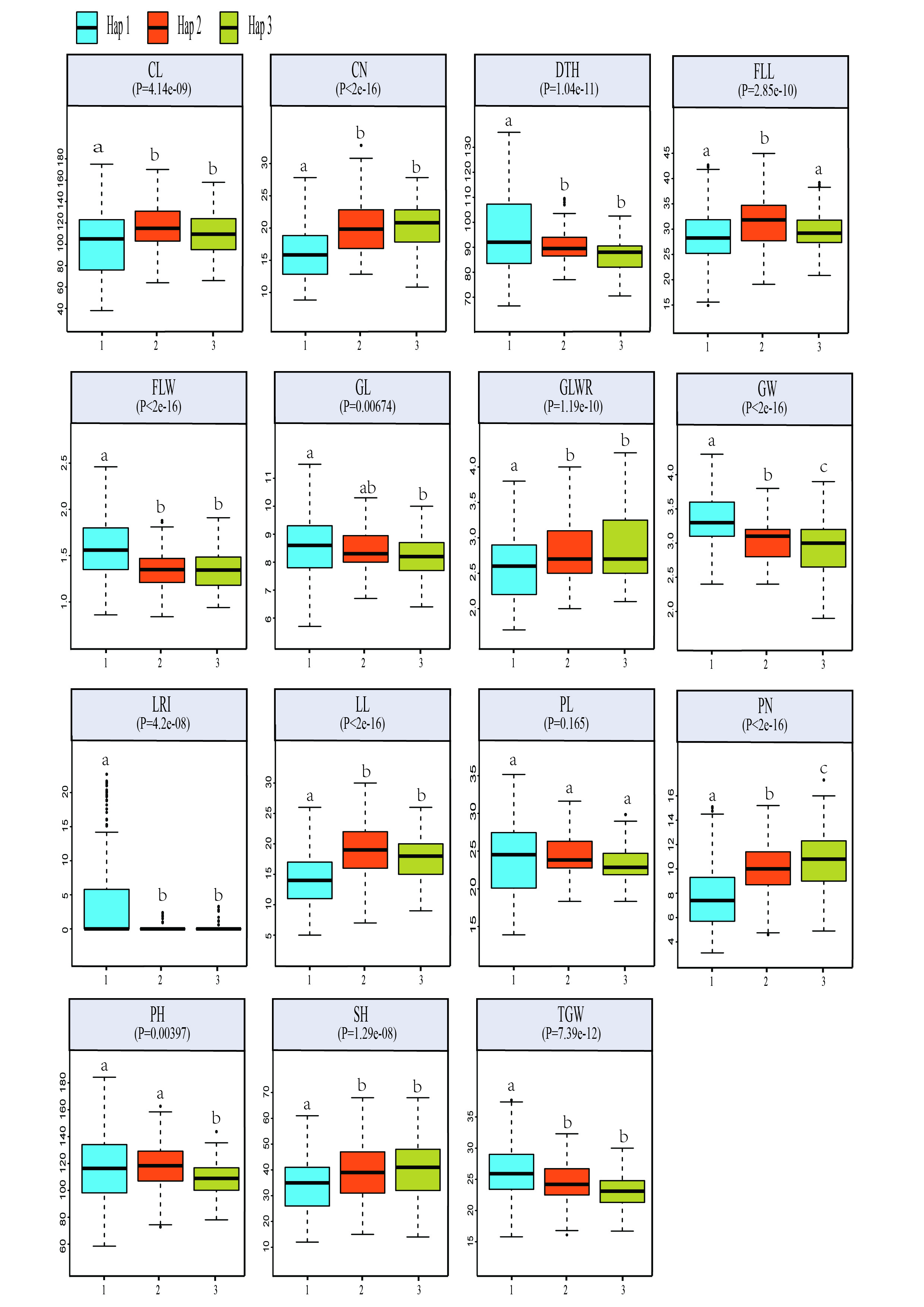

Supplement: Supplementary Figure 16 — Comparison and analysis of 15 agronomic traits among the predominant gcHap, unfavorable gcHap, and major gcHaps of OsCXE7.6. [file DataSheet1.zip › Supplementary Figure 16-30/Supplementary Figure 30 cxe11.1.jpg]

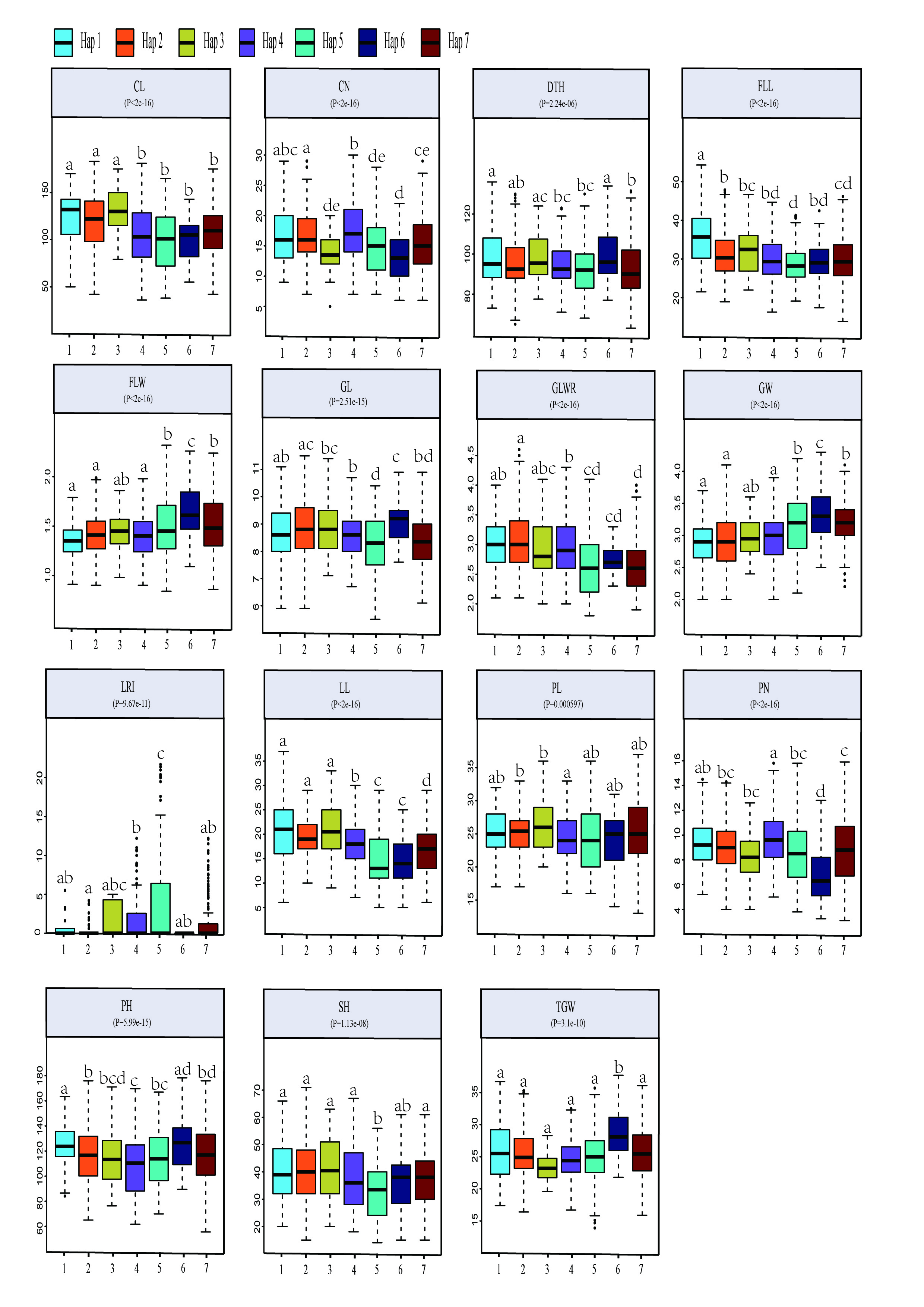

Supplement: Supplementary Figure 31 — Comparison and analysis of 15 agronomic traits among the predominant gcHap, unfavorable gcHap, and major gcHaps of OsCXE11.2. [file DataSheet2.zip › Supplementary Figure 31-41/Supplementary Figure 31 cxe11.2.jpg]

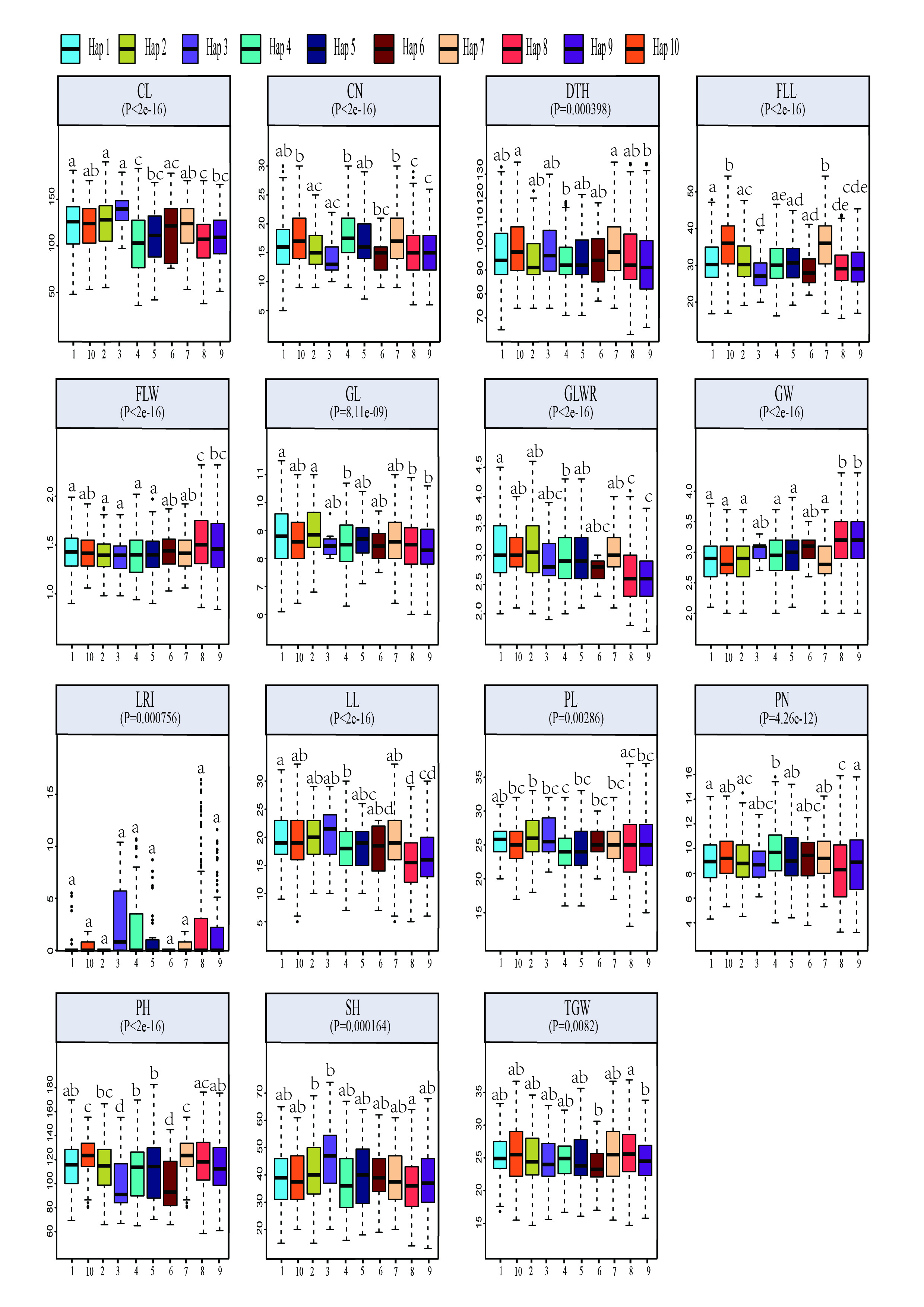

Supplement: Supplementary Figure 31 — Comparison and analysis of 15 agronomic traits among the predominant gcHap, unfavorable gcHap, and major gcHaps of OsCXE11.2. [file DataSheet2.zip › Supplementary Figure 31-41/Supplementary Figure 32 cxe11.3.jpg]

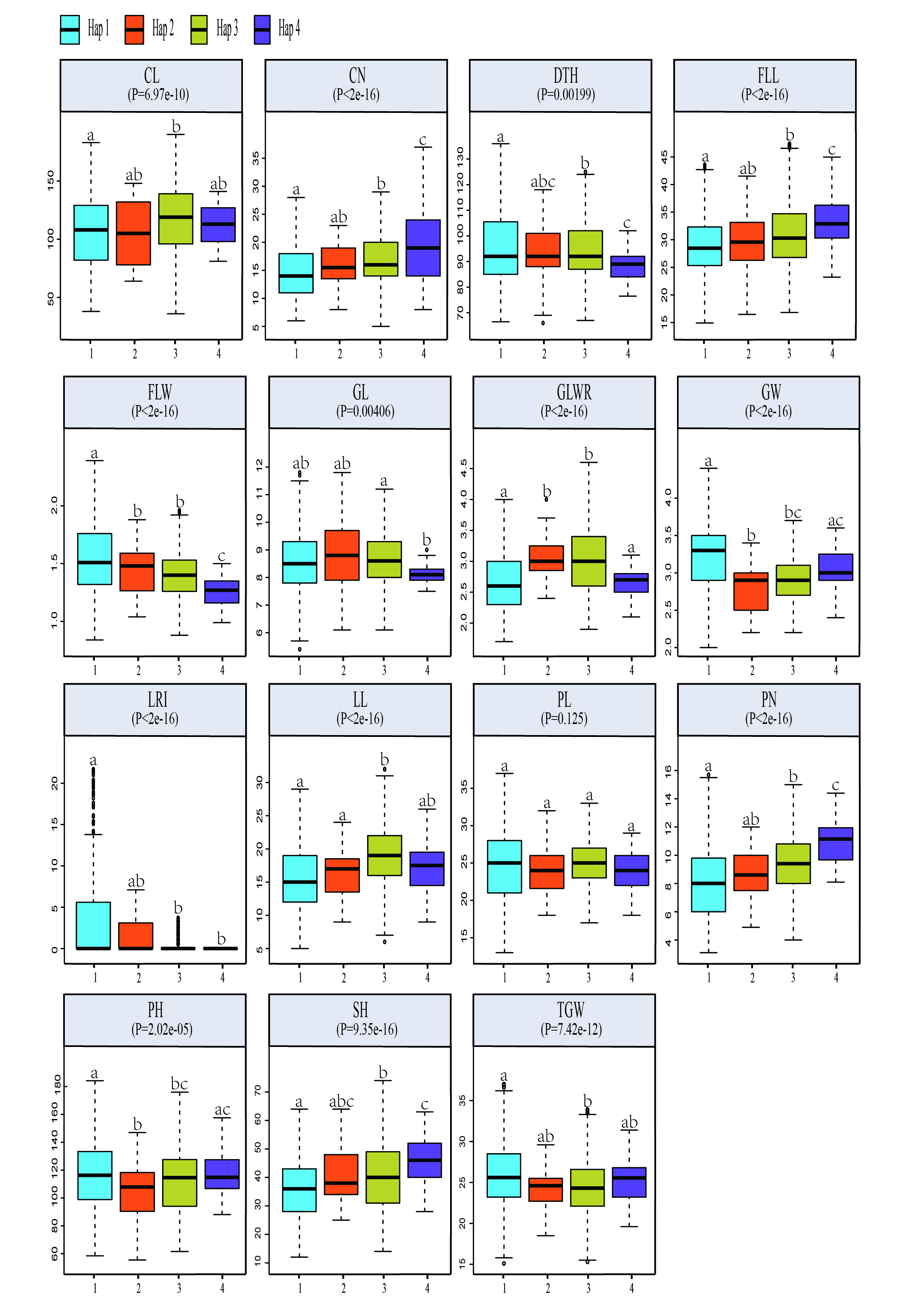

Supplement: Supplementary Figure 31 — Comparison and analysis of 15 agronomic traits among the predominant gcHap, unfavorable gcHap, and major gcHaps of OsCXE11.2. [file DataSheet2.zip › Supplementary Figure 31-41/Supplementary Figure 33 cxe12.1.jpg]

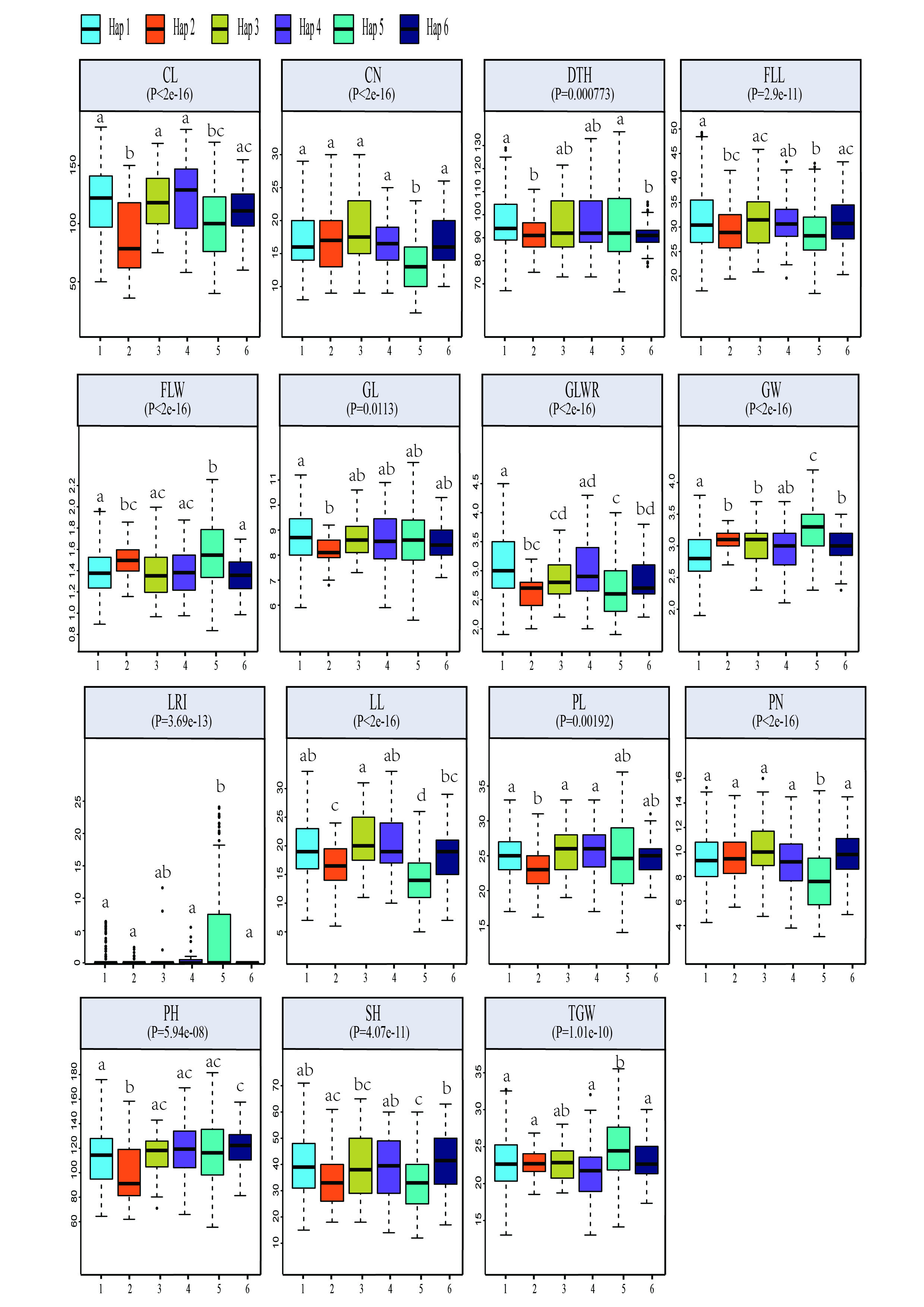

Supplement: Supplementary Figure 31 — Comparison and analysis of 15 agronomic traits among the predominant gcHap, unfavorable gcHap, and major gcHaps of OsCXE11.2. [file DataSheet2.zip › Supplementary Figure 31-41/Supplementary Figure 34 cxe12.2.jpg]

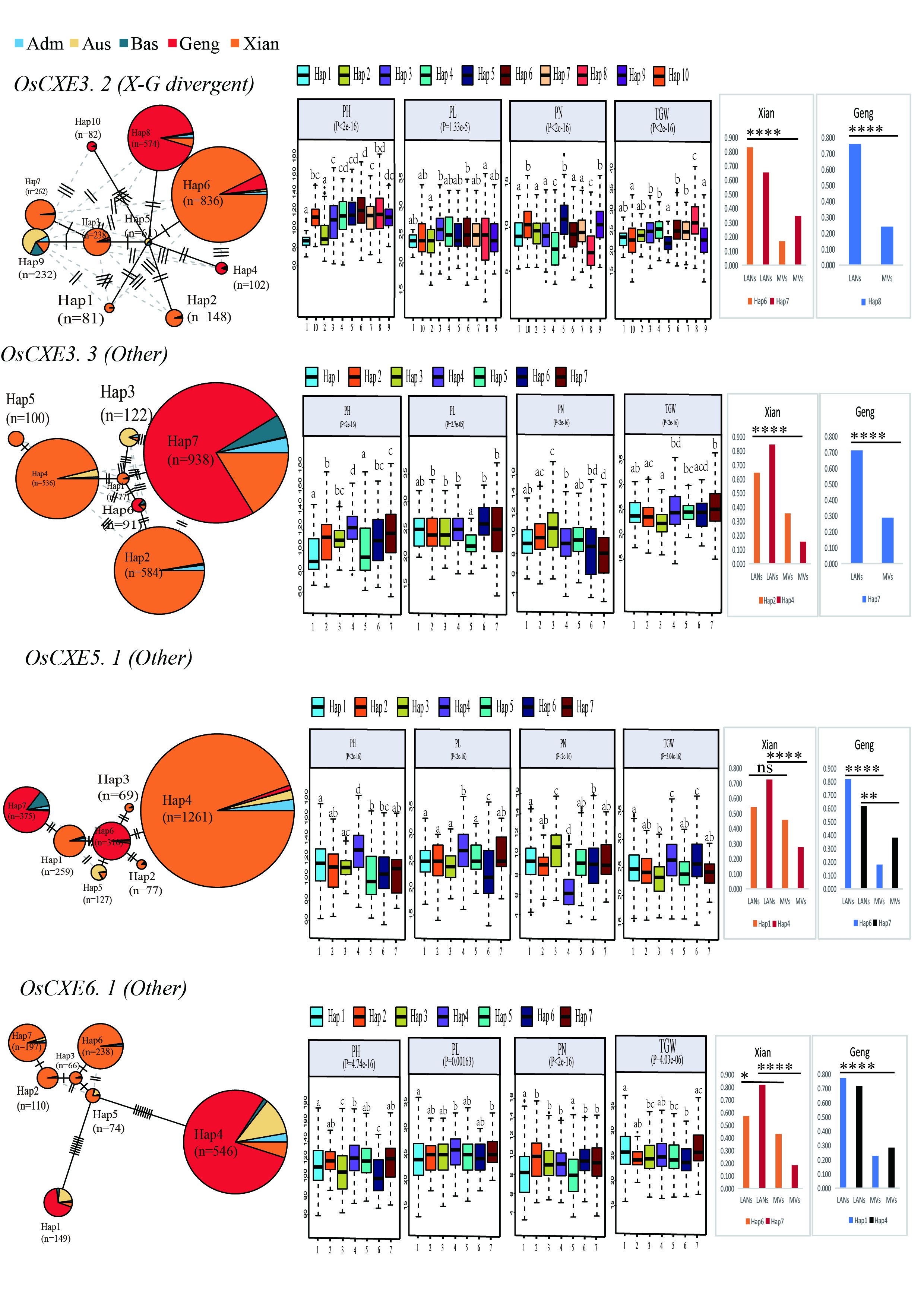

Supplement: Supplementary Figure 31 — Comparison and analysis of 15 agronomic traits among the predominant gcHap, unfavorable gcHap, and major gcHaps of OsCXE11.2. [file DataSheet2.zip › Supplementary Figure 31-41/Supplementary Figure 35 Haplotype networks of remaining OsCXE genes.jpg]

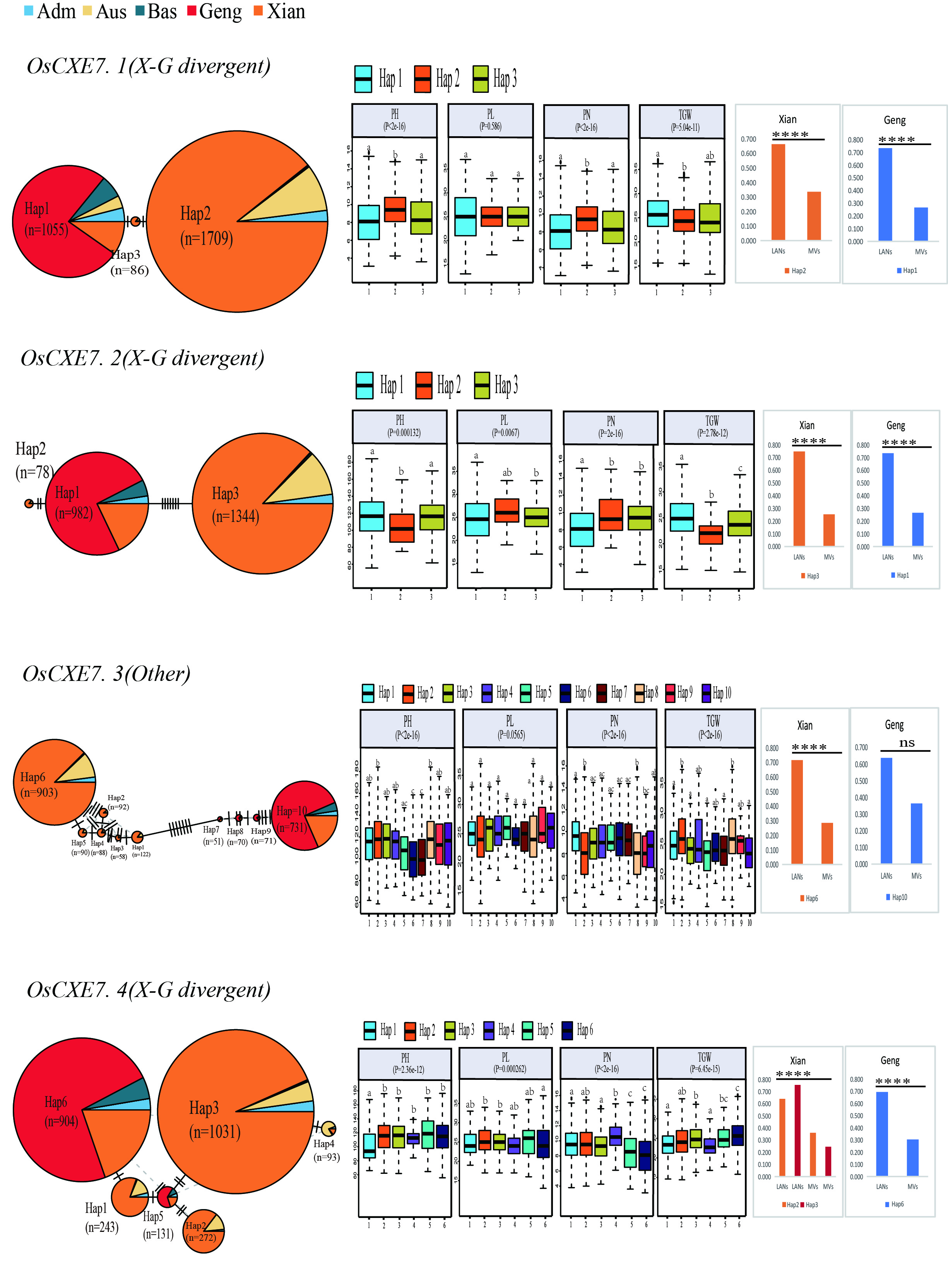

Supplement: Supplementary Figure 31 — Comparison and analysis of 15 agronomic traits among the predominant gcHap, unfavorable gcHap, and major gcHaps of OsCXE11.2. [file DataSheet2.zip › Supplementary Figure 31-41/Supplementary Figure 36 Haplotype networks of remaining OsCXE genes.jpg]

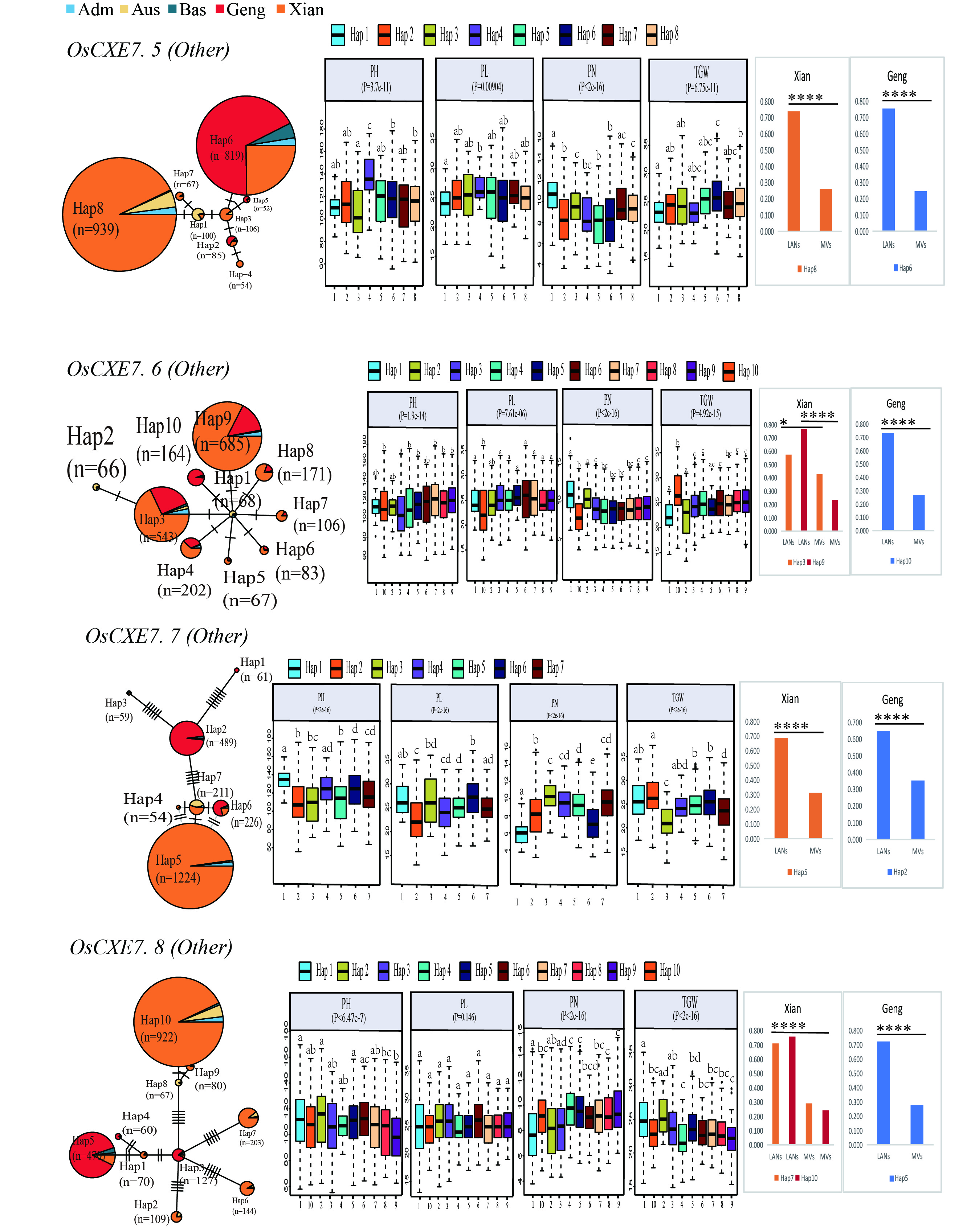

Supplement: Supplementary Figure 31 — Comparison and analysis of 15 agronomic traits among the predominant gcHap, unfavorable gcHap, and major gcHaps of OsCXE11.2. [file DataSheet2.zip › Supplementary Figure 31-41/Supplementary Figure 37 Haplotype networks of remaining OsCXE genes.jpg]

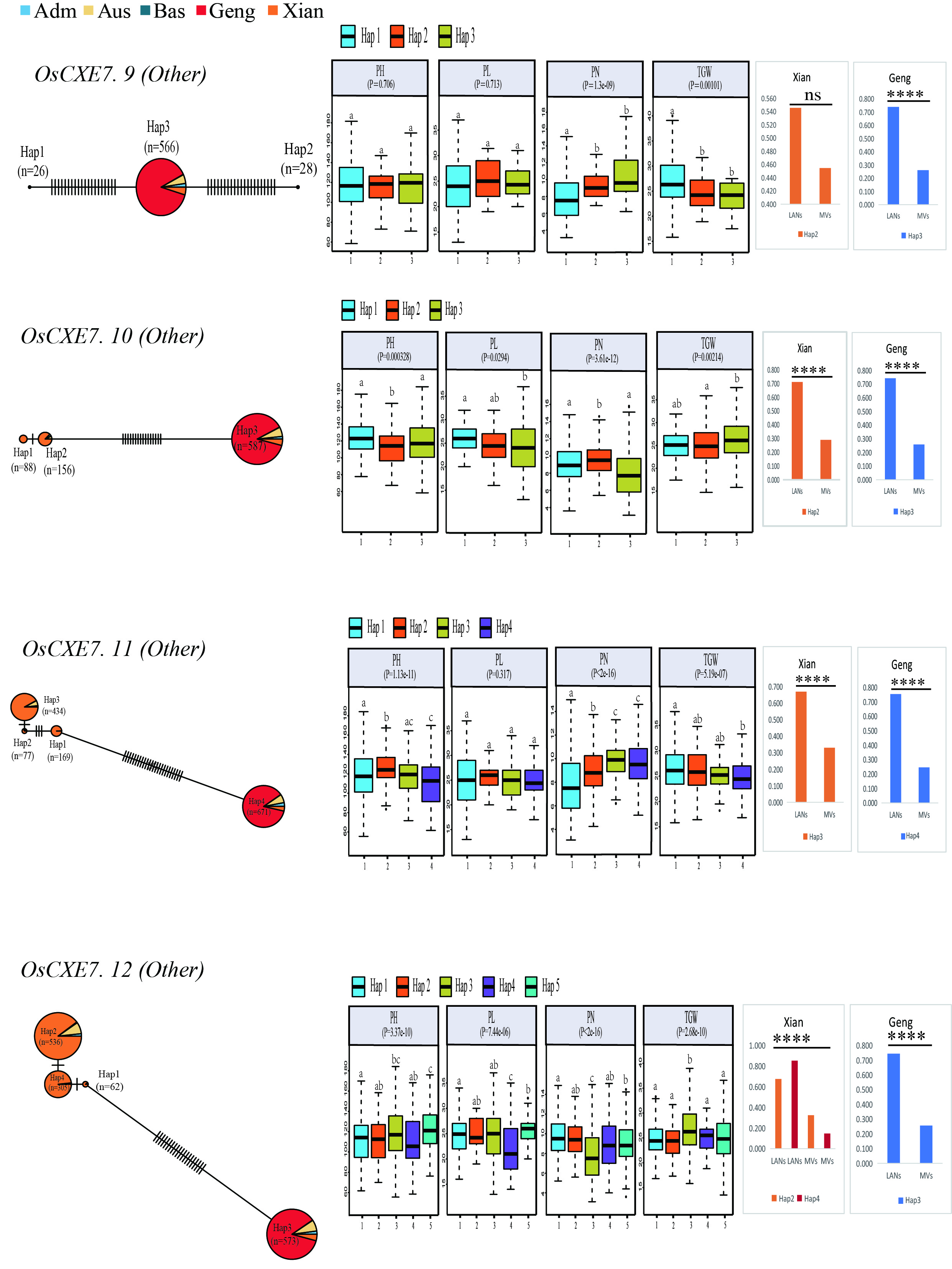

Supplement: Supplementary Figure 31 — Comparison and analysis of 15 agronomic traits among the predominant gcHap, unfavorable gcHap, and major gcHaps of OsCXE11.2. [file DataSheet2.zip › Supplementary Figure 31-41/Supplementary Figure 38 Haplotype networks of remaining OsCXE genes.jpg]

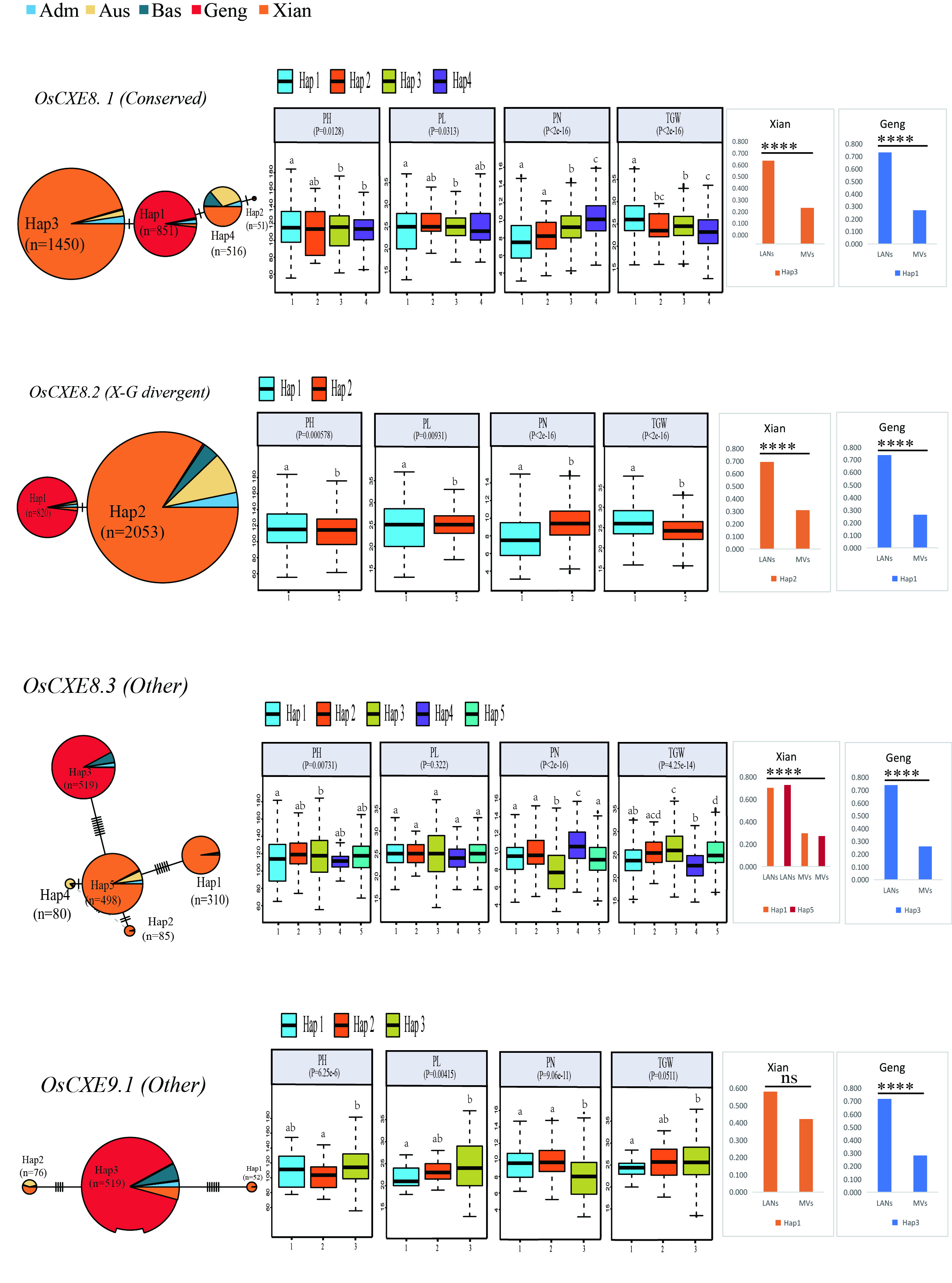

Supplement: Supplementary Figure 31 — Comparison and analysis of 15 agronomic traits among the predominant gcHap, unfavorable gcHap, and major gcHaps of OsCXE11.2. [file DataSheet2.zip › Supplementary Figure 31-41/Supplementary Figure 39Haplotype networks of remaining OsCXE genes.jpg]

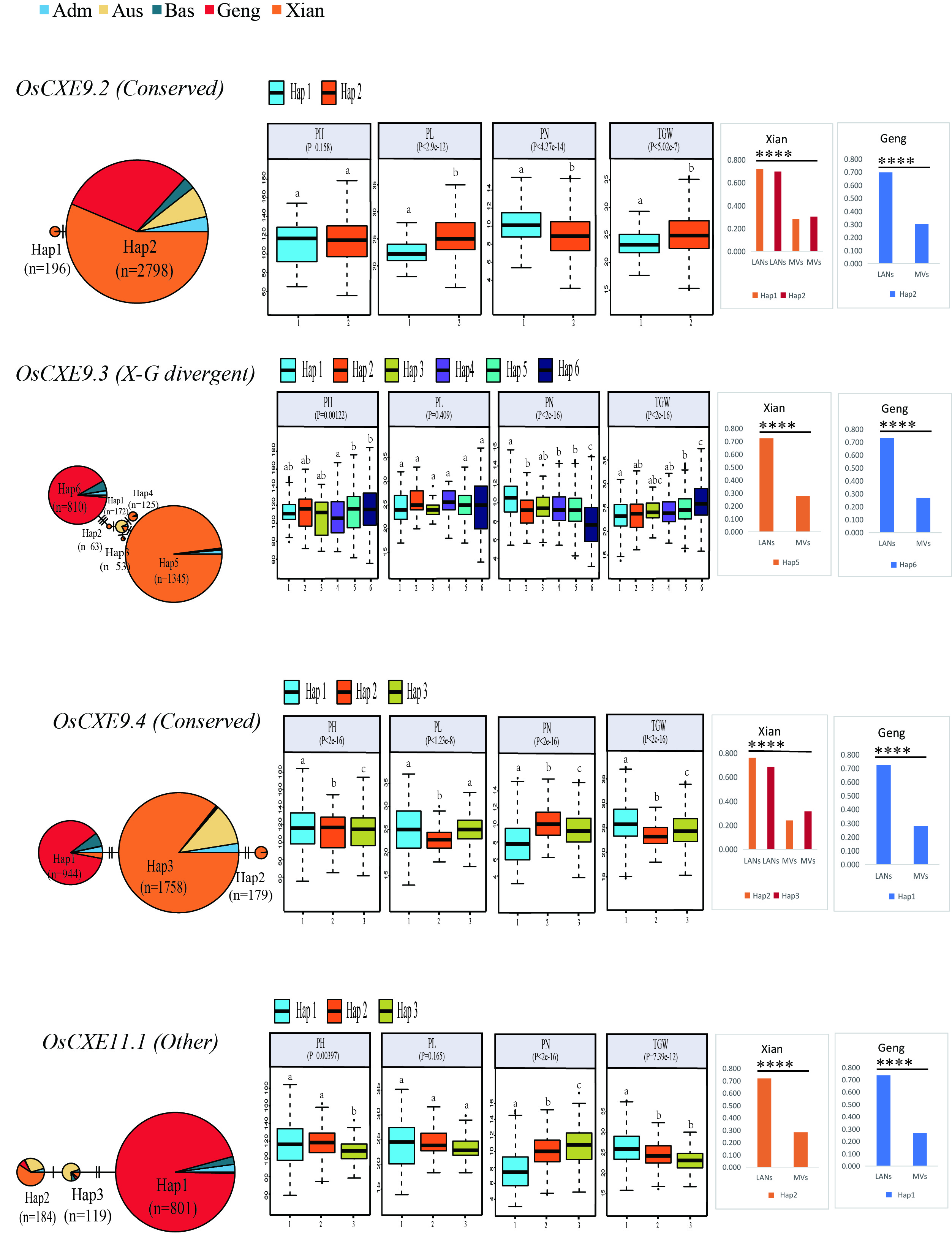

Supplement: Supplementary Figure 31 — Comparison and analysis of 15 agronomic traits among the predominant gcHap, unfavorable gcHap, and major gcHaps of OsCXE11.2. [file DataSheet2.zip › Supplementary Figure 31-41/Supplementary Figure 40 Haplotype networks of remaining OsCXE genes.jpg]

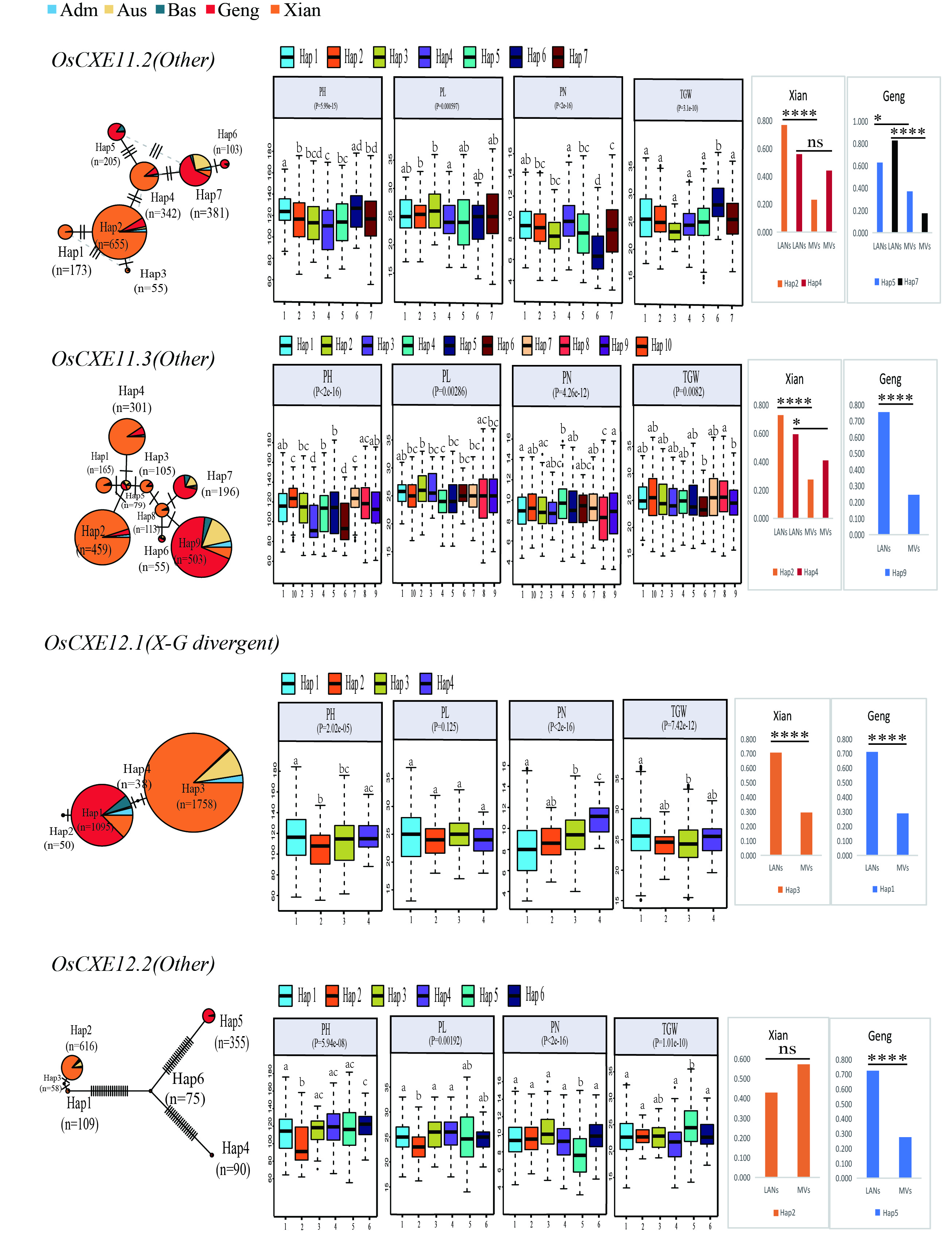

Supplement: Supplementary Figure 31 — Comparison and analysis of 15 agronomic traits among the predominant gcHap, unfavorable gcHap, and major gcHaps of OsCXE11.2. [file DataSheet2.zip › Supplementary Figure 31-41/Supplementary Figure 41Haplotype networks of remaining OsCXE genes.jpg]
